# Disparities in glycaemic control, monitoring, and treatment of type 2 diabetes in England: A retrospective cohort analysis

**DOI:** 10.1371/journal.pmed.1002942

**Published:** 2019-10-07

**Authors:** Martin B. Whyte, William Hinton, Andrew McGovern, Jeremy van Vlymen, Filipa Ferreira, Silvio Calderara, Julie Mount, Neil Munro, Simon de Lusignan

**Affiliations:** 1 Department of Clinical and Experimental Medicine, University of Surrey, Guildford, United Kingdom; 2 Eli Lilly, Basingstoke, United Kingdom; University of Manchester, UNITED KINGDOM

## Abstract

**Background:**

Disparities in type 2 diabetes (T2D) care provision and clinical outcomes have been reported in the last 2 decades in the UK. Since then, a number of initiatives have attempted to address this imbalance. The aim was to evaluate contemporary data as to whether disparities exist in glycaemic control, monitoring, and prescribing in people with T2D.

**Methods and findings:**

A T2D cohort was identified from the Royal College of General Practitioners Research and Surveillance Centre dataset: a nationally representative sample of 164 primary care practices (general practices) across England. Diabetes healthcare provision and glucose-lowering medication use between 1 January 2012 and 31 December 2016 were studied. Healthcare provision included annual HbA1c, renal function (estimated glomerular filtration rate [eGFR]), blood pressure (BP), retinopathy, and neuropathy testing. Variables potentially associated with disparity outcomes were assessed using mixed effects logistic and linear regression, adjusted for age, sex, ethnicity, and socioeconomic status (SES) using the Index of Multiple Deprivation (IMD), and nested using random effects within general practices. Ethnicity was defined using the Office for National Statistics ethnicity categories: White, Mixed, Asian, Black, and Other (including Arab people and other groups not classified elsewhere). From the primary care adult population (*n =* 1,238,909), we identified a cohort of 84,452 (5.29%) adults with T2D. The mean age of people with T2D in the included cohort at 31 December 2016 was 68.7 ± 12.6 years; 21,656 (43.9%) were female. The mean body mass index was 30.7 ± SD 6.4 kg/m^2^. The most deprived groups (IMD quintiles 1 and 2) showed poorer HbA1c than the least deprived (IMD quintile 5). People of Black ethnicity had worse HbA1c than those of White ethnicity. Asian individuals were less likely than White individuals to be prescribed insulin (odds ratio [OR] 0.86, 95% CI 0.79–0.95; *p <* 0.01), sodium-glucose cotransporter-2 (SGLT2) inhibitors (OR 0.68, 95% CI 0.58–0.79; *p <* 0.001), and glucagon-like peptide-1 (GLP-1) agonists (OR 0.37, 95% CI 0.31–0.44; *p <* 0.001). Black individuals were less likely than White individuals to be prescribed SGLT2 inhibitors (OR 0.50, 95% CI 0.39–0.65; *p <* 0.001) and GLP-1 agonists (OR 0.45, 95% CI 0.35–0.57; *p <* 0.001). Individuals in IMD quintile 5 were more likely than those in the other IMD quintiles to have annual testing for HbA1c, BP, eGFR, retinopathy, and neuropathy. Black individuals were less likely than White individuals to have annual testing for HbA1c (OR 0.89, 95% CI 0.79–0.99; *p =* 0.04) and retinopathy (OR 0.82, 95% CI 0.70–0.96; *p =* 0.011). Asian individuals were more likely than White individuals to have monitoring for HbA1c (OR 1.10, 95% CI 1.01–1.20; *p =* 0.023) and eGFR (OR 1.09, 95% CI 1.00–1.19; *p =* 0.048), but less likely for retinopathy (OR 0.88, 95% CI 0.79–0.97; *p =* 0.01) and neuropathy (OR 0.88, 95% CI 0.80–0.97; *p =* 0.01). The study is limited by the nature of being observational and defined using retrospectively collected data. Disparities in diabetes care may show regional variation, which was not part of this evaluation.

**Conclusions:**

Our findings suggest that disparity in glycaemic control, diabetes-related monitoring, and prescription of newer therapies remains a challenge in diabetes care. Both SES and ethnicity were important determinants of inequality. Disparities in glycaemic control and other areas of care may lead to higher rates of complications and adverse outcomes for some groups.

## Abstract 2

Martin Brunel Whyte and colleagues reveal disparities in diabetes monitoring and treatment for patients of different ethnicites in England.

## Author summary

**Why was this study done?:**

- There are few studies of ethnic disparities in diabetes care conducted in healthcare systems free at the point of delivery and with adjustment for socioeconomic status. These have generally shown overall improvement in diabetes management and outcomes over time, but with persistent imbalance between population groups.
- A number of initiatives have attempted to redress disparity of care in diabetes management in the UK over the last decade.

**What did the researchers do and find?:**

- We set out to determine whether there are healthcare disparities (inequalities between the sexes or across socioeconomic and ethnic groups) in the care of people with type 2 diabetes.
- We analysed routinely collected data from a network of primary care practices in England for measures of clinical outcomes, prescribing, and care processes for individuals with type 2 diabetes.
- Our study provides up-to-date information on care processes and metabolic outcomes for type 2 diabetes mellitus.
- We found disparities in glycaemic control, disease monitoring, and prescribing between ethnicities and socioeconomic groups, and a weak difference between the sexes.

**What do these findings mean?:**

- The commissioning of diabetes services must continue with the ambition to reduce inequalities and thereby improve population health for individuals with type 2 diabetes.

## Introduction

There are over 3.7 million people diagnosed with diabetes in the United Kingdom—a number that is increasing by about 5% per year [1]. Type 2 diabetes (T2D) accounts for 90% of these cases [1]. In parallel with this, the ethnic minority population of England and Wales has grown from 4.5 million in 2001 to 6.4 million in 2011 [2]. Seven percent of the population is of South Asian origin (Indian, Pakistani, Bangladeshi, or other Asian origin), 3.3% of African or Caribbean origin, and 2.2% of mixed origin. People from South Asian and Black ethnic groups are twice as likely to have diabetes as people from White or other ethnic groups (15.2% versus 8.0%, respectively) [1]. It is therefore important to be aware of the variations in disease manifestations and management in these individuals. In addition, those with low socioeconomic status (SES) are at higher risk of developing T2D [3]. The rise in T2D has been mirrored by rising obesity levels, which are also socially patterned [4]. The growing burden of T2D is therefore shared unequally across SES and between ethnicity groups [5].

The term ‘health disparities’ relates to preventable differences in the burden of disease between population groups. The causes of health disparities are complex and include societal issues such as poverty, marginalisation, discrimination, poor access to healthcare, and low education [6].

Diabetes doubles the risk of cardiovascular disease (heart attacks, heart failure, angina, and strokes) [7], is one of the most common, preventable, causes of blindness [8], and is the second most common reason for end stage kidney disease [9]. Diabetes is estimated to cost the UK £9 billion per annum; this equates to approximately 10% of the total health resource expenditure [10]. It is estimated that 80% of these costs are incurred in treating potentially avoidable complications [10,11].

As a societal aim is to achieve better health for the whole population, it is vital to reduce health inequalities. Since the turn of the century, a number of sources have highlighted disparity in diabetes outcomes in the UK, with individuals with T2D of Black African [5,12], Pakistani, or Indian heritage [5,13–15] having worse glycaemic control than their White counterparts, and those of low SES also having worse glucose regulation [5].

The concerning evidence of disparity in medical care over the last 40 years [16] has prompted a number of initiatives. In 2003, the UK government made reducing health inequality a key priority of national health policy [17]. This was supported by the national Quality and Outcomes Framework (QOF), introduced in 2004 [18] to provide financial incentives to general practitioners for monitoring and achieving control of glycaemia, cholesterol, and blood pressure (BP) levels. QOF also rewarded monitoring for disease complications such as retinopathy and neuropathy and urine examination for microalbuminuria. QOF was not introduced with the explicit aim of reducing health inequality, but its impact on inequality has been the subject of much speculation [19–21]. Universal quality improvement programmes, such as QOF, have been criticised for initially widening inequality (inverse equity hypothesis) [22]. Earlier reports in 2010 and 2012 suggested that QOF did not impact inequalities, either positively or negatively [19,23–26]. A study of inner London primary care practices found that following the introduction of QOF, glycated haemoglobin (HbA1c) control improved for all ethnic groups. However, people of South Asian and Black African/Caribbean ethnicity had persistently worse control than people of White ethnicity, despite more intensive treatment [12].

Although across the UK population as a whole, from 2004 to 2012, the National Health Service (NHS) was successful in improving the quality of diabetes healthcare, inequality remained unchanged [27].

In 2012, the Health and Social Care Act gave the NHS an explicit duty to consider reducing inequalities in healthcare outcomes [28], and more recently, the NHS 10-year plan, published in late 2018, explicitly includes the need to reduce inequalities [29]. These laudable aims have been tempered by the global macroeconomic situation. Health investment in the UK since 2010 has increased at lower rates than previously, with proportionally less being allocated to deprived areas [30].

Given these significant changes in fiscal and health policy since 2010, we have undertaken a contemporary analysis of whether social inequalities persist in diabetes management in primary care.

Studies of diabetes disparity tend to report indicators as either (i) clinical outcomes [27] or surrogate clinical markers [5,12,27,31] or (ii) care processes [32], but seldom both [19,21,23,26]. The primary objective of this study was to identify whether there are differences in glycaemic control (HbA1c) across socioeconomic and/or ethnic groups in the UK. We also investigated whether disparity exists in medication prescribing, monitoring of glycaemic control (HbA1c), and screening for diabetes complications, across socioeconomic groups, sex, and ethnicity.

## Methods

This was a retrospective cohort analysis of adults with T2D identified from the Royal College of General Practitioners Research and Surveillance Centre (RCGP RSC) database (S1 Text). This comprises primary care data from a sentinel network of primary care practices (general practices) distributed across England. The clinical computing systems that the contributing practices use are EMIS Web, INPS Vision, and TPP SystmOne. The database included all recorded clinical codes with associated values and dates for the population up to 31 December 2016. Clinical codes are recorded using the Read coding system and include diagnosis codes, medication codes, investigation codes, and process of care codes. This large primary care database has been demonstrated to be representative of the national population [33].

UK general practice is a registration-based system with residents registering with a single general practitioner. Care is free; nearly all care and prescribing for T2D is carried out in primary care. Patients with diabetes are also exempt from prescription charges.

We used a 2-step process to identify people with T2D, which we have reported in full previously [34,35]. In brief, the first step identifies all people with diabetes (of any type), defined as those who had a diagnostic code (diagnosis of diabetes), clinical investigations (2 or more fasted glucose, random glucose, or glucose tolerance test values or HbA1c measurements consistent with diagnosis), or medication use (2 or more prescriptions for oral diabetes medications, excluding metformin or injectable therapies). These people were then categorised by diabetes type using a clinically-based 7-step algorithm. We have found that this method reduces misdiagnosis, misclassification, and miscoding in people with diabetes [36,37]. All adults (age ≥ 18 years) with identified T2D prior to 2012, and with continuance in the RCGP RSC database over the period 2012–2016, were included for analysis.

Within the adult T2D population, we identified all medication prescriptions for sodium-glucose cotransporter-2 (SGLT2) inhibitors, glucagon-like peptide-1 (GLP-1) agonists, metformin, insulin, sulphonylureas, dipeptidyl peptidase-4 (DPP-4) inhibitors, and thiazolidinediones, between 1 January 2012 and 31 December 2016. We did not include fixed-dose combination therapies.

The 3 study outcomes (glycaemic control, disease monitoring, and prescribing) were assessed for association with sex, SES, and ethnicity.

### Definition of study outcomes

Disease monitoring was defined as visits that included assessment of glycaemic control, BP, renal function (estimated glomerular filtration rate [eGFR]), retinopathy, and neuropathy. Monitoring was identified using a combination of referral, diagnosis, examination, and clinical investigation codes (S2 Text) and was assessed over a 5-year time period (1 January 2012 to 31 December 2016). Unbroken annual monitoring for a variable was ‘complete annual monitoring’. Medication use was described as at least 1 prescription within the medication class recorded within 2012–2016. Most recently measured HbA1c was used as the measure of glycaemic control, i.e., the HbA1c value closest to the end of follow-up.

### Definition of exposure variables of interest

Ethnicity was defined using the Office for National Statistics official UK ethnicity categories, which define 5 major ethnic categories: White, Mixed/Multiple (e.g., White and Black Caribbean), Asian (including Indian, Pakistani, Bangladeshi, and Chinese), Black (including African and Caribbean), and Other (including Arab and other minority groups not classified elsewhere) [38]. SES was derived using the Index of Multiple Deprivation (IMD), based on patient postcode. We used an ontological approach to maximise our ability to detect ethnicity [39].

### Statistical analysis

We performed mixed effects regression analyses to determine the relationship between sex, socioeconomic group, and ethnicity and prescribing rates and diabetes monitoring (‘complete annual monitoring’ of glycaemic control, BP, renal function [eGFR], retinopathy, and neuropathy). All models included SES and ethnicity and were also adjusted for age and sex. In order to account for any tendency for ethnic minority groups to live in urbanised areas (which may be less affluent), for the main model we used mixed effects modelling with patients nested within general practices using a random intercept. This approach can also adjust for any practice-level differences in disease monitoring and prescribing.

Linear regression was performed to assess the association between exposure variables and glycaemic control (last HbA1c recorded in follow-up period), and logistic regression for prescribing and disease monitoring, with the binary outcome variables (i) at least 1 prescription of the medication class of interest in 2012–2016 (yes/no) and (ii) complete annual monitoring during the follow-up period (between 2012 and 2016).

People who died or deregistered during the follow-up period were excluded from the analysis. For missing data, we included a missing category for categorical variables and therefore included people with missing data. For continuous variables, people with missing data were excluded.

As a post hoc analysis, we also tested for an interaction effect between SES and ethnicity in the mixed effects model.

As sensitivity analyses, we have included the univariate models as well as a model adjusted for a larger number of potential confounding variables: age, sex, smoking, alcohol use, duration of diabetes, HbA1c, concurrent diabetes therapies, diabetes complications, comorbidities, BP, and body mass index (BMI) (S3–S5 Text).

Smoking status and alcohol use were defined using the most recently recorded measure. Duration of diabetes was defined as the time between the first recorded indicator of diabetes (diagnostic code, blood glucose measures consistent with diabetes, or medication) in the record and the date of data extraction (31 December 2016). BMI and BP were defined using the most recently recorded value. The presence of diabetes complications and comorbidities was determined by the presence of diagnosis codes or other codes specific for the diagnosis. The diabetes complications identified comprised amputation, peripheral neuropathy, retinopathy, and peripheral vascular disease. The comorbidities identified comprised hypertension, atrial fibrillation, angina, stroke, myocardial infarction, congestive cardiac failure, history of transient ischaemic attack, coronary artery disease, chronic kidney disease (CKD 3–5), renal replacement therapy (dialysis or transplant), dementia, depression, rheumatoid arthritis, and chronic liver disease (any cause). Chronic kidney disease was identified using eGFR measurements and diagnosis codes. All variables included were categorical to account for nonlinear relationships with the outcome measure and were retained in multivariate analyses irrespective of associations in univariate analyses. Complications and comorbidities were defined using codes for diagnosis, investigation, and process of care (S5 Text). Results are mean ± standard deviation unless otherwise stated. All analyses were performed using R statistical software version 3.5.3.

### Ethics

All data were pseudonymised at the point of data extraction. No clinically identifiable information was available to researchers. National Research Ethics Committee approval was obtained on 30 September 2016 (REF: 16/WM/0425), and the study was subsequently approved by the RCGP RSC approvals committee.

## Results

### The RCGP RSC population

The RCGP RSC database at the time of this study included a total of 1,595,170 people (adults and children) from 164 primary care practices. The mean age of the total RCGP RSC population was 39.6 ± 23.1 years, and 807,516 (50.6%) were female. People with a higher IMD were slightly over-represented in the population (S1 Fig). Patients’ ethnicity was identifiable for 1,182,883 (74.2% of the population).

### The diabetes cohort

Within the RCGP RSC population, from 1,238,909 adults, 90,730 were identified with diabetes. Of these, 84,452 (93.1%) had T2D. A priori we decided to include only those with diabetes diagnosed before 2012 and continuance in the RCGP RSC database for the 5-year period 2012–2016, leaving *n =* 49,380 (Table 1). The mean age of people with T2D in the included cohort at 31 December 2016 was 68.7 ± 12.6 years. Less than half of these were female (43.9%). Ethnicity was identified in 85.5% of the diabetes cohort. Asian (9.0%) and Black (4.1%) people were slightly over-represented in the population with T2D, relative to the general RCGP RSC population (5.8% and 3.5%, respectively).

**Table 1:** The characteristics of the type 2 diabetes mellitus adult population diagnosed before 2012 and with follow-up for the 5 years (*n =* 49,380).

Characteristic	*n* (%)
**Sex**	
Female	21,656 (43.9)
Male	27,724 (56.1)
**Ethnicity**	
White	35,008 (70.9)
Asian	4,422 (9.0)
Black	2,031 (4.1)
Mixed	378 (0.8)
Other	365 (0.7)
Missing	7,176 (14.5)
**Socioeconomic status**	
IMD quintile 1 (most deprived)	9,388 (19.0)
IMD quintile 2	8,692 (17.6)
IMD quintile 3	9,258 (18.7)
IMD quintile 4	10,379 (21.0)
IMD quintile 5 (least deprived)	11,571 (23.4)
Missing	92 (0.2)
**Body mass index**	
Underweight	272 (0.6)
Normal	7,886 (16.0)
Overweight	16,866 (34.2)
Obese class 1	13,468 (27.3)
Obese class 2	6,470 (13.1)
Obese class 3	3,899 (7.9)
Missing	519 (1.1)
**Smoking status**	
Never	11,931 (24.2)
Active	6,535 (13.2)
Ex-smoker	29,583 (59.9)
Missing	1,331 (2.7)
**Duration of diabetes**	
Under 5 years	20,256 (41.0)
5–9 years	16,593 (33.6)
10–14 years	7,868 (15.9)
15–19 years	2,866 (5.8)
≥20 years	1,797 (3.6)
**HbA1c**	
<53 mmol/mol (<7%)	23,044 (46.7)
53–63 mmol/mol (7%–8%)	13,412 (27.2)
64–74 mmol/mol (8%–9%)	6,182 (12.5)
75–85 mmol/mol (9%–10%)	3,122 (6.3)
≥86 mmol/mol (≥10%)	3,444 (7.0)
Missing	176 (0.4)
**Systolic blood pressure (mm Hg)**	
<120	7,504 (15.2)
120–139	27,938 (56.6)
140–159	11,703 (23.7)
≥160	2,169 (4.4)
Missing	66 (0.1)
**Diastolic blood pressure (mm Hg)**	
<80	34,289 (69.4)
80–89	12,404 (25.1)
90–99	2,206 (4.5)
≥100	415 (0.8)
Missing	66 (0.1)
**eGFR (ml/min/1.73 m**^**2**^**)**	
<15	230 (0.5)
15–29	975 (2.0)
30–44	3,374 (6.9)
45–59	5,897 (12.1)
≥60	38,244 (78.5)
Missing	660 (1.3)

eGFR, estimated glomerular filtration rate; IMD, Index of Multiple Deprivation.

The majority of the T2D cohort (*n =* 48,861; 98.9%) had 1 or more measurements of BMI. The mean BMI was 30.7 ± 6.4 kg/m^2^. Nearly all patients (*n =* 49,204; 99.6%) also had 1 or more HbA1c measurements available. The mean most recent HbA1c measurement for the cohort was 57.5 ± 16.5 mmol/mol (7.4% ± 1.5%).

### Disparities in glycaemic control

There was a clear gradient of worsening glycaemic control across socioeconomic groups, with the HbA1c of IMD quintile 1 being higher by 1.86 (95% CI 1.29–2.42) mmol/mol (*p <* 0.001) than that in the least deprived group (IMD quintile 5). Black people had worse glycaemic control (HbA1c +2.36 [95% CI 1.53–3.19] mmol/mol; *p <* 0.001) than White people, as did Asians (+1.10 [95% CI 0.49–1.71] mmol/mol; *p <* 0.001) (Table 2). These findings were supported by the sensitivity analyses (S1 Table).

**Table 2:** Effect of clinical characteristics on most recent HbA1c (mmol/mol), estimated from multivariate linear regression.

Characteristic	ß-coefficient	95% CI	*p-*Value
**Sex**			
Female	REF	REF	REF
Male	0.31	0.02 to 0.59	0.034
**Ethnicity**			
White	REF	REF	REF
Asian	1.10	0.49 to 1.71	<0.001
Black	2.36	1.53 to 3.19	<0.001
Mixed	0.04	−1.60 to 1.67	0.967
Other	0.87	−0.83 to 2.57	0.316
Missing	0.65	0.16 to 1.14	0.009
**IMD quintile**			
1 (most deprived)	1.86	1.29 to 2.42	<0.001
2	1.53	1.01 to 2.05	<0.001
3	1.20	0.72 to 1.68	<0.001
4	0.56	0.11 to 1.01	0.015
5 (least deprived)	REF	REF	REF
Missing	0.03	−3.44 to 3.49	0.988

IMD, Index of Multiple Deprivation.

### Disparities in monitoring

Healthcare monitoring of the diabetes cohort improved annually for HbA1c measurement (82.5% in 2012 to 92.8% in 2016), eGFR (83.3% in 2012 to 92.4% in 2016), and neuropathic testing (65.4% in 2012 to 72.2% in 2016). Monitoring of BP also improved (85.9% in 2012 to 92.9% in 2015) with the exception of 2016 (91.3%). For retinopathy, however, monitoring rates peaked in 2013 (68.6% overall) and have fallen annually since, to 59.5% overall in 2016.

Complete annual monitoring (2012–2016) for HbA1c (69.0%), eGFR (66.1%), and BP (71.5%) was found in the majority of the cohort, whilst just over a quarter of the cohort had complete annual monitoring for presence of neuropathy (27.8%) and retinopathy (25.9%) (S2 Table).

Males were more likely than females to have complete annual monitoring (2012–2016) for HbA1c (odds ratio [OR] 1.06, 95% CI 1.02–1.11; *p =* 0.002) and neuropathy screening (OR 1.05, 95% CI 1.01–1.10; *p =* 0.019), but the effect sizes were small (Table 3).

**Table 3:** Mixed effects model for disparities in monitoring of glycaemic control and complications.

Characteristic	HbA1c monitoring	Blood pressure monitoring	eGFR monitoring	Retinal screening	Neuropathy screening
**Sex**					
Female	REF	REF	REF	REF	REF
Male	1.06 (1.02–1.11)**	1.01 (0.97–1.05)	1.01 (0.97–1.05)	1.04 (1.00–1.09)	1.05 (1.01–1.10)*
**Ethnicity**					
White	REF	REF	REF	REF	REF
Asian	1.10 (1.01–1.20)*	1.00 (0.91–1.09)	1.09 (1.00–1.19)*	0.88 (0.79–0.97)*	0.88 (0.80–0.97)*
Black	0.89 (0.79–0.99)*	0.93 (0.82–1.05)	0.90 (0.80–1.01)	0.82 (0.70–0.96)*	0.98 (0.85–1.13)
Mixed	0.80 (0.65–1.00)*	0.88 (0.70–1.11)	0.89 (0.72–1.11)	0.73 (0.54–0.97)*	0.94 (0.73–1.21)
Other	0.90 (0.72–1.13)	0.75 (0.59–0.94)*	0.92 (0.73–1.15)	0.63 (0.47–0.84)**	0.89 (0.67–1.20)
Missing	0.62 (0.58–0.66)***	0.55 (0.51–0.59)***	0.63 (0.59–0.67)***	0.80 (0.74–0.87)***	0.69 (0.64–0.75)***
**IMD quintile**					
1 (most deprived)	0.77 (0.71–0.84)***	0.85 (0.78–0.93)***	0.88 (0.81–0.96)**	0.75 (0.68–0.83)***	0.80 (0.73–0.88)***
2	0.84 (0.78–0.91)***	0.89 (0.82–0.97)**	0.92 (0.86–0.99)*	0.85 (0.78–0.92)***	0.87 (0.80–0.94)***
3	0.85 (0.79–0.91)***	0.89 (0.83–0.96)**	0.92 (0.86–0.99)*	0.89 (0.82–0.96)**	0.89 (0.83–0.96)**
4	0.88 (0.82–0.93)***	0.88 (0.82–0.94)***	0.96 (0.90–1.02)	0.86 (0.81–0.92)***	0.90 (0.84–0.96)**
5 (least deprived)	REF	REF	REF	REF	REF
Missing	0.59 (0.37–0.95)*	0.65 (0.39–1.08)	0.66 (0.41–1.06)	0.36 (0.17–0.75)**	0.53 (0.32–0.89)*

Data are odds ratio (95% CI). Model adjusted for age, sex, ethnicity, and socioeconomic status, with patients additionally nested within primary care practices.

*p < 0.05

**p < 0.01

***p < 0.001.

eGFR, estimated glomerular filtration rate; IMD, Index of Multiple Deprivation.

People belonging to the least deprived socioeconomic group (IMD quintile 5) were significantly more likely than the other IMD quintiles to have complete annual monitoring (2012–2016) for HbA1c, BP, eGFR, retinopathy, and neuropathy (Table 3).

Black people were less likely than White people to have complete annual monitoring for HbA1c (OR 0.89, 95% CI 0.79–0.99; *p =* 0.04) and presence of retinopathy (OR 0.82, 95% CI 0.70–0.96; *p =* 0.011). Asian individuals were more likely than White individuals to have complete annual monitoring for HbA1c (OR 1.10, 95% CI 1.01–1.20; *p =* 0.023) and eGFR (OR 1.09, 95% CI 1.00–1.19; *p =* 0.048), but less likely to have retinopathy screening (OR 0.88, 95% CI 0.79–0.97; *p =* 0.01) and neuropathy screening (OR 0.88, 95% CI 0.80–0.97; *p =* 0.01). These relationships were also apparent in the univariate models and were unchanged in the fully adjusted model (adjusted for age, sex, BP, BMI, smoking, alcohol, duration of diabetes, HbA1c level, concurrent diabetes therapies, diabetes complications, and comorbidities) in the sensitivity analyses (S1 Table).

Similarly, the sensitivity analyses showed that people belonging to the more deprived socioeconomic groups (IMD quintiles 1–4) remained less likely to receive complete annual monitoring than those in the least deprived group (IMD quintile 5), across all test types. For sex, the sensitivity analyses did not support the (small) effect that had been observed, of greater monitoring in males.

Testing for an interaction effect of SES and ethnicity showed that Asian individuals in IMD quintile 1 (most deprived) were more likely to have monitoring for HbA1c, BP, retinopathy, and neuropathy than predicted by ethnicity and IMD alone. No other substantial interactions were discernible, and there was no interaction between Asian ethnicity and any of the other IMD quintiles.

### Disparities in prescribing

**Fig 1:**
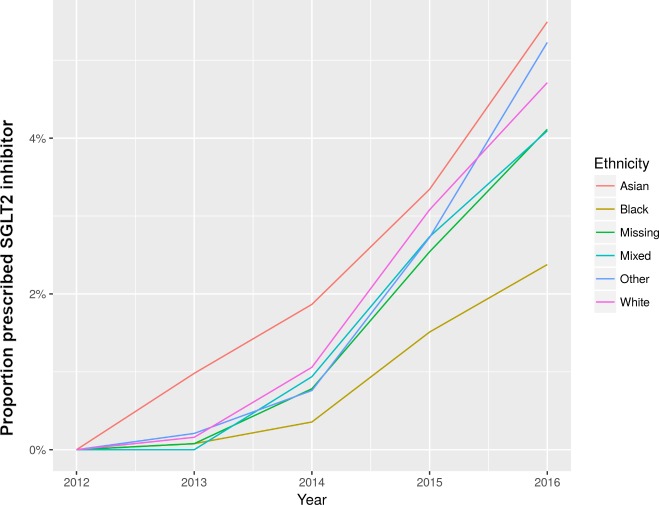
Proportion of ethnic groups prescribed SGLT2 inhibitors for type 2 DM, by year of prescription. DM, diabetes mellitus.

Metformin was the most commonly prescribed drug, prescribed at least once during 2012–2016 in 79.2% of the cohort, followed by sulphonylureas (42.4%) and DPP-4 inhibitors (26.4%). Insulin was prescribed in 20.0% of the cohort, and GLP-1 agonists in 3.4%. SGLT2 inhibitors have shown gradual rise in usage since licencing in November 2012 (Fig 1).

Those in the most deprived groups (IMD quintiles 1 and 2) were more likely to be prescribed insulin (OR 1.21, 95% CI 1.11–1.32, *p <* 0.001, and OR 1.18, 95% CI 1.09–1.28, *p <* 0.001, respectively), metformin (OR 1.20, 95% CI 1.09–1.31, *p <* 0.001, and OR 1.19, 95% CI 1.10–1.29, *p <* 0.001), sulphonylureas (OR 1.12, 95% CI 1.05–1.21, *p =* 0.001, and OR 1.10, 95% CI 1.03–1.18, *p =* 0.005), and DPP-4 inhibitors (OR 1.17, 95% CI 1.07–1.28, *p <* 0.001, and OR 1.11, 95% CI 1.02–1.20, *p =* 0.01) than those in the least deprived group (IMD quintile 5). Compared to the least deprived group (IMD quintile 5), GLP-1 prescribing was more likely in IMD quintiles 1, 3, and 4; however, this effect was lost in the sensitivity analyses (S1 Table). There were no patterns of difference in SGLT2 inhibitor prescribing across IMD quintiles (Table 4).

**Table 4:** Mixed effects model for disparities in prescribing.

Characteristic	Insulin	Metformin	Sulphonylurea	DPP-4 inhibitor	GLP-1 agonist	SGLT2 inhibitor
**Sex**						
Female	REF	REF	REF	REF	REF	REF
Male	0.93 (0.89–0.97)**	1.21 (1.16–1.27)***	1.21 (1.17–1.25)***	1.08 (1.03–1.12)***	0.82 (0.77–0.88)***	1.00 (0.93–1.08)
**Ethnicity**						
White	REF	REF	REF	REF	REF	REF
Asian	0.86 (0.79–0.95)**	1.67 (1.49–1.87)***	1.29 (1.19–1.39)***	1.10 (1.01–1.20)*	0.37 (0.31–0.44)***	0.68 (0.58–0.79)***
Black	1.05 (0.93–1.18)	1.27 (1.09–1.48)**	1.18 (1.07–1.31)**	0.97 (0.86–1.09)	0.45 (0.35–0.57)***	0.50 (0.39–0.65)***
Mixed	0.96 (0.75–1.24)	1.36 (1.01–1.85)*	1.21 (0.99–1.49)	0.92 (0.73–1.17)	0.38 (0.23–0.62)***	0.69 (0.44–1.06)
Other	1.11 (0.86–1.44)	1.45 (1.04–2.03)*	1.27 (1.02–1.58)*	1.17 (0.93–1.47)	0.42 (0.26–0.69)***	0.71 (0.46–1.09)
Missing	0.98 (0.91–1.05)	0.91 (0.85–0.98)*	0.94 (0.88–1.00)	0.94 (0.87–1.01)	0.82 (0.73–0.93)**	0.90 (0.79–1.03)
**IMD quintile**						
1 (most deprived)	1.21 (1.11–1.32)***	1.20 (1.09–1.31)***	1.12 (1.05–1.21)**	1.17 (1.07–1.28)***	1.15 (1.00–1.33)*	1.09 (0.95–1.27)
2	1.18 (1.09–1.28)***	1.19 (1.10–1.29)***	1.10 (1.03–1.18)**	1.11 (1.02–1.20)*	1.09 (0.96–1.25)	1.06 (0.92–1.21)
3	1.07 (0.99–1.15)	1.12 (1.04–1.20)**	1.08 (1.02–1.15)*	1.06 (0.99–1.14)	1.14 (1.01–1.29)*	0.97 (0.85–1.10)
4	1.05 (0.98–1.13)	1.05 (0.98–1.12)	0.99 (0.94–1.05)	1.05 (0.98–1.13)	1.13 (1.01–1.27)*	1.12 (1.00–1.27)
5 (least deprived)	REF	REF	REF	REF	REF	REF
Missing	0.96 (0.54–1.71)	1.06 (0.61–1 .83)	1.00 (0.64–1.57)	0.94 (0.56–1.58)	0.48 (0.15–1.58)	0.73 (0.28–1.93)

Data are odds ratio (95% CI). Model adjusted for age, sex, ethnicity, and socioeconomic status, with patients additionally nested within primary care practices.

*p < 0.05

**p < 0.01

***p < 0.00.

IMD, Index of Multiple Deprivation.

**Fig 2:**
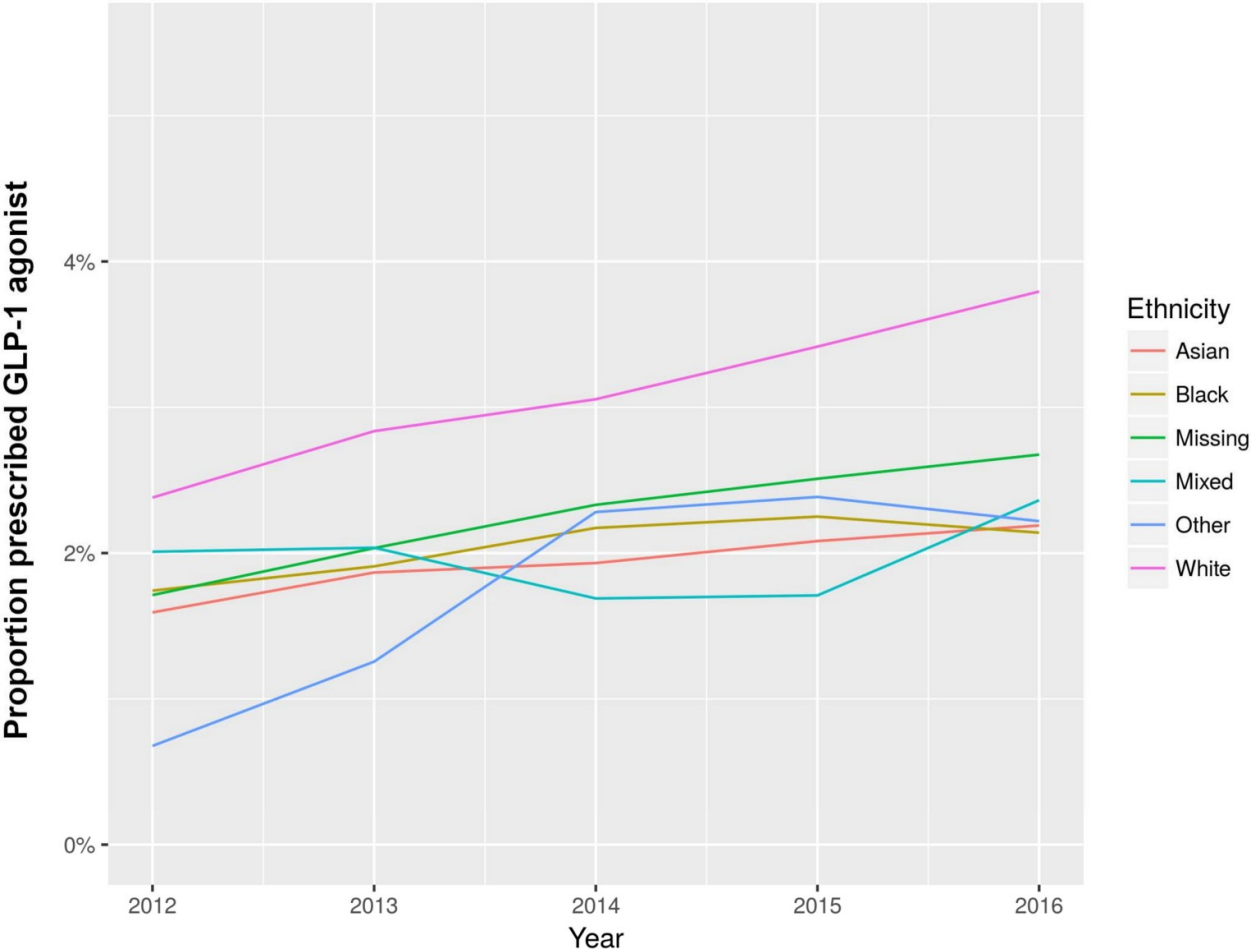
Proportion of ethnic groups prescribed GLP-1 agonists for type 2 DM, by year of prescription. DM, diabetes mellitus.

Asian people were more likely than White people to be prescribed metformin (OR 1.67, 95% CI 1.49–1.87; *p <* 0.001), sulphonylureas (OR 1.29, 95% CI 1.19–1.39; *p <* 0.001), and DPP-4 inhibitors (OR 1.10, 95% CI 1.01–1.20; *p =* 0.03). However, they were less likely to be prescribed insulin (OR 0.86, 95% CI 0.79–0.95; *p =* 0.002), GLP-1 agonists (OR 0.37, 95% CI 0.31–0.44; *p <* 0.001), and SGLT2 inhibitors (OR 0.68, 95% CI 0.58–0.79; *p <* 0.001). Black people were more likely than White people to be prescribed metformin (OR 1.27, 95% CI 1.09–1.48; *p =* 0.002) and sulphonylureas (OR 1.18, 95% CI 1.07–1.31; *p =* 0.002), but they were less likely to be prescribed SGLT2 inhibitors (OR 0.50, 95% CI 0.39–0.65; *p <* 0.001; Fig 1) and GLP-1 agonists (OR 0.45, 95% CI 0.35–0.57; *p <* 0.001; Fig 2). These relationships did not change with sensitivity analysis (S1 Table).

## Discussion

Our results show that both SES and ethnicity are important determinants of disparity in diabetes care. Black and Asian people, as well as those in the most socioeconomically disadvantaged group, had worse glycaemic control than White people. Disparities in monitoring for glycaemic control and complications were also seen, with individuals from the most deprived quintile of SES and from ethnic minority groups having less monitoring of HbA1c, eGFR, retinopathy, and neuropathy, although no disparity for monitoring of BP was found. We also found that there was relatively less prescribing of the (newer) GLP-1 agonists and SGLT2 inhibitors in Black and Asian individuals than in White individuals.

### Glycaemic control

As expected, diabetes was more prevalent among males, as well as people of Black and Asian ethnicities [5]. Glycaemic control was not so well achieved in the most socioeconomically disadvantaged individuals, as reported in earlier studies [12,32,40]. The implication of our finding is that although primary care practices may be achieving QOF targets, this could be without addressing inequality in care and hence mortality outcomes [19]. Disparity in glycaemic outcomes between ethnicities was not seen in analysis of the National Diabetes Audit (NDA) data up to 2015 [41], in which service organisation was of most importance in determining outcomes. However, practices that participated in the NDA were significantly more likely to be based in areas of low deprivation [42]. The NDA data treated glycated haemoglobin as a categorical variable (HbA1c <7.5% and >10%), whereas our analysis used HbA1c as a continuous outcome.

### Monitoring

The more deprived quintiles of SES had less monitoring of HbA1c, BP, eGFR, retinopathy, and neuropathy. Our data support the inverse equity hypothesis, whereby new services are accessed first by wealthy and well-informed individuals [43], leading to an initial widening of the inequality gap before it later narrows. Following the first year of QOF, achievements were higher in affluent areas [44]. An analysis of 34 QOF clinical indicator scores over the first 3 years suggested that the gap between practices in deprived and affluent areas narrowed significantly over this time period. After 3 years of QOF, a report suggested that a socioeconomic gradient in practice performance was no longer apparent [45]. However, our data do not support this. The Diabetes Prevention Programme (introduced in 2016) may prove to reduce inequality by helping to identify people at risk of diabetes or with existing (but undiagnosed) diabetes. It is too early to assess the effectiveness of this programme, but our data support, from an inequality perspective, the rationale for this intervention.

Black individuals were less likely than White individuals to have uninterrupted monitoring (over 5 years) of HbA1c and retinopathy, although they had equivalent monitoring of BP (after adjustment for SES and other factors). Equity in monitoring of BP may reflect a clinical awareness of the risk of hypertension in individuals of Black African/Caribbean ethnicity [46]. Whether hypertension is considered by the Black population a greater health risk than diabetes requires investigation. However, a study in south London has shown that whilst frequency of monitoring of BP may be adequate in Black individuals, the level of control achieved remains worse than in their White counterparts [47].

Recording of retinal screening was lower in ethnic minority groups and in those with lower SES. Minority ethnic communities with T2D in the UK have previously been shown to have low uptake of retinal screening and are more prone to diabetic retinopathy—including sight-threatening retinopathy and maculopathy—compared to the White population [48]. However, that analysis did not adjust for glycaemic control, BP, and duration of diabetes. Whether less participation in screening programmes directly translates into greater prevalence of retinopathy in minority populations merits further study.

Cost and accessibility may be limiting factors for screening attendance, and strategies are required to improve access for low SES groups [49]. Conversely, screening for diabetic neuropathy is based largely on symptoms and physical examination as part of the annual clinical review. Asian individuals had equivalent monitoring of BP and better monitoring of HbA1c and eGFR than the White population, but slightly less monitoring of retinopathy and neuropathy. This is important, as despite a lower unadjusted prevalence of clinical neuropathy in South Asian individuals compared with individuals of White or Black African/Caribbean ethnicity, people of Asian background with T2D in the UK are at greater risk of painful diabetic neuropathy [50]. It is noteworthy that Asian individuals in IMD quintile 1 (most deprived) were more likely to have testing for HbA1c, BP, retinopathy, and neuropathy than predicted by ethnicity and IMD alone. Further work is needed to determine the reason for this positive finding, but it offers hope that disparity in care may be overcome.

### Prescribing

Our data suggest disparity between ethnicities in the use of GLP-1 agonists and SGLT2 inhibitors—with less prescribing in Black and Asian individuals. The GLP-1 agonist and SGLT2 inhibitor classes confer significant cardiovascular protection, and so inequality in prescription of these agents would be a concern [51–54]. The disparities observed do not necessarily imply inequality or unfairness in prescribing: contraindications to use of these medications, such as the presence of renal impairment, may contribute. However, in our sensitivity analysis we used multiple regression with a range of metabolic and clinical characteristics to adjust for known confounders, which did not significantly change the data. Furthermore, we did not see a similar relationship for the other insulin-sensitising drugs: metformin and DPP-4 inhibitors.

### Strengths and limitations

The large size of the cohort and the robust nature of the diabetes case finding and algorithm for classification are major advantages of our approach. The algorithm we developed to facilitate ethnicity identification resulted in the identification of ethnic group for 85.6% of the T2D cohort [33]. This compares favourably with ethnicity identification in people with T2D in other large UK datasets (QResearch, 75.0%; Clinical Practice Research Datalink, 44.5%) [55]. As we have previously reported [33], the RCGP RSC network closely aligns with the national population in terms of age, ethnicity, and SES—save for a slight preponderance of individuals in the highest socioeconomic quintile (least deprived). Whilst this is important to acknowledge, the large cohort size means that the sample of people in the most deprived deciles is still large. Our analyses were of individuals in the RCGP RSC database over the time period 2012–2016. This may lead to bias as monitoring of individuals who left the RCGP RSC over this time period may be worse than reported.

The cohort is also limited by the nature of being observational and defined using retrospectively collected data. Whilst this puts significant constraints on the availability of data, data completeness for most clinically important variables was excellent. Disparities in diabetes care may show regional variation [56], which was not part of this evaluation, nor can international concordance be assumed.

## Conclusions

To summarise, we have found that disparities exist in glycaemic control and the use of more recently introduced medications for glycaemic control in ethnic minority groups. Socioeconomically disadvantaged individuals, as well as those of Black ethnicity, were less likely to have continuous monitoring for diabetes complications over the study period. Work is needed to determine whether these disparities represent inequality in care.

## Supporting information

S1 Fig The proportion of people from the RCGP RSC cohort with Index of Multiple Deprivation scores in each decile of deprivation (based on the national average). (DOCX)

S1 Table Sensitivity analyses. (PDF)

S2 Table The numbers (proportion) of people with annual data for 0, 1, 2, 3, 4, or 5 years, for each monitoring variable. (DOCX)

S1 Text Analysis plan. (DOCX)

S2 Text Referral, diagnosis, examination, and clinical investigation codes to identify disease monitoring. (DOCX)

S3 Text Potential confounding variables adjusted for in model of disease monitoring and prescribing rates. (DOCX)

S4 Text Codes for alcohol misuse. (PDF)

S5 Text Complications and comorbidities defined using codes for diagnosis, investigation, and process of care. (DOCX)

S6 Text STROBE checklist. (DOCX)

## Abbreviations

BMI: body mass index
BP: blood pressure
eGFR: estimated glomerular filtration rate
IMD: Index of Multiple Deprivation
NHS: National Health Service
OR: odds ratio
QOF: Quality and Outcomes Framework
RCGP RSC: Royal College of General Practitioners Research and Surveillance Centre
SES: socioeconomic status
T2D: type 2 diabetes
