# Supplementary material for: Disparities in glycaemic control, monitoring, and treatment of type 2 diabetes in England: A retrospective cohort analysis
Source: PLoS Med. 2019 Oct 7;16(10):e1002942. doi: 10.1371/journal.pmed.1002942 (PMC6779242; doi:10.1371/journal.pmed.1002942)
Supplement: S1 Fig — (DOCX) [file pmed.1002942.s001.docx]

**S6 Appendix**


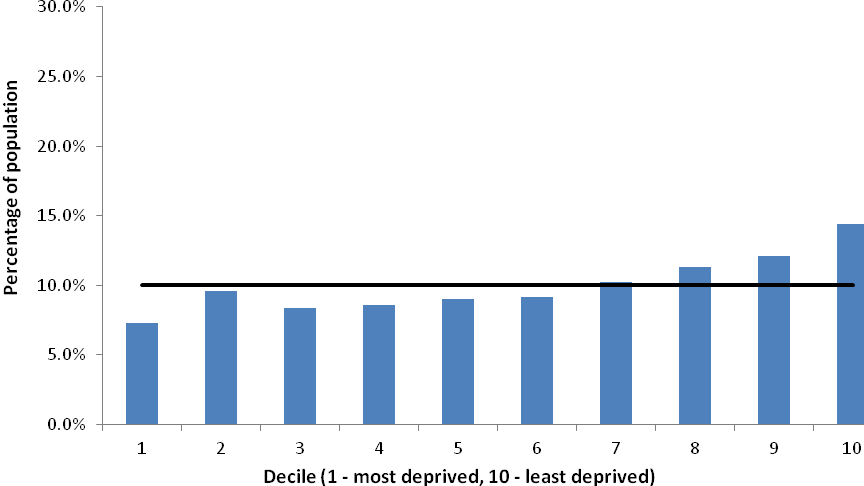


The proportion of people from the RCGP RSC cohort with index of multiple deprivation scores in each decile of deprivation (based on the national average).
