# Supplementary material for: Disparities in glycaemic control, monitoring, and treatment of type 2 diabetes in England: A retrospective cohort analysis
Source: PLoS Med. 2019 Oct 7;16(10):e1002942. doi: 10.1371/journal.pmed.1002942 (PMC6779242; doi:10.1371/journal.pmed.1002942)
Supplement: S1 Table — (PDF) [file pmed.1002942.s002.pdf]

	Uninterupted HbA1c monitoring (2012-2016)		
	Univariate models	Fully adjusted model*	Fully adjusted mixed effects model**
**Gender**			
Female		1.00 (reference)	
Male	1.03 (0.99 - 1.07)	0.94 (0.90 - 0.98)	1.06 (1.02 - 1.11)
**Ethnicity**			
White		1.00 (reference)	
Asian	0.81 (0.76 - 0.87)	1.01 (0.93 - 1.09)	1.10 (1.01 - 1.20)
Black	0.63 (0.58 - 0.69)	0.89 (0.81 - 0.99)	0.89 (0.79 - 0.99)
Missing	0.71 (0.67 - 0.75)	0.83 (0.78 - 0.88)	0.62 (0.58 - 0.66)
Mixed	0.59 (0.48 - 0.73)	0.75 (0.60 - 0.95)	0.80 (0.65 - 1.00)
Other	0.58 (0.47 - 0.71)	0.76 (0.61 - 0.95)	0.90 (0.72 - 1.13)
**Socioeconomic status**			
IMD quintile 1 (most deprived)	0.71 (0.67 - 0.76)	0.76 (0.71 - 0.81)	0.77 (0.71 - 0.84)
IMD quintile 2	0.78 (0.73 - 0.83)	0.84 (0.79 - 0.90)	0.84 (0.78 - 0.91)
IMD quintile 3	0.88 (0.83 - 0.94)	0.92 (0.86 - 0.98)	0.85 (0.79 - 0.91)
IMD quintile 4	0.88 (0.83 - 0.93)	0.88 (0.83 - 0.94)	0.88 (0.82 - 0.93)
IMD quintile 5 (least)		1.00 (reference)	
None	0.72 (0.47 - 1.12)	0.90 (0.56 - 1.45)	0.59 (0.37 - 0.95)

	Uninterupted BP monitoring (2012-2016)		
	Univariate models	Fully adjusted model*	Fully adjusted mixed effects model**
**Gender**			
Female		1.00 (reference)	
Male	0.97 (0.93 - 1.01)	0.86 (0.82 - 0.90)	1.01 (0.97 - 1.05)
**Ethnicity**			
White		1.00 (reference)	
Asian	0.82 (0.76 - 0.88)	1.11 (1.03 - 1.21)	1.00 (0.91 - 1.09)
Black	0.72 (0.66 - 0.79)	1.07 (0.95 - 1.19)	0.93 (0.82 - 1.05)
Missing	0.51 (0.48 - 0.54)	0.79 (0.74 - 0.84)	0.55 (0.51 - 0.59)
Mixed	0.70 (0.56 - 0.87)	0.95 (0.74 - 1.21)	0.88 (0.70 - 1.11)
Other	0.52 (0.42 - 0.65)	0.74 (0.59 - 0.93)	0.75 (0.59 - 0.94)
**Socioeconomic status**			
IMD quintile 1 (most deprived)	0.85 (0.80 - 0.91)	0.88 (0.82 - 0.94)	0.85 (0.78 - 0.93)
IMD quintile 2	0.84 (0.79 - 0.89)	0.89 (0.83 - 0.96)	0.89 (0.82 - 0.97)
IMD quintile 3	0.93 (0.87 - 0.98)	0.95 (0.89 - 1.02)	0.89 (0.83 - 0.96)
IMD quintile 4	0.91 (0.86 - 0.97)	0.88 (0.82 - 0.94)	0.88 (0.82 - 0.94)
IMD quintile 5 (least)		1.00 (reference)	
None	0.92 (0.59 - 1.48)	1.06 (0.63 - 1.76)	0.65 (0.39 - 1.08)

	Uninterupted eGFR monitoring (2012-2016)		
	Univariate models	Fully adjusted model*	Fully adjusted mixed effects model**
**Gender**			
Female		1.00 (reference)	
Male	0.97 (0.93 - 1.00)	0.91 (0.87 - 0.95)	1.01 (0.97 - 1.05)
**Ethnicity**			
White		1.00 (reference)	
Asian	0.77 (0.72 - 0.82)	1.04 (0.96 - 1.12)	1.09 (1.00 - 1.19)
Black	0.58 (0.53 - 0.64)	0.84 (0.76 - 0.93)	0.90 (0.80 - 1.01)
Missing	0.67 (0.64 - 0.71)	0.75 (0.71 - 0.80)	0.63 (0.59 - 0.67)
Mixed	0.62 (0.51 - 0.76)	0.83 (0.67 - 1.04)	0.89 (0.72 - 1.11)
Other	0.57 (0.47 - 0.70)	0.81 (0.65 - 1.01)	0.92 (0.73 - 1.15)
**Socioeconomic status**			
IMD quintile 1 (most deprived)	0.78 (0.74 - 0.83)	0.87 (0.81 - 0.92)	0.88 (0.81 - 0.96)
IMD quintile 2	0.86 (0.81 - 0.92)	0.95 (0.89 - 1.02)	0.92 (0.86 - 0.99)
IMD quintile 3	0.94 (0.89 - 1.00)	0.99 (0.93 - 1.05)	0.92 (0.86 - 0.99)
IMD quintile 4	0.95 (0.89 - 1.00)	0.96 (0.90 - 1.02)	0.96 (0.90 - 1.02)
IMD quintile 5 (least)		1.00 (reference)	
None	0.87 (0.57 - 1.36)	1.10 (0.69 - 1.77)	0.66 (0.41 - 1.06)

	Uninterupted retinal screening (2012-2016)		
	Univariate models	Fully adjusted model*	Fully adjusted mixed effects model**
**Gender**			
Female		1.00 (reference)	
Male	1.04 (1.00 - 1.09)	0.97 (0.93 - 1.02)	1.04 (1.00 - 1.09)
**Ethnicity**			
White		1.00 (reference)	
Asian	0.78 (0.72 - 0.84)	0.92 (0.845 - 0.994)	0.88 (0.79 - 0.97)
Black	0.43 (0.37 - 0.48)	0.54 (0.47 - 0.62)	0.82 (0.70 - 0.96)
Missing	0.81 (0.76 - 0.86)	0.92 (0.86 - 0.97)	0.80 (0.74 - 0.87)
Mixed	0.56 (0.43 - 0.73)	0.66 (0.507 - 0.870)	0.73 (0.54 - 0.97)
Other	0.60 (0.46 - 0.78)	0.75 (0.57 - 0.99)	0.63 (0.47 - 0.84)
**Socioeconomic status**			
IMD quintile 1 (most deprived)	0.61 (0.57 - 0.65)	0.68 (0.63 - 0.73)	0.75 (0.68 - 0.83)
IMD quintile 2	0.72 (0.67 - 0.77)	0.79 (0.743 - 0.848)	0.85 (0.78 - 0.92)
IMD quintile 3	0.70 (0.65 - 0.74)	0.73 (0.68 - 0.78)	0.89 (0.82 - 0.96)
IMD quintile 4	0.91 (0.86 - 0.96)	0.92 (0.87 - 0.98)	0.86 (0.81 - 0.92)
IMD quintile 5 (least)		1.00 (reference)	
None	0.25 (0.11 - 0.46)	0.26 (0.13 - 0.52)	0.36 (0.17 - 0.75)

	Uninterupted neuropathy screening (2012-2016)		
	Univariate models	Fully adjusted model*	Fully adjusted mixed effects model**
**Gender**			
Female		1.00 (reference)	
Male	1.03 (0.99 - 1.07)	0.98 (0.94 - 1.03)	1.05 (1.01 - 1.10)
**Ethnicity**			
White		1.00 (reference)	
Asian	0.77 (0.72 - 0.83)	0.88 (0.82 - 0.96)	0.88 (0.80 - 0.97)
Black	0.51 (0.46 - 0.57)	0.65 (0.58 - 0.74)	0.98 (0.85 - 1.13)
Missing	0.61 (0.57 - 0.65)	0.66 (0.62 - 0.70)	0.69 (0.64 - 0.75)
Mixed	0.73 (0.57 - 0.92)	0.87 (0.68 - 1.11)	0.94 (0.73 - 1.21)
Other	0.50 (0.38 - 0.65)	0.62 (0.47 - 0.81)	0.89 (0.67 - 1.20)
**Socioeconomic status**			
IMD quintile 1 (most deprived)	0.73 (0.69 - 0.78)	0.82 (0.77 - 0.87)	0.80 (0.73 - 0.88)
IMD quintile 2	0.69 (0.64 - 0.73)	0.75 (0.71 - 0.81)	0.87 (0.80 - 0.94)
IMD quintile 3	0.85 (0.80 - 0.90)	0.90 (0.84 - 0.95)	0.89 (0.83 - 0.96)
IMD quintile 4	0.91 (0.86 - 0.96)	0.92 (0.87 - 0.98)	0.90 (0.84 - 0.96)
IMD quintile 5 (least)		1.00 (reference)	
None	0.82 (0.51 - 1.28)	0.92 (0.57 - 1.48)	0.53 (0.32 - 0.89)

* Adjusted for x,y,z (variables described in the manuscript/Appendix)
** Model adjusted for age, sex, ethnicity, and SES only; with patients additionally nested within primary care practices

	Insulin		
	Univariate models	Fully adjusted model*	Fully adjusted mixed effects model**
**Gender**			
Female		1.00 (reference)	
Male	0.95 (0.91 - 0.99)	0.88 (0.828 - 0.925)	0.93 (0.89 - 0.97)
**Ethnicity**			
White		1.00 (reference)	
Asian	0.96 (0.89 - 1.04)	0.67 (0.61 - 0.74)	0.86 (0.79 - 0.95)
Black	1.23 (1.11 - 1.37)	0.98 (0.86 - 1.11)	1.05 (0.93 - 1.18)
Missing	0.98 (0.92 - 1.04)	0.92 (0.85 - 0.99)	0.98 (0.91 - 1.05)
Mixed	1.09 (0.85 - 1.39)	0.97 (0.73 - 1.30)	0.96 (0.75 - 1.24)
Other	1.16 (0.90 - 1.48)	0.98 (0.74 - 1.31)	1.11 (0.86 - 1.44)
**Socioeconomic status**			
IMD quintile 1 (most deprived)	1.22 (1.14 - 1.31)	1.02 (0.94 - 1.10)	1.21 (1.11 - 1.32)
IMD quintile 2	1.20 (1.12 - 1.29)	1.03 (0.95 - 1.12)	1.18 (1.09 - 1.28)
IMD quintile 3	1.09 (1.01 - 1.16)	0.97 (0.89 - 1.05)	1.07 (0.99 - 1.15)
IMD quintile 4	1.07 (1.00 - 1.14)	1.01 (0.94 - 1.10)	1.05 (0.98 - 1.13)
IMD quintile 5 (least)		1.00 (reference)	
None	0.86 (0.48 - 1.46)	0.74 (0.39 - 1.42)	0.96 (0.54 - 1.71)

	Metformin		
	Univariate models	Fully adjusted model*	Fully adjusted mixed effects model**
**Gender**			
Female		1.00 (reference)	
Male	1.28 (1.23 - 1.34)	1.24 (1.18 - 1.30)	1.21 (1.16 - 1.27)
**Ethnicity**			
White		1.00 (reference)	
Asian	2.34 (2.12 - 2.58)	1.94 (1.74 - 2.16)	1.67 (1.49 - 1.87)
Black	1.86 (1.64 - 2.13)	1.49 (1.29 - 1.72)	1.27 (1.09 - 1.48)
Missing	0.91 (0.86 - 0.97)	1.75 (1.28 - 2.38)	0.91 (0.85 - 0.98)
Mixed	1.82 (1.36 - 2.47)	0.92 (0.86 - 0.98)	1.36 (1.01 - 1.85)
Other	2.12 (1.56 - 2.96)	1.50 (1.08 - 2.08)	1.45 (1.04 - 2.03)
**Socioeconomic status**			
IMD quintile 1 (most deprived)	1.51 (1.41 - 1.62)	1.18 (1.09 - 1.27)	1.20 (1.09 - 1.31)
IMD quintile 2	1.34 (1.25 - 1.44)	1.13 (1.05 - 1.22)	1.19 (1.10 - 1.29)
IMD quintile 3	1.13 (1.06 - 1.20)	1.04 (0.97 - 1.11)	1.12 (1.04 - 1.20)
IMD quintile 4	1.06 (0.99 - 1.12)	1.02 (0.95 - 1.09)	1.05 (0.98 - 1.12)
IMD quintile 5 (least)		1.00 (reference)	
None	1.18 (0.73 - 2.02)	1.24 (0.72 - 2.13)	1.06 (0.61 - 1.83)

	SU		
	Univariate models	Fully adjusted model*	Fully adjusted mixed effects model**
**Gender**			
Female		1.00 (reference)	
Male	1.21 (1.16 - 1.25)	1.24 (1.19 - 1.29)	1.21 (1.17 - 1.25)
**Ethnicity**			
White		1.00 (reference)	
Asian	1.48 (1.39 - 1.58)	1.29 (1.20 - 1.39)	1.29 (1.19 - 1.39)
Black	1.43 (1.31 - 1.56)	1.31 (1.18 - 1.44)	1.18 (1.07 - 1.31)
Missing	0.94 (0.89 - 0.99)	0.93 (0.88 - 0.98)	0.94 (0.88 - 1.00)
Mixed	1.34 (1.09 - 1.64)	1.38 (1.11 - 1.71)	1.21 (0.99 - 1.49)
Other	1.45 (1.18 - 1.78)	1.30 (1.04 - 1.62)	1.27 (1.02 - 1.58)
**Socioeconomic status**			
IMD quintile 1 (most deprived)	1.21 (1.15 - 1.28)	1.04 (0.97 - 1.10)	1.12 (1.05 - 1.21)
IMD quintile 2	1.18 (1.11 - 1.25)	1.06 (0.99 - 1.12)	1.10 (1.03 - 1.18)
IMD quintile 3	1.08 (1.02 - 1.14)	1.03 (0.97 - 1.09)	1.08 (1.02 - 1.15)
IMD quintile 4	1.00 (0.95 - 1.06)	0.98 (0.92 - 1.04)	0.99 (0.94 - 1.05)
IMD quintile 5 (least)		1.00 (reference)	
None	0.91 (0.59 - 1.38)	0.93 (0.59 - 1.45)	1.00 (0.64 - 1.57)

	DPP-4		
	Univariate models	Fully adjusted model*	Fully adjusted mixed effects model**
**Gender**			
Female		1.00 (reference)	
Male	1.09 (1.05 - 1.14)	1.08 (1.03-1.13)	1.08 (1.03 - 1.12)
**Ethnicity**			
White		1.00 (reference)	
Asian	1.46 (1.37 - 1.56)	1.29 (1.19-1.39)	1.10 (1.01 - 1.20)
Black	1.17 (1.05 - 1.29)	0.99 (0.89-1.11)	0.97 (0.86 - 1.09)
Missing	1.00 (0.94 - 1.06)	0.98 (0.92-1.04)	0.94 (0.87 - 1.01)
Mixed	1.11 (0.88 - 1.39)	1.08 (0.85-1.38)	0.92 (0.73 - 1.17)
Other	1.70 (1.37 - 2.10)	1.51 (1.20-1.89)	1.17 (0.93 - 1.47)
**Socioeconomic status**			
IMD quintile 1 (most deprived)	1.33 (1.25 - 1.42)	1.13 (1.05-1.21)	1.17 (1.07 - 1.28)
IMD quintile 2	1.26 (1.18 - 1.34)	1.12 (1.05-1.20)	1.11 (1.02 - 1.20)
IMD quintile 3	1.21 (1.13 - 1.28)	1.14 (1.07-1.22)	1.06 (0.99 - 1.14)
IMD quintile 4	1.12 (1.05 - 1.19)	1.10 (1.03-1.17)	1.05 (0.98 - 1.13)
IMD quintile 5 (least)		1.00 (reference)	
None	1.09 (0.66 - 1.72)	1.16 (0.70-1.90)	0.94 (0.56 - 1.58)

	GLP-1		
	Univariate models	Fully adjusted model*	Fully adjusted mixed effects model**
**Gender**			
Female		1.00 (reference)	
Male	0.91 (0.85 - 0.98)	1.02 (0.94 - 1.11)	0.82 (0.77 - 0.88)
**Ethnicity**			
White		1.00 (reference)	
Asian	0.53 (0.45 - 0.61)	0.55 (0.46 - 0.65)	0.37 (0.31 - 0.44)
Black	0.61 (0.49 - 0.74)	0.45 (0.36 - 0.57)	0.45 (0.35 - 0.57)
Missing	0.83 (0.75 - 0.92)	0.85 (0.76 - 0.96)	0.82 (0.73 - 0.93)
Mixed	0.58 (0.35 - 0.90)	0.54 (0.33 - 0.89)	0.38 (0.23 - 0.62)
Other	0.60 (0.36 - 0.94)	0.44 (0.27 - 0.74)	0.42 (0.26 - 0.69)
**Socioeconomic status**			
IMD quintile 1 (most deprived)	1.27 (1.14 - 1.42)	0.89 (0.79 - 1.00)	1.15 (1.00 - 1.33)
IMD quintile 2	1.20 (1.07 - 1.33)	0.88 (0.78 - 1.00)	1.09 (0.96 - 1.25)
IMD quintile 3	1.25 (1.13 - 1.40)	1.00 (0.89 - 1.12)	1.14 (1.01 - 1.29)
IMD quintile 4	1.17 (1.05 - 1.30)	1.02 (0.91 - 1.14)	1.13 (1.01 - 1.27)
IMD quintile 5 (least)		1.00 (reference)	
None	0.50 (0.12 - 1.34)	0.33 (0.10 - 1.10)	0.48 (0.15 - 1.58)

	SGLT2		
	Univariate models	Fully adjusted model*	Fully adjusted mixed effects model**
**Gender**			
Female		1.00 (reference)	
Male	1.09 (1.02 - 1.17)	1.03 (0.95 - 1.11)	1.00 (0.93 - 1.08)
**Ethnicity**			
White		1.00 (reference)	
Asian	1.20 (1.08 - 1.34)	0.88 (0.77 - 1.00)	0.68 (0.58 - 0.79)
Black	0.52 (0.41 - 0.64)	0.33 (0.26 - 0.42)	0.50 (0.39 - 0.65)
Missing	0.90 (0.82 - 1.00)	0.87 (0.77 - 0.97)	0.90 (0.79 - 1.03)
Mixed	0.88 (0.57 - 1.30)	0.76 (0.50 - 1.17)	0.69 (0.44 - 1.06)
Other	0.99 (0.66 - 1.45)	0.62 (0.41 - 0.94)	0.71 (0.46 - 1.09)
**Socioeconomic status**			
IMD quintile 1 (most deprived)	1.45 (1.31 - 1.61)	1.07 (0.96 - 1.20)	1.09 (0.95 - 1.27)
IMD quintile 2	1.25 (1.12 - 1.39)	1.01 (0.90 - 1.13)	1.06 (0.92 - 1.21)
IMD quintile 3	1.12 (1.00 - 1.25)	0.96 (0.85 - 1.08)	0.97 (0.85 - 1.10)
IMD quintile 4	1.19 (1.07 - 1.32)	1.12 (1.00 - 1.25)	1.12 (1.00 - 1.27)
IMD quintile 5 (least)		1.00 (reference)	
None	0.87 (0.30 - 1.93)	0.71 (0.27 - 1.85)	0.73 (0.28 - 1.93)

* Adjusted for x,y,z (variables described in the manuscript/Appendix)
** Model adjusted for age, sex, ethnicity, and SES only; with patients additionally nested within primary care practices

	Glycaemic control (Latest HbA1c)		
	Univariate models	Fully adjusted model*	Fully adjusted mixed effects model**
**Gender**			
Female		1.00 (reference)	
Male	0.74 (0.45 - 1.04)	0.08 (-0.19 to 0.35)	0.31 (0.02 - 0.59)
**Ethnicity**			
White		1.00 (reference)	
Asian	3.53 (3.01 - 4.04)	0.29 (-0.20 to 0.77)	1.10 (0.49 - 1.71)
Black	5.40 (4.66 - 6.14)	2.18 (1.51 to 2.85)	2.36 (1.53 - 3.19)
Missing	0.79 (0.37 - 1.21)	0.57 (0.19 to 0.94)	0.65 (0.16 - 1.14)
Mixed	2.12 (0.44 - 3.80)	0.07 (-1.40 to 1.53)	0.04 (-1.60 - 1.67)
Other	3.13 (1.43 - 4.83)	-0.70 (-2.17 to 0.78)	0.87 (-0.83 - 2.57)
**Socioeconomic status**			
IMD quintile 1 (most deprived)	3.76 (3.31 - 4.20)	1.46 (1.06 to 1.87)	1.86 (1.29 - 2.42)
IMD quintile 2	2.60 (2.14 - 3.06)	0.71 (0.31 to 1.11)	1.53 (1.01 - 2.05)
IMD quintile 3	1.64 (1.19 - 2.09)	0.67 (0.28 to 1.06)	1.20 (0.72 - 1.68)
IMD quintile 4	0.67 (0.23 - 1.10)	0.22 (-0.16 to 0.59)	0.56 (0.11 - 1.01)
IMD quintile 5 (least)		1.00 (reference)	
None	0.08 (-3.31 - 3.48)	-0.51 (-3.46 to 2.44)	0.03 (-3.44 - 3.49)

* Adjusted for x,y,z (variables described in the manuscript/Appendix)
** Model adjusted for age, sex, ethnicity, and SES only; with patients additionally nested within primary care practices
