# Supplementary material for: Disparities in glycaemic control, monitoring, and treatment of type 2 diabetes in England: A retrospective cohort analysis
Source: PLoS Med. 2019 Oct 7;16(10):e1002942. doi: 10.1371/journal.pmed.1002942 (PMC6779242; doi:10.1371/journal.pmed.1002942)
Supplement: S2 Table — (DOCX) [file pmed.1002942.s003.docx]

**S2 Table.** The numbers (proportion) of people with annual data for 0, 1, 2, 3, 4 or 5 years; for each monitoring variable

| **No. of years** | **HbA1c**  **n (%)** | **BP**  **n (%)** | **eGFR**  **n (%)** | **Retinopathy**  **n (%)** | **Neuropathy**  **n (%)** |
| --- | --- | --- | --- | --- | --- |
| 0 | 340 (0.7) | 184 (0.4) | 252 (0.5) | 3,795 (7.7) | 2,299 (4.7) |
| 1 | 1,331 (2.7) | 1,049 (2.1) | 1,277 (2.6) | 5,089 (10.3) | 4,636 (9.4) |
| 2 | 2,251 (4.6) | 2,009 (4.1) | 2,132 (4.3) | 6,225 (12.6) | 5,622 (11.4) |
| 3 | 3,520 (7.1) | 2,942 (6.0) | 3,632 (7.4) | 8,551 (17.3) | 8,848 (17.9) |
| 4 | 7,880 (16.0) | 7,908 (16.0) | 9,453 (19.1) | 12,934 (26.2) | 14,253 (28.9) |
| 5 | 34,058 (69.0) | 35,288 (71.5) | 32,634 (66.1) | 12,786 (25.9) | 13,722 (27.8) |
