# Supplementary material for: Disparities in glycaemic control, monitoring, and treatment of type 2 diabetes in England: A retrospective cohort analysis
Source: PLoS Med. 2019 Oct 7;16(10):e1002942. doi: 10.1371/journal.pmed.1002942 (PMC6779242; doi:10.1371/journal.pmed.1002942)
Supplement: S1 Text — (DOCX) [file pmed.1002942.s004.docx]

**Surrey Real World Evidence Centre – Project 2: Further Insights into the management of type 2 diabetes**

(24/08/2016 Version 1.8)

**University of Surrey team:**

**Simon de Lusignan** ^1^

Professor of Primary Care and Clinical Informatics

Chair in Health Care Management, University of Surrey

Director of Royal College of General Practitioners (RCGP) Research and Surveillance Centre (RSC) [s.lusignan@surrey.ac.uk](mailto:s.lusignan@surrey.ac.uk)

**Andrew McGovern ^1^**

Clinical Researcher

Section of Clinical Medicine & Ageing,

Department of Clinical & Experimental Medicine, University of Surrey

[a.mcgovern@surrey.ac.uk](mailto:a.mcgovern@surrey.ac.uk)

**William Hinton ^1^**

Research Fellow

Section of Clinical Medicine & Ageing,

Department of Clinical & Experimental Medicine, University of Surrey

[w.hinton@surrey.ac.uk](mailto:w.hinton@surrey.ac.uk)

**Neil Munro ^1^**

Visiting Professor

Primary Care Diabetes

[n.munro@surrey.ac.uk](mailto:n.munro@surrey.ac.uk)

**Martin Whyte ^1^**

Senior Lecturer & Consultant in Diabetes & Endocrinology

Section of Clinical Medicine & Ageing,

Department of Clinical & Experimental Medicine, University of Surrey

[m.b.whyte@surrey.ac.uk](mailto:m.b.whyte@surrey.ac.uk)

**Filipa Ferreira** * ^1^

Project Manager

Section of Clinical Medicine & Ageing,

Department of Clinical & Experimental Medicine, University of Surrey

[f.ferreira@surrey.ac.uk](mailto:f.ferreira@surrey.ac.uk)

**Tom Chan** ^1^

Senior Research Fellow

Section of Clinical Medicine & Ageing,

Department of Clinical & Experimental Medicine, University of Surrey

[t.chan@surrey.ac.uk](mailto:t.chan@surrey.ac.uk)

**John Williams ^1^**

Senior Clinical Research Fellow

Section of Clinical Medicine & Ageing,

Department of Clinical & Experimental Medicine, University of Surrey

[j.g.williams@surrey.ac.uk](mailto:j.g.williams@surrey.ac.uk)

**David Russell-Jones ^1^**

Professor of Diabetes

University of Surrey

[D.Russell-Jones@surrey.ac.uk](mailto:D.Russell-Jones@surrey.ac.uk)

**Eli Lilly and Company team:**

**Bradley H Curtis** ^2^

Principal Research Scientist, Global Medical Affairs

Eli Lilly and Company

[curtis_bradley_h@lilly.com](mailto:curtis_bradley_h@lilly.com)

**Joe Jansen** ^2^

Associate Consultant, Global Operations

Eli Lilly and Company

[jansen_joe@lilly.com](mailto:jansen_joe@lilly.com)

**Kate van Brunt** ^3^

Research Scientist, Diabetes

Eli Lilly and Company

[van_brunt_kate@lilly.com](mailto:van_brunt_kate@lilly.com)

**Silvio Calderara** ^4^

Consultant-Real World Evidence Diabetes (EuCAN)

Eli Lilly and Company

[calderara_silvio@lilly.com](mailto:calderara_silvio@lilly.com)

^1^ Section of Clinical Medicine & Ageing

Department of Clinical & Experimental Medicine

University of Surrey

Guildford

Surrey GU2 7XH

W: [www.clininf.eu](http://www.clininf.eu)

^2^ Eli Lilly and Company

Lilly Corporate Centre,

Indianapolis IN 46285 U.S.A.

^3^ Eli Lilly and Company

Windlesham, Surrey,

United Kingdom

^4^ Eli Lilly and Company

1214 Vernier-Geneva

Switzerland

*Author for Correspondence

| **Surrey Real World Evidence Centre**  **– Project 2: Further Insights into the management of type 2 diabetes**  **Project summary**  **1 List of abbreviations** |  |
| --- | --- |
| **2 background and RationalE** |  |
| **3 Study aims and Objectives** |  |
| **4 Plan of investigations** |  |
| **4.1 Overview of study design (i.e. observational study of a cohort)**  **4.2 Data source**  **4.3 Data extraction**  **4.4 Definition of the Cohort: inclusion and exclusion criteria**  **4.5 Sample size considerations**  **4.6 Consent issues**  **4.7 Outcome measures**  Differences in prescribing and health outcomes across socioeconomic and other groups  Targeted interventions: stratification by macrovascular complications  **4.8 Data processing**  **4.9 Analysis plan**  **5 RESEARCH AND INFORMATION GOVERNANCE**  **5.1 Research ethics considerations**  **5.2 Information governance considerations**  Data sharing Service Level Agreement (SLA)  Fair processing principle and patient opt out  Information security |  |
| **6 Project Management** |  |
| **6.1 Reporting and accountability arrangements**  Project Advisory Group  Project Operational Team  **6.2 Patient and practice involvement**  **6.3 Dissemination** |  |
|  |  |
| **APPENDIX**  **References** |  |

**Project summary**

**background:**

**There are many options for the second and third line management of Type 2 diabetes (T2DM). This includes injectable as well as oral medications. It is important that medications are prescribed based on clinical need and that there are no disparities in prescribing based on socio-economic status or to vulnerable patient groups.**

**Whilst clinical trials have demonstrated the efficacy of hypoglycaemic agents for glucose lowering and in a limited number of cases, for cardiovascular benefit, the effectiveness of these drugs in day-to-day clinical practice may be very different. Real world evidence is needed to understand comparative effectiveness, and how medications are used in practice.**

**OBJECTIVES:**

**To describe:**

**1) Disparities in the prescribing and outcomes of people with T2DM; and**

**2) Whether glycated haemoglobin (HbA1c) twelve months after diagnosis of T2DM, is independently associated with lowering the proportion of new macrovascular (MACE) events after 10 years.**

**Method:**

**We will use data held in the Royal College of General Practitioners (RCGP) Research and Surveillance Centre (RSC) database for this study; utilising our existing cohort (N >55,000) of adult patients with T2DM to compare prescribing rates of different classes of agents used to treat T2DM. We will also explore other healthcare indicators across different age, gender, ethnic, socioeconomic groups, and localities.**

**For the second part of this investigation we will stratify people according to the presence of retinopathy at T2DM diagnosis (as a surrogate for duration of hyperglycaemia prior to diagnosis of diabetes), and compare major adverse cardiac events, all-cause mortality, as well as other important outcomes between these groups, to establish the impact of early intensification in people with T2DM.**

**Anticipated outcomes:**

**The study will inform whether healthcare disparities exist in the prescribing trends for T2DM patients from different groups; and whether early intensive treatment following diagnosis will help prevent macrovascular events and improve survival.**

**1 List of abbreviations**

| DPP-4 | Dipeptidase peptidase 4 inhibitors |
| --- | --- |
| GLP-1 | Glucagon like peptide 1 |
| GP | General Practice |
| HSCIC | Health and Social Care Information Centre |
| HbA1c | Glycated haemoglobin |
| IGT | Information Governance Toolkit |
| IMD | Index of Multiple Deprivation |
| MACE | Major Adverse Cardiac Event |
| MPR | Medication Possession Ratio |
| P4P | Pay-for-Performance, in the context of this study the implementation of the Quality and Outcomes Framework (QOF). |
| PHE | Public Health England |
| QOF | Quality and Outcomes Framework, a P4P scheme for incentivised chronic disease management. It includes diabetes. |
| RCGP | Royal College of General Practitioners |
| REC | Research Ethics Committee |
| RES | Research and Enterprise Support, University of Surrey |
| RSC | Research and Surveillance Centre (part of RCGP) |
| RWE | Real World Evidence |
| SLA | Service Level Agreement |
| SGLT2 | Sodium glucose co-transporter 2 |
| T1DM | Type 1 diabetes mellitus |
| T2DM | Type 2 diabetes mellitus |

**2 background and RationalE**

***Diabetes is an important condition, with multiple therapeutic options:***

Diabetes affects a significant proportion of the population worldwide^[[1]](#endnote-1)^. In the last 10 years, many new therapeutic agents have been introduced into clinical practice for the treatment of diabetes in primary care. These new classes of therapeutic agents include dipeptidase petidase 4 (DPP-4) inhibitors, glucagon like peptide 1 (GLP-1) receptor agonists, and sodium glucose co-transporter 2 (SGLT2) inhibitors. Over the same period, there has been the introduction of an increased range of options for insulin therapy, both in terms of types of insulin as well as administration methods^[[2]](#endnote-2),^^[[3]](#endnote-3)^. Further complexity has been added by changes to targets set in guidelines, and standardization of glycated haemoglobin assay methods (HbA1c)^[[4]](#endnote-4)^.

***Real world evidence (RWE) is needed alongside trial evidence:***

Real world evidence (RWE) is needed alongside trial evidence. Trial evidence will always be required in order to assess and to compare efficacy and safety; however, RWE provides insights into effectiveness in clinical practice, where factors such as medication adherence are so important^[[5]](#endnote-5)^. As the number of therapeutic options expands in diabetes, there is an increasing need to compare different combinations of therapeutic options that might be chosen^[[6]](#endnote-6)^.

There is growing emphasis of collecting real-world data on patients with diabetes. For example, Merck® recently announced the initiation of a global registry of type 2 diabetes mellitus (T2DM) patients, aimed at capturing data to evaluate blood glucose control, healthcare resource utilization, medication adherence, quality of life and patient satisfaction^[[7]](#endnote-7)^. Such platforms allow for the examination of key questions regarding various aspects of diabetes management, including:

- How is disease progression affected by available therapies?
- What are significant predictors of poor outcomes?
- What is the safety profile of a specific therapy?
- How does clinical practice vary, and what factors predict choice of glucose-lowering agent?
- Are there disparities in the delivery and/or outcomes of care?
- What characteristics (patient, practitioner, agent) or practices enhance adherence?

***University of Surrey-Lilly Real world evidence (RWE) centre:***

Eli-Lilly has funded the establishment of a RWE centre for the study of diabetes at the University of Surrey^[[8]](#endnote-8)^. The collaboration is now in its second year of a five year agreement. Part of this research collaboration includes further development of expertise in the processing and interpreting of clinical data for people with diabetes. This will involve the use, subject to ethical approval, of the Clinical Informatics and Health Outcomes Research Group’s^[[9]](#endnote-9)^ data assets, both current and future. Of particular interest are healthcare disparities in the care of people with type 2 diabetes and the association of early and effective glycaemic control within the first 12 months of T2DM diagnosis on long-term macrovascular events.

The goal of this RWE centre is to explore how T2DM is managed and, with respect to this study, how medicines are used in clinical practice. This type of research is needed because the clinical environment differs from the trial environment, within which adherence to therapy is mandated and strict inclusion and exclusion criteria are applied. Effectiveness in the real clinical setting may be different from that in clinical trials, which are conducted to demonstrate efficacy and safety.

***Scope of the data used – Primary care & Prescription data***

The data sources for these studies are: primary care data, anonymised coded data from general practice computerised medical record (CMR) systems and prescription data. The research group have considerable experience of using routinely collected primary care data^[[10]](#endnote-10)^. Our goal is to explore whether prescribing disparities exist in the T2DM population across different groups including socioeconomic groups.

The primary source of our primary care data will be the Royal College of General Practitioners (RCGP) Research and Surveillance Centre (RSC) practices. This is a long established group of experienced data collecting general practices. Many have been involved with the RCGP RSC for many decades, which was founded in 1957. Collected data from the group are widely regarded as being of the highest quality. In the last decade a further stimulus that has improved data quality has been the UK primary care pay-for-performance system for chronic disease management, the Quality and Outcomes Framework. This has improved data quality and promoted more active management of diabetes in primary care^[[11]](#endnote-11),^^[[12]](#endnote-12)^.

Over the next five years of this developmental research programme, we plan to utilise a wider range of data sources including Hospital Episode Statistics (HES)^[[13]](#endnote-13)^. However, further data linkage is not part of this particular study.

***Healthcare disparities in the care of people with type 2 diabetes***

It has previously been suggested that glycaemic control is worse in people with lower socioeconomic status^[[14]](#endnote-14)^. The reasons for this are complex and likely to include increased prevalence of obesity and other co-morbidities, reduced access to healthcare, and issues relating to medication compliance and self-management^[[15]](#endnote-15)^. Our early work using the University of Surrey-Lilly Real World Evidence (RWE) dataset suggests that disparities may also exist in the prescribing of diabetes therapies across different socio-economic groups^[[16]](#endnote-16)^^[[17]](#endnote-17)^. Specifically we have found that crude prescribing rates of the newer therapies: dipeptidyl peptidase-4 (DPP-4) inhibitors and the sodium glucose co-transporter 2 (SGLT2) inhibitors were lower in people with T2DM who were more deprived. If diabetes control is worse amongst people from more deprived areas, we might expect prescribing rates to be higher amongst this group of patients. Further investigation into these potential disparities is merited.

***Targeted interventions: stratification by macrovascular complications***

The United Kingdom Prospective Diabetes Study (UKPDS) showed the benefits of early intensive glucose control in newly diagnosed T2DM, following a ten-year follow-up that suggested a “legacy” effect. The drive to achieve near normoglycaemia in most (if not all) patients was reconsidered in light of the ADVANCE and ACCORD studies. The ADVANCE and ACCORD trials enrolled high risk patients, 8 and 12 years older, than those in the UKPDS. These patients had been treated for 8 and 10 years, whereas UKPDS were newly diagnosed and treatment naïve. Around 8% of patients in the UKPDS had a history of macrovascular disease, compared with about a third in the ADVANCE and ACCORD studies.

The ADVANCE study (using sulphonylureas) showed an increased risk of severe hypoglycaemia and more hospitalisations with intensive glucose control^[[18]](#endnote-18)^, whilst the ACCORD study (designed to achieve an HbA1c <6%) did not significantly lower the number of major cardiovascular events compared with targeting levels of 7.0–7.9%.^[[19]](#endnote-19)^ In fact, ACCORD was stopped early because of increased death rates in the lower target group. There is still considerable controversy as to the cause of the excess deaths seen in this group including hypoglycaemia and polypharmacy. Not only was glucose reduction achieved rapidly in the trial but many classes of glucose lowering agents were sometimes needed to achieve the lower glycaemic targets. This has led to some relaxation of glycaemic targets in patients with long standing diabetes associated with cardiovascular complications.

We do not know which therapies are most likely to achieve normoglycaemia. Early, good glycaemic control may be important not just for beta-cell preservation but also in relation to prevention of macrovascular events and improving survival. A study of almost 3,800 patients within the UK showed that the HbA1c 3-6 months after diagnosis was an independent predictor of all-cause mortality (up to 9 years later) after adjustment for age, smoking history, gender and BP^[[20]](#endnote-20)^.

In comparison to changing guidelines for the management of hypertension or cholesterol, the procedures for managing hyperglycaemia have seen relatively little change in recent years in spite of improvements in the detection of hyperglycaemia earlier in disease continuum of T2DM. These generally focus on the traditional stepwise dietary and lifestyle change – usually for up to 3-months, followed by the gradual introduction and escalation of oral and (later) injectable therapy^[[21]](#endnote-21)^.

More recent data suggests that ‘aggressive’ glycaemic control, immediately after the point of diagnosis, leads to a reduction in macrovascular events (although not in survival)^[[22]](#endnote-22)^,^[[23]](#endnote-23)^. These data also highlight the need for adequate duration of follow-up, in order to determine an effect on major adverse cardiac events (MACE). It may be the case that glycaemic targets need to be tailored according to the duration of hyperglycaemia and/or the presence of macrovascular disease at the point of diagnosis (ACCORD, ADVANCE).

**3 Study aims and Objectives**

The proposed study is part of a developing programme of related research projects in Diabetes: the *FURTHER INSIGHTS PROJECTS*. The study will utilise a real world longitudinal data platform for people with diabetes addressing information technology, governance, and operational issues in working with GP practices. The study described in this protocol has a focused aim and limited objectives, exploring healthcare disparities in the care of people with T2DM and the association of early and effective glycaemic control within the first 12 months of T2DM diagnosis on long-term macrovascular events.

The present study will utilise an existing cohort of patients with T2DM (> 55,000), spanning over a period of 10 years between 01/04/2005 and 31/01/2016, using retrospectively collected data as part of routine clinical practice. There will be no new or additional data collected.

The study will extract data to answer the following research questions:

1. Are there healthcare disparities (inequalities across age and gender, socioeconomic groups, localities, and difference ethnic groups) in the care and subsequent outcomes of people with type 2 diabetes?

The ultimate aim is to improve management of patients where care disparities exist and should subsequently lead to reduced disease burden.

1. Is HbA1c, twelve months after diagnosis of T2DM, independently associated with the incidence of macrovascular (MACE) events after 10 years?

Data from this study should help to inform the early management of people with type 2 diabetes from first presentation and diagnosis. The ultimate aim is to reduce macrovascular complications in people with type 2 diabetes.

**4 Plan of investigations**

**4.1 Overview of study design**

***Database***

The proposed study will utilise the existing pseudonymised surveillance data of the Royal College of General Practitioners, Research and Surveillance Centre (RCGP RSC) held in the secure server of the Clinical Informatics Group, University of Surrey.

***Study Population***

Individuals included in this study will be a cohort of people with T2DM identified using algorithms developed by the Clinical Informatics & Health Outcomes Research Group that combines the appropriate diagnostic and treatment criteria^[[24]](#endnote-24),^^[[25]](#endnote-25),^^[[26]](#endnote-26)^. The study will include the relevant retrospective clinical data for the cohort for a ‘before’ (baseline) period; and then for a ten year observation period from 1^st^ April 2005 to 31^st^ January 2016. The large population of the database will mean that the proposed study should generate useful information about the management of patients with T2DM in primary care, and contribute to improving the quality of care for people with T2DM.

People will be classified as having T2DM or not on 1^st^ April 2005 based on data held up to that point. Pay-for-performance (P4P) for chronic disease management had its preparatory year in 2003 and was implemented in 2004. This should ensure that the study can identify cases.

***Exposure:***

The study intends to analyse two areas:

1. Comparison of the quality of diabetes care amongst people with different age, gender, socioeconomic, and ethnic backgrounds in the UK, and exploring whether disparities in prescribing rates, and other healthcare indicators exist across these groups.
2. Explore whether there is an association between early and effective glycaemic control within the first 12-months after diagnosis of T2DM, stratified by presence or absence of retinopathy at diagnosis, on long-term macrovascular event rates in the UK.

***Outcome measures:***

*Healthcare disparities*

Our outcome measures will be glycaemic control stratified by demographic factors (socioeconomic status, age, ethnicity, etc.), prescribing rates across different therapies (oral and injectable), and other important measures for diabetes processes of care.

*Targeted Interventions*

Outcomes measures will include MACE, all-cause mortality, and the impact of early intensification on other important outcomes in people with diabetes.

**4.2 Data source**

The proposed study is a secondary analysis of the existing and pseudonymised clinical coded surveillance data of the RCGP RSC network of GP practices. This is held in the secure server of the Clinical Informatics Group, University of Surrey. The study will use the retrospective observational dataset as it is updated on 31^st^ January 2016, or other appropriate end of quarter date.

The RCGP RSC database is a nationally representative sample, drawn from all health regions across England. The RCGP RSC is the gold standard surveillance network. It collects data each week from over 1,300,000 patients distributed across England to provide national surveillance data^[[27]](#endnote-27)^. This national surveillance is provided in collaboration with Public Health England (PHE). The RCGP RSC was established in 1957, and has a long history of engagement in research and surveillance.

The Clinical Informatics & Health Outcomes Research Group is working in partnership with the RCGP’s RSC through a formal service agreement. The data, information technology assets, analysis capability, and leadership of the RCGP RSC are based at Clinical Informatics & Health Outcomes Research Group, University of Surrey. The Clinical Informatics & Health Outcomes Research Group and the RCGP are jointly involved in a range of research and surveillance programmes. The network runs in close collaboration with Public Health England (PHE) who provide a reference laboratory that carefully reports microbiological specimens collected in practices.

The medical record for the RCGP RSC is sourced from GP computerised medical record (CMR) systems. These CMR systems broadly adopt 2 coding schemes (Read 2 and CTV3)^[[28]](#endnote-28)^, but slightly different interfaces and preferred terms in the look-up tables. This may produce slightly different levels of recording of codes, particularly non-QOF codes (Quality and Outcomes Framework)^6,^^[[29]](#endnote-29)^.

The Clinical Informatics & Health Outcomes Research Group, as the data custodian and analysis hub, has direct access to RCGP RSC dataset. Data are uploaded from the network weekly, to a secure sever, with the option to switch to twice weekly uploads at time of epidemics. This currently contains all coded clinical data for patients registered in over 140 practices within the RCGP RSC network of GP practices, going back to 2004. The number of GP practices participating in the network is increasing, with the number of practices expected to increase to 200 in the next few months. The registered population of these practices will be in excess of 1,500,000 patients.

**4.3 Data extraction**

We plan to use Apollo Medical Software Solutions to provide the data extraction and network transferring services^[[30]](#endnote-30)^. Apollo Medical Software Solutions is a third party commercial company that has a formal data consent agreement with the RCGP RSC and GP practices within the RCGP RSC network, for data extraction and data transfer. Within this data consent agreement, Apollo Medical Software Solutions provides assurance on information security and protection of confidentiality.

Apollo Medical Software Solutions have considerable experience of GP extraction, and the Research Group has developed with them a customised bulk plus incremental extraction specification that will meet the needs of this project. The following routinely collected clinical coded data will be extracted for the study (see Appendix 1 for more details):

- Demographic information: age, gender, ethnicity, registered date.
- Postcode: Full postcodes will be immediately transformed into deprivation scores using GIS methods (Geographical Information System) within GP computer systems upon extraction. Only the first part of the postcodes will be retained for identification of general locality.
- Diabetes specific information – diagnosis and diabetes-related complication information.
- Diabetes therapy/medication information, and medication for cardiovascular comorbidities.
- General health and health behaviour information – e.g. Lifestyle/risk factors, BMI (Body Mass Index), smoking status, etc.
- Comorbidities – records of other diseases and long term conditions, e.g. chronic respiratory disease, chronic heart disease, chronic kidney disease, chronic liver disease, chronic neurological disease, immunosuppression, pneumonia, etc. There will be a particular focus on cardiovascular comorbidities, and other diabetes complications such as retinal disease, and peripheral neuropathy.
- Concomitant medication.
- Referral to further care.

**4.4 Definition of the Cohort: inclusion and exclusion criteria**

Practice level

- All GP practices within the RCGP RSC network of practices will be included in the study

Patient level

- All adult registered patients of the RCGP RSC network of practices meeting the criteria for T2DM (based on the algorithms developed by the Research Group).
- The existing diabetes algorithm will be refined to exclude people with less common diabetes types from the cohort including monogenic diabetes (previously known as maturity diabetes of the young (MODY)), pancreatic diabetes; type 3c diabetes, and secondary diabetes. These rarer type of diabetes are poorly coded in routine data and we will validate methods to identify them using other indicators (for example, the presence of preceding pancreatic disease in the case of pancreatic diabetes).

Existing work on the RCGP RSC database has shown the following regarding data completeness:

- There is a cohort of 58,717 adult patients with T2DM and 956,970 adult patients without T2DM with complete data for a period of at least 10 years (April 2005 - January 2016).
- For age, gender, and GP practice, all people with T2DM have 100% data completion, and for deprivation score (derived from postcode), this is 96%.
- For smoking status, blood pressure, and BMI, the T2DM cohort has data completion of at least 98% across each variable (99%, 99%, and 98%). This is 48% in the non-T2DM cohort.
- 98% of patients with T2DM have an HbA1c measurement (57,502 patients).
- 57% of the T2DM cohort has hypertension (33,467 patients).

**4.5 Sample size consideration**

The present study will follow the health experiences and health outcomes of a cohort of patients with T2DM over a ten year period between 01/04/2005 and 31/01/2016 using retrospective data, collected routinely as part of clinical practice in the RCGP RSC network of GP practices.

As the present study is an exploratory study a power calculation was not performed. However, the findings may inform power calculations for more definitive clinical trials.

**4.6 Consent issues**

Only existing pseudonymised data held within the secure server of the Clinical Informatics & Health Outcomes Research Group will be used for secondary analysis in this study. The objectives of this study is congruent with the purpose for which data is collected, under a data sharing agreement with the GP practices participating in the RCGP RSC network. No additional consent from GP practices within the RCGP RSC network will be sought. However, project information for this study will be shared with GP practices in the network via existing network communication mechanisms (e.g. RCGP RSC newsletters and website), prior to the start of this study.

Individual patient consent will not be sought. This will be discussed and justified in more detail in the Research Ethics and Information Governance considerations sections (Section 5.1 and 5.2).

**4.7 Outcome measures**

*Health care disparities*

The primary outcome measure will be glycaemic control in all included people with T2DM stratified by demographic factors (socioeconomic status, age, ethnicity, etc.). Measures of glycaemic control will comprise; most recent HbA1c, and time weighted HbA1c during an extended follow-up period (duration will depend on data availability).

The secondary outcome measures will include, prescribing rates of newer therapies (DPP-4 inhibitors, SGLT2 inhibitors, and GLP-1 analogues) defined as number of prescriptions per year per 1,000 people, prescribing rates of older oral and injectable therapies, and other important measures of process of care of diabetes (level of primary care quality and outcome [QOF] targets achieved; in particular annual retinal screening, glycaemic control monitoring, and renal function monitoring). Prescribing rates will be calculated using prescription data recorded in the primary care record. We will describe the annual crude prescribing rates stratified by each possible predictor variable and adjusted rates (adjusted for potential confounding variables). We will look at each of these measures annually over the most recent five year period.

We will use regression analysis (Section 4.9) to determine if socioeconomic status, demographic, or ethnic differences (areas of potential disparity) are independently associated with glycaemic control allowing for the following independent variables:

- Patient age
- Patient gender
- Patient ethnicity (defined using our verified ethnicity identification algorithm)
- Smoking status and alcohol use
- Duration of diabetes
- Diabetes complications
- Adherence and persistence with medications
- Comorbidities:
  - Renal impairment
  - Ischaemic heart disease
  - Previous stroke
  - Atrial fibrillation
  - Memory loss (including diagnosed dementias)
  - Chronic liver disease
  - Infections
  - Lung disease
  - Other chronic inflammatory conditions
  - Psychiatric illness (including depression)
- Other parameters:
  - Blood pressure
  - BMI
  - Missing data

We will explore these predictor variables for any important correlations which may have an impact on our conclusions. For example, we will explore if any of the major comorbidities listed have any impact on medication adherence which may subsequently impact on glycaemic control.

People will have socioeconomic status categorised by quintile using the index of multiple deprivation. This is a nationally regarded measure of deprivation which can be calculated from patients’ postcodes. This is an overall measure of deprivation for neighbourhoods, calculated using the weights of seven domains (see Appendix 2 for further details). Patient postcodes are converted to Lower Super Output Areas (LSOA), and these will be assigned to an IMD for each patient. This IMD will subsequently be divided into quintiles based on the national distribution of IMD scores.

Missing data for included parameters is likely to be an important predictor of poor outcomes as it may correlate to low propensity to consult or engage with primary care services. For example, missing smoking data is more likely to be present in people with fewer primary care contacts and therefore may be a predictor of poor outcomes in people with T2DM. Several approaches will be used to explore and adjust for this effect:

1. A subgroup analysis of only people with complete data for all the variables of interest for each analysis will be performed.
2. Imputation of missing data and the impact on models will be performed.
3. A descriptive analysis of the correlations of outcomes with missing data for each variable of interest.
4. Inclusion of a missing data category for each variable of interest into regression models to allow adjustment for missing data.

*Targeted interventions: stratification by macrovascular complications*

We will explore whether HbA1c, 12 months after diagnosis of T2DM is independently associated with lowering the proportion new macrovascular events (MACE) after 10 years.

Definition of MACE events

We will stratify people according to the presence of retinopathy at the point of diagnosis of diabetes.

- fatal or non-fatal MI, ACS hospitalisation, coronary intervention/CABG, fatal or non-fatal stroke, heart failure, amputation or urgent revascularisations.

A record of the presence of diabetic retinopathy (none, background retinopathy or maculopathy/proliferative retinopathy) at the time of diagnosis will be used as a surrogate marker of (hitherto undetected) diabetes duration and compared between groups at baseline and incorporated into the multivariate analysis.

The duration of diabetes is probably the strongest predictor for development and progression of retinopathy. Among younger-onset patients with diabetes in the Wisconsin epidemiologic study of diabetic retinopathy (WESDR), the prevalence of any retinopathy was 8% at 3 years, 25% at 5 years, 60% at 10 years, and 80% at 15 years.^[[31]](#endnote-31)^ In addition, the Australian Diabetes, Obesity and Lifestyle Study (AusDiab) showed that the prevalence of diabetic retinopathy is less than 10% in those with diabetes duration of less than 5 years but more than 50% in those with 20 years or longer diabetes^[[32]](#endnote-32)^.

Follow-up events

- MACE events: fatal or non-fatal MI, ACS hospitalisation, coronary intervention/CABG, fatal or non-fatal stroke, heart failure, amputation or urgent revascularisations

- All-cause mortality

Missing variables

Patients without an HbA1c recorded 6 months before or 3 months after diagnosis will be analysed separately. For each additional variable with missing values the approach to missing data outlined in the section above will be used. Censoring will be defined as loss-to follow-up.

**4.8 Data processing**

This data will be extracted, stored, and processed by the Clinical Informatics & Health Outcomes Research Group at the University of Surrey, and only aggregated tables will be made available in publications or to third parties.

Data extractions will be conducted in accordance with the research team’s standard operating procedures in data extraction pseudonymisation and transfer. All data processing and analysis in the present proposed study will be conducted within the secure IT environment of the Clinical Informatics & Health Outcomes Research Group, University of Surrey. The information security policies and procedures of the Research Group have been approved by the NHS Health and Social Care Information Centre (HSCIC). Details of the departmental information governance policies and procedures can be found in: <http://www.clininf.eu/about/information-governance.html>.

**4.9 Analysis plan**

R Studio within the secure analysis server is the statistical and analytical tool of choice for the Research Group^[[33]](#endnote-33)^ . Our secure analysis servers are optimised for routine healthcare data processing, to provide faster deliveries for our projects. We will look to conduct the following pieces of analysis within this study:

## *Summary statistics*

Summary statistics will be used to describe the population of interest. Means and standard deviations will be used to summarize the continuous variables; frequencies and percentages will be used to summarize the categorical variables.

## *Inferential statistics*

## Pearson’s chi-squared tests, t-tests and analysis of variance (ANOVA) will be used to compare all means and proportions respectively across the age, gender, socioeconomic, and ethnic groups, and among subgroups according to glycaemic control, to highlight any potentially substantial differences.

## Linear mixed models will be used for the analyses of impact of socioeconomic, demographic, and ethnicity factors on healthcare outcomes. The cluster effect of GP practice will also be considered. Model selection will be performed for parameter estimation.

The analysis will include innovative regression method modelling. For an outcome in diabetes management such as HbA1c on an individual patient basis, regression of HbA1c will be applied to each individual patient. This approach has a number of advantages compared to the conventional approach which measures the HbA1c in two time points^[[34]](#endnote-34)^.

Further details of the analysis to be performed for each research question are outline below.

Healthcare disparities

## Regression analyses will be used to determine the relationship between glycaemic control and age, gender, socioeconomic, and ethnic groups, adjusting for potential confounding variables. We will perform linear regression with most recent measurement of HbA1c as the outcome variable.

## We will also perform a sensitivity analysis of the regression model by repeating the analysis but with stratification of HbA1c by good (e.g. <7% / 53mmol/mol), moderate (e.g. 7-8.5%/ 53- 69mmol/mol) and poor (e.g. >8.5%/ >69mmol/mol) glycaemic control. The regression model will use this categorical measure of HbA1c as the outcome and we will therefore use ordinal logistic regression.

In addition, regression analyses will be used to determine the relationship between age, gender, socioeconomic, and ethnic groups and prescribing rates after adjusting for potential confounding variables. We will perform logistic regression with the binary outcome variable; prescribed the medication class of interest or not during the follow-up period.

As a sensitivity analysis we will perform regression using the prescription count per person over the follow up period. For this, initially the distribution of number of prescriptions per person for the medication class of interest will be analysed. Depending of the shape of this distribution we will either construct Poisson regression or negative binomial regression models as appropriate. Given the large number of people who have not yet been prescribed any drugs in the classes of interest these models will have to account for the large number of people with zero as the value for the prescription count. It is therefore likely that a zero inflated Poisson or negative binomial regression (if there is over dispersion) analysis will be the most suitable to analyse these data.

We will report both crude prescribing rates and adjusted rates (as incident risk ratios or odds ratios depending on the model chosen) with 95% confidence intervals for these models and p values for each model parameter.

We will explore the impact of model reduction using backwards stepwise elimination of variables which are not significant (p>0.05).

Targeted interventions: stratification by macrovascular complications

Univariable and multivariable Cox proportional hazard modelling will be used to analyse the association between HbA1c values and event-free survival. Event-free survival curves will be constructed using the Kaplan-Meier method and compared using log-rank test.

Logistic regression will be used to test the primary endpoint. Outcomes with HbA1c values analysed as a categorical variable according to the groups described above – with comparisons made to the reference group. HbA1c will also be modelled as a continuous variable, assuming both a linear relationship and a non-linear relationship using log-transformed HbA1c. Unadjusted and multivariate adjusted hazard ratios will be calculated.

For the multivariable modelling, covariates previously demonstrated to be independently associated with cardiovascular mortality will be included in the model (age, sex, and previous MI, hypertension, peripheral vascular disease, congestive heart failure, and PTCA/PCI, age, smoking status, body-mass index and systolic blood pressure, total cholesterol, LDL cholesterol, HDL-cholesterol, antiplatelet drug use, statins, beta-blocker, ACEi,). HbA1c at diagnosis will *a priori* be included in the model. In addition, the final multivariable model will include covariates with strong univariable associations.

Results will be reported as HR with associated 95% CI and P-values. P value <0.05 will be considered statistically significant.

**5 RESEARCH AND INFORMATION GOVERNANCE**

This study is conducted within the University of Surrey’s formal frameworks for information and research governance. In addition, all externally funded projects and collaborative projects with external partners are supported and guided by the University’s Research and Enterprise Support (RES) service. RES ensures that university supported projects are financially viable, and that legal issues of knowledge transfer and intellectual properties are addressed.

**5.1 Research ethics considerations**

Working with practitioners and a pharmaceutical company, the primary purpose of this study is to follow a cohort of patients with T2DM to explore whether glycaemic control and prescribing patterns differ across people with different socioeconomic backgrounds, and investigate the association of early and effective glycaemic control within the first 12-months after diagnosis of T2DM, on long-term macrovascular event rates. This will help us to identify areas of quality improvement initiatives. The proposed study will use pseudonymised RCGP RSC clinical data extracted from GP information systems; the data is routinely collected as part of clinical consultations in primary care.

The principal ethical issue is concerned with the protection and use of pseudonymised patient level information for the purpose of medical research without individual level patient consent. The protection and use of pseudonymised patient level information is addressed more fully in the next section: information governance considerations.

The research team will make a formal application for approval from a NHS Research Ethics Committee (REC) prior to the commencement of the study. In addition, the research team will enquire if this study requires specific approval from the Health Research Authority, Confidential Advisory Group (CAG) for Section 251 of NHS Act 2006 and Health Service (Control of Patient Information) Regulations 2002 exemption to support the use of patient level information without individual level patient consent (<http://www.hra.nhs.uk/about-the-hra/our-committees/section-251/what-is-section-251/>).

**5.2 Information governance considerations**

The Clinical Informatics & Health Outcomes Research Group at the University of Surrey has worked with routinely collected healthcare data in a number of research and evaluation projects over the last 15 years. The Research Group works within the research and Information Governance frameworks for health and social care in the United Kingdom. The University of Surrey is registered with the Information Commissioner’s Office Data Protection Register, and is compliant with the Data Protection Act, and other legislations.

Data sharing Service Level Agreement (SLA)

Existing pseudonymised coded data from the RCGP RSC network of GP practices provide the data source for this project. Formal data sharing agreements are made between GP practices participating in the RCGP RSC network and the RCGP RSC. The SLA specifies the scope of the network, how data are extracted and processed, and the respective responsibilities of the RCGP RSC and the participating GP practices. In addition, the RCGP has a formal process to approve requests from bona fide research organisations, including the Clinical Informatics & Health Outcomes Research Group at the University of Surrey, to access data on a project by project basis to support research congruent with the aim of the RCGP RSC.

Fair processing principle and patient opt out

No strong patient identifiers (NHS numbers, postcodes, dates of birth, etc.) will be extracted in this study. Additionally, no patient level data will be sent to the research sponsor/funder in a way that the individual patient can be re-identified. In line with the principle of the Data Protection Act 1998, data subjects will be informed of the uses of their data in this study. As part of the data sharing SLA, project information for research studies will be shared with GP practices of the network within existing network communication mechanisms (e.g. RCGP RSC newsletters and website) prior to the start of the projects concerned.

Participating GP practices will then be asked to display project information in their website and project information posters in reception areas to inform patients of the use of their anonymised data in this study, from when the practice has consented to take part in the study and until the study is completed. The project information will specifically refer to the right of the patients to opt out if they do not wish their data to be included in this study. The research team will respect the codes in the data indicating that a patient does not wish to have their record available for research; the research team will, however, seek to report the number of patients within a practice who have chosen to opt out to assess sample biases.

Information security

The project team is supported by IT services dedicated to the Faculty and to the Department of Clinical & Experimental Medicine.

In addition, the Research Group reviewed its departmental information governance policies and procedures, against the requirements of the NHS Information Governance Toolkit (IGT) for Hosted Secondary Use Team/Project, Version 12 (<https://www.igt.hscic.gov.uk/>). The review was approved by the Health and Social Care Information Centre, and was deemed satisfactory to support application to Confidentiality Advisory Group or the Data Access Advisory Group. The information security policies and procedures of the Research Group, approved by the NHS Health and Social Care Information Centre (HSCIC), can be accessed from: <http://www.clininf.eu/about/information-governance.html>.

1. **PROJECT MANAGEMENT**

**6.1 Reporting and accountability arrangements**

The project is accountable to the Project Steering Board, with the day-to-day operational issues managed by the Project Operational Team.

Project Steering Board

The Steering Board will meet bi-annually to receive regular and exceptional reports, including reporting of adverse events, from the Operational Team, monitor progress against set milestones, and ensure that resources and support are available to enable the successful delivery of the project within the funding agreement. In the event of a report of adverse incidents, the Project Steering Board will co-ordinate an effective management of the adverse events in line with local and national guidance, and if appropriate, onward reporting to the University, sponsor, external partners or external research and information governance authorities.

| **Steering Board Member** | **Role and Organisation** |
| --- | --- |
| Prof Simon de Lusignan | Principal Investigator, University of Surrey |
| Louise Timlin | Research Sponsor Representative |
| Prof Neil Munro | Domain Expert |
| TBC | GP/Practice representative |
| Roy Johnson | Patient Representative |
| Patrick Millar | Patient Representative |
| Dr Filipa Ferreira | Project Manager, University of Surrey |

The Project Steering Board consists of the Principal Investigator, domain experts, patient and practitioner representatives, and representative of the sponsor organisation of the study.

Project Operational Team

The operational team is responsible for the completion of the project objectives against set milestones, and submit regular and ad-hoc reports to the Project Steering Board. The Team will meet fortnightly in person and/or via teleconference, particularly in the early stages of the project, to ensure the project meets with the milestones agreed for the project.

The Operation Team consists of research staff, the project manager and the Principal Investigator of this project:

| **Team Member (TBC)** | **Lead responsibility in the project and organisation** |
| --- | --- |
| Prof Simon de Lusignan | Senior academic and clinical Lead, University of Surrey |
| Prof Neil Munro | Senior Primary Care Diabetes investigator, University of Surrey |
| Kate Van Brunt | Research Sponsor Representative, Eli Lilly |
| Dr Bradley Curtis | Principal Research Scientist, Eli Lilly |
| Silvio Calderara | Consultant-Real World Evidence Diabetes, Eli Lilly |
| Dr Filipa Ferreira | Project Manager, University of Surrey |
| William Hinton | Research Fellow, University of Surrey |
| Dr Andrew McGovern | Research Programme MD student, University of Surrey |
| Dr Martin Whyte | Senior Lecturer Nutrition & Metabolism, University of Surrey |
| Jeremy van Vlymen | Research Fellow, University of Surrey |
| Rachel Byford / Barbara Arrowsmith | Database developer, University of Surrey |
| Dr Tom Chan | Information governance lead, University of Surrey |
| Dr John Williams | Primary Care Diabetes & Primary Care Informatics, University of Surrey |
| Ana Correa | Primary Care Scientist, University of Surrey |
| Prof David Russell Jones (TBC) | Liaison with Steering Board, University of Surrey |

**6.2 Patient and practitioner involvement**

Patient involvement

Patients will be involved in the protocol development and as members of the Steering Board. The patient perspective and patient relevance are integral part of the project management, particularly in the aspects of acceptability in the use of patient information as outlined in the protocol.

Practitioner involvement

Clinical practitioners will be recruited from our existing research networks to ensure that the study is clinically relevant and operationally feasible from the practitioners’ perspective. We will look for practices purposefully to represent different social groups, brand of computerised medical record systems, and practice size (large practices may have more data extraction challenges).

**6.3 Dissemination**

A formal report with key findings, implication for practice, and call for further research will be submitted to the sponsor/funder at the end of the study.

The outputs from the research will also be disseminated through peer review papers in high impact journals within the domains of primary care and diabetes. The research team will seek to present findings at relevant local and national seminars and conferences.

**Appendix 1**

**Basic Demographics**

| Year of birth | Computed |
| --- | --- |
| Gender | Readily Available |
| Race/ethnicity | Where recorded |
| Index of multiple deprivation (IMD) | Derived from post code |
| Occupation code | Where recorded |
| Geographic region | Avoiding any potential for re-identification |

**General Clinical Information**

| Blood pressure | Readily available in record, annual recording since pay-for-performance (P4P the Quality and Outcomes Framework (QOF)) |
| --- | --- |
| BMI (also calculated from weight and height) | Readily available in record, annual recording since pay-for-performance (the Quality and Outcomes Framework) |

**General Behavioural Information**

| Diet advice | Should be part of annual assessment of diabetes in primary care, part of P4P/QOF so well recorded |
| --- | --- |
| Alcohol consumption | Should be part of annual assessment of diabetes in primary care, part of P4P/QOF so well recorded |
| Exercise advice | Should be part of annual assessment of diabetes in primary care, part of P4P/QOF so well recorded |
| Smoker / smoking history | Should be part of annual assessment of diabetes in primary care, part of P4P/QOF so well recorded |

**Diabetes-Specific Information**

| Type of diabetes (T1DM vs. T2DM) | Computed (based on Surrey algorithm), we can compare with other approaches |
| --- | --- |
| Duration of diabetes | We will look to ascertain this from pre-2005 data; generally possible to get date of diagnosis, first Px and/or blood tests |
| Family history (i.e., another HUM member with T1DM or T2DM) | Computed  We have a household key, (rarely relevant)  Family history codes are often recorded |

**Diabetes-Related Complications**

| Diabetes Complication Severity Index (DCSI) | Computed |
| --- | --- |
| ***Retinopathy*** | Computed^[[35]](#footnote-1)^ |
| ***Nephropathy*** from check of renal function and test for proteinuria and/or indication the patient is treated for renal disease (including looking for dialysis and transplant codes). | Computed |
| ***Neuropathy*** | Computed^[[36]](#footnote-2)^ |
| ***Cerebrovascular*** | Computed |
| Transient ischemic attack | Readily Available |
| Hemorrhagic stroke | Readily Available |
| Ischemic stroke | Readily Available |
| ***Cardiovascular*** | Computed |
| Other ischemic heart disease | Readily Available |
| Angina pectoris | Readily Available |
| Myocardial infarction | Readily Available |
| Ventricular fibrillation, arrest | Readily Available |
| Atrial fibrillation, arrest | Readily Available |
| Old myocardial infarction | Readily Available |
| Heart failure | Readily Available |
| Aortic aneurysm/dissection | Readily Available |
| ***Peripheral vascular disease*** | Computed |
| Diabetic peripheral vascular disease | Readily Available |
| Other aneurysm | Readily Available |
| Foot wound + complication | Readily Available |
| Claudication, intermittent | Readily Available |
| Embolism/thrombosis (lower extremity) | Readily Available |
| Gangrene | Readily Available |
| Ulcer of lower limbs | Readily Available |
| ***Metabolic*** | Computed |
| Ketoacidosis | Readily Available |
| Hyperosmolar | Readily Available |
| Other coma | Readily Available |

**Diabetes Medication Therapy**

(Grouped by British National Formula chapter heading – all prescriptions)

| **Oral Medications** | | |
| --- | --- | --- |
| Metformin | | Readily Available |
| Dose of Metformin | | Computed |
| Metformin MR | | Readily Available |
| Dose of Metformin MR | | Computed |
| Sulphonylurea | | Readily Available |
| TZDs | | Readily Available |
| Meglitinides | | Readily Available |
| AGIs | | Readily Available |
| DPP-4 inhibitors (existing and new entries as they achieve formulary) | | Readily Available |
| Januvia (sitagliptin) | | Readily Available |
| Galvus (vildagliptin) | | Readily Available |
| Onglyza (saxagliptin) | | Readily Available |
| Tradjenta (linagliptin) | | Readily Available |
| Nesina (alogliptin) | | Readily Available |
| Dose of DPP-4 inhibitors | |  |
| Januvia (sitagliptin) | | Computed |
| Galvus (vildagliptin) | | Computed |
| Onglyza (saxagliptin) | | Computed |
| Tradjenta (linagliptin) | | Computed |
| Nesina (alogliptin) | | Computed |
| SGLT2 inhibitors (existing and new entries as they achieve formulary) | |  |
| Invokana (canagliflozin) | | Readily Available |
| Farxiga (dapagliflozin) | | Readily Available |
| Jardiance (empagliflozin) | | Readily Available (2/15) |
| Dose of SGLT2 inhibitors | |  |
| Invokana (canagliflozin) | | Computed |
| Farxiga (dapagliflozin) | | Computed |
| Jardiance (empagliflozin) | | Computed |
| Oral fixed dose combinations [existing (e.g., Janumet). There are several of these for most of the above groups | |  |
| Actoplus Met XR (pioglitazone/metformin XR) | | Readily Available |
| Avandamet (rosiglitazone/metformin) | | Readily Available |
| Avandaryl (rosiglitazone/glimperide) | | Readily Available |
| Glipizide/metformin (generic, Metaglip) | | Readily Available |
| Glyburide/metformin (generic, Glucovance) | | Readily Available |
| Invokamet (canagliflozin/metformin) | | Readily Available |
| Janumet (sitagliptin/metformin) | | Readily Available |
| Janumet XR (sitagliptin/metformin XR) | | Readily Available |
| Jentadueto (linagliptin/metformin) | | Readily Available |
| Kazano (alogliptin/metformin) | | Readily Available |
| Kombiglyze XR (saxagliptin/metformin XR) | | Readily Available |
| Oseni (alogliptin/pioglitazone) | | Readily Available |
| Pioglitazone/glimepiride (generic, Duetact) | | Readily Available |
| Pioglitazone/metformin (generic, Actoplus Met) | | Readily Available |
| Prandimet (repaglinide/metformin) | | Readily Available |
| **Non-insulin Injectable medications** | | |
| GLP-1 receptor agonists - daily |  | |
| Byetta (exenatide) | Readily Available | |
| Victoza (liraglutide) | Readily Available | |
| Lyxumia (lixisenatide) | Readily Available | |
| GLP-1 receptor agonists - weekly |  | |
| Bydureon (exenatide) | Readily Available | |
| Tanzeum (albiglutide) | Readily Available | |
| Trulicity (dulaglutide) | Readily Available | |
| Dose of GLP-1 receptor agonists |  | |
| Byetta (exenatide) | Computed | |
| Victoza (liraglutide) | Computed | |
| Bydureon (exenatide) | Computed | |
| Tanzeum (albiglutide) | Computed | |
| Trulicity (dulaglutide) | Computed | |
| Injectable fixed dose combinations where one component is non-insulin [new entries as they achieve formulary status (e.g. insulin/GLP)] | Readily Available | |
| **Insulin** | | |
| Basal insulin (intermediate- and long-acting) | Readily Available | |
| Dose of basal insulin | Computed (class level) | |
| Faster-acting insulin (rapid- and short-acting) | Readily Available | |
| Dose of faster-acting insulin | Computed (class level) | |
| Bi-phasic/mixed insulin | Readily Available | |
| Dose of biphasic/mixed insulin | Computed (class level) | |
| **Other** | | |
| Glucagon | Readily Available | |

**Other Diseases of Interest** (these will be as recorded in the primary care computerised medical record; eventual linkage to hospital data (Hospital Episode Statistics-HES would improve recording of surgical events and major vascular events – principally giving more precise details of any surgery/procedure performed (e.g. angioplasty) and the precise cancer diagnosis**.**

| Charlson Comorbidity Index (CCI) | Computed |
| --- | --- |
| Liver disease - mild | Readily Available |
| Liver disease – moderate or severe | Readily Available |
| Mental Illness (excluding Alzheimer’s and other dementias)  We will separate common mental health problems (CMHP – anxiety, depression, stress) from severe and enduring psychiatric illness (schizophrenia and hypomania); memory problems and dementia; and alcoholism and addictions. | Readily Available |
| Alzheimer’s and other dementias | Readily Available |
| Gastrointestinal side effects (GI motility, abdominal pain, bloating, constipation, diarrhoea, nausea, vomiting) | Readily Available |
| Hypertension | Readily Available |
| Dyslipidaemia | Readily Available |
| Obesity | Readily Available |
| Chronic kidney disease | Readily Available |
| Chronic pulmonary disease, we will separate asthma, from COPD and other rare chronic pulmonary disease | Readily Available |
| Bariatric surgery | Readily Available |
| Amputations, ulcerations | Readily Available |
| Cancer (excluding metastatic carcinoma) | Readily Available |
| Metastatic carcinoma | Readily Available |

**Concomitant Medication Therapy (nearly all prescribing is by GPs)**

| Angiotensin-converting enzyme inhibitors | Readily Available |
| --- | --- |
| Angiotensin-II receptor antagonists | Readily Available |
| Calcium-channel blockers | Readily Available |
| Beta-blockers | Readily Available |
| Thiazides | Readily Available |
| Alpha-blockers | Readily Available |
| Fixed dose combination of antihypertensives | Readily Available |
| Centrally-acting antihypertensives | Readily Available |
| Vasodilator antihypertensives | Readily Available |
| Renin inhibitors | Readily Available |
| Statins | Readily Available |
| Fibrates | Readily Available |
| Antidepressants | Readily Available |
| Corticosteroids | Readily Available |
| Number of Prescription Drugs | Computed |
| Pill Burden | Computed |
| Whether special weekly quantities of medicines dispensed (implies a “Nomad” or other system to support adherence generally used in older people) | Readily Available |

**Laboratory Testing**

Since 2003 nearly all practices have had lab links meaning that there is longitudinal data since then for people with diabetes. Most people will have had at least an annual review

| HbA1c (most recent) | Readily Available |
| --- | --- |
| Fasting blood glucose (most recent) | Readily Available |
| LDL (most recent) | Readily Available |
| HDL (most recent) | Readily Available |
| Triglycerides (most recent) | Readily Available |
| Serum creatinine (most recent) | Readily Available |
| Dipstick for protein (most recent) | Readily Available |
| Albumin urine test (most recent) | Readily Available |

**Healthcare Resource Utilization** (only primary care data will be available for the initial INSIGHTS reports):

| Total healthcare costs | Computed^[[37]](#footnote-3)^ |
| --- | --- |
| Number of physician visits/year (rolling 12 month period) | Computed |
| Number of dialysis visits/year (rolling 12 month period) | Computed |

**Provider Information (Most Recent Visit with T2DM as Primary Diagnosis)**

| Primary care | Readily Available |
| --- | --- |
| Specialist | Available with limitations from primary care data |
| Type of specialist | Available but inconsistent. Some diabetes in general medicine, some in endocrinology, some “diabetes.” |
| Contractual arrangement (function of member) | Computed |

^All oral combination that include glucose lowering therapies will be separately analysed and reported. New glucose lowering agents will be included in the analysis as they become available for clinical use in the UK and are listed in the computerised drug dictionaries.

**Appendix 2**

A description of the index of multiple deprivation: The Index of Multiple Deprivation 2015 is the official measure of relative deprivation for small areas (Lower-layer Super Output Areas; LSOAs) in England^[[38]](#endnote-35)^. LSOAs are small areas designed to be of a similar population size, with an average of approximately 1,500 residents or 650 households. There are 32,844 LSOAs in England. The Index of Multiple Deprivation ranks every LSOA in England from 1 (most deprived area) to 32,844 (least deprived area).

The Indices of Deprivation 2015 provide a set of relative measures of deprivation for small areas (Lower-layer Super Output Areas) across England, based on seven domains of deprivation. The domains were combined using the following weights to produce the overall Index of Multiple Deprivation:

1. Income Deprivation (22.5%)
2. Employment Deprivation (22.5%)
3. Education, Skills and Training Deprivation (13.5%)
4. Health Deprivation and Disability (13.5%)
5. Crime (9.3%)
6. Barriers to Housing and Services (9.3%)
7. Living Environment Deprivation (9.3%)

**References:**

1. Danaei G, Finucane MM, Lu Y, Singh GM, Cowan MJ, Paciorek CJ, et al. National, regional, and global trends in fasting plasma glucose and diabetes prevalence since 1980: systematic analysis of health examination surveys and epidemiological studies with 370 country-years and 2 7 million participants. The Lancet. 2011;378(9785):31–40. doi: 10.1016/S0140-6736(11)60679-X. pmid:21705069 [↑](#endnote-ref-1)
2. Tran L, Zielinski A, Roach AH, Jende JA, Householder AM, Cole EE, Atway SA, Amornyard M, Accursi ML, Shieh SW, Thompson EE. Pharmacologic treatment of type 2 diabetes: oral medications. Ann Pharmacother. 2015 May;49(5):540-56. doi: 10.1177/1060028014558289. [↑](#endnote-ref-2)
3. Tran L, Zielinski A, Roach AH, Jende JA, Householder AM, Cole EE, Atway SA, Amornyard M, Accursi ML, Shieh SW, Thompson EE. Pharmacologic Treatment of Type 2 Diabetes: Injectable Medications. Ann Pharmacother. 2015 Jun;49(6):700-714. [↑](#endnote-ref-3)
4. Jeppsson JO, Kobold U, Barr J, Finke A, Hoelzel W, Hoshino T, Miedema K, Mosca A, Mauri P, Paroni R, Thienpont L, Umemoto M, Weykamp C; International Federation of Clinical Chemistry and Laboratory Medicine (IFCC). Approved IFCC reference method for the measurement of HbA1c in human blood. Clin Chem Lab Med. 2002 Jan;40(1):78-89. [↑](#endnote-ref-4)
5. de Lusignan S, Crawford L, Munro N. Creating and Using Real World Evidence to Answer Questions about Clinical Effectiveness. Accepted for publication, *Journal of Innovation in Health Informatics 2015.* [↑](#endnote-ref-5)
6. Kenney JT Jr. Managing the evolving complexity of pharmacologic treatment: comparative effectiveness research, pharmacoeconomic data analyses, and other decision support tools. Am J Manag Care. 2012 Nov;18(10 Suppl):S234-9. [↑](#endnote-ref-6)
7. Merck Launches Global Patient Registry Supporting Expanded Commitment to Real-World Outcomes Research in Type 2 DiabetesBusiness Wire, June 13, 2014. Available at:<http://www.businesswire.com/news/home/20140613005029/en/Merck-Launches-Global-Patient-Registry-Supporting-Expanded#.VY5bAflViko> (Accessed: 12 August 2016) [↑](#endnote-ref-7)
8. University of Surrey. Surrey announces new research partnership to explore type 2 diabetes (2015). Available at: <http://www.surrey.ac.uk/features/surrey-announces-new-research-partnership-explore-type-2-diabetes> (Accessed: 12 August 2016) [↑](#endnote-ref-8)
9. Clinical Informatics. The Clinical Informatics and Health Outcomes research group website. Available at: [www.clininf.eu](http://www.clininf.eu) (Accessed: 12 August 2016) [↑](#endnote-ref-9)
10. de Lusignan S, Metsemakers JF, Houwink P, Gunnarsdottir V, van der Lei J. Routinely collected general practice data: goldmines for research? A report of the European Federation for Medical Informatics Primary Care Informatics Working Group (EFMI PCIWG) from MIE2006, Maastricht, The Netherlands. Inform Prim Care. 2006;14(3):203-9. [↑](#endnote-ref-10)
11. Seidu S, Davies MJ, Mostafa S, de Lusignan S, Khunti K. Prevalence and characteristics in coding, classification and diagnosis of diabetes in primary care. Postgrad Med J. 2014 Jan;90(1059):13-7. doi: 10.1136/postgradmedj-2013-132068. [↑](#endnote-ref-11)
12. Hassan Sadek N, Sadek AR, Tahir A, Khunti K, Desombre T, de Lusignan S. Evaluating tools to support a new practical classification of diabetes: excellent control may represent misdiagnosis and omission from disease registers is associated with worse control. Int J Clin Pract. 2012 Sep;66(9):874-82. doi: 10.1111/j.1742-1241.2012.02979.x. [↑](#endnote-ref-12)
13. Health and Social Care Information Centre (HSCIC). Hospital Episode Statistics. Available at: <http://www.hscic.gov.uk/hes> (Accessed: 12 August 2016) [↑](#endnote-ref-13)
14. Jotkowitz, A.B., et al., Do patients with diabetes and low socioeconomic status receive less care and have worse outcomes? A national study. Am J Med, 2006. 119(8): p. 665-9. [↑](#endnote-ref-14)
15. Houle, J., et al., Socioeconomic status and glycemic control in adult patients with type 2 diabetes: a mediation analysis. BMJ Open Diabetes Res Care, 2016. 4(1): p. e000184. [↑](#endnote-ref-15)
16. McGovern A, Hinton W, van Vlymen J, Munro N, Whyte M, de Lusignan S. Real world evidence on the disparities of prescribing of dipeptidyl peptidase-4 inhibitors in UK primary care [Abstract]. Diabetic Medicine (March 2016). 33 (Supplement 1): P183. [↑](#endnote-ref-16)
17. McGovern A, Hinton W, van Vlymen J, Munro N, Whyte M, de Lusignan S. Real world evidence on the prescribing trends in sodium glucose co-transporter 2 inhibitors in UK primary care [Abstract]. Diabetic Medicine (March 2016). 33 (Supplement 1): P165. [↑](#endnote-ref-17)
18. Patel, A., et al., Intensive blood glucose control and vascular outcomes in patients with type 2 diabetes. N Engl J Med, 2008. 358(24): p. 2560-72. [↑](#endnote-ref-18)
19. Gerstein, H.C., et al., Effects of intensive glucose lowering in type 2 diabetes. N Engl J Med, 2008. 358(24): p. 2545-59. [↑](#endnote-ref-19)
20. Kerr, D., et al., HbA1c 3 months after diagnosis predicts premature mortality in patients with new onset type 2 diabetes. Diabet Med, 2011. 28(12): p. 1520-4. [↑](#endnote-ref-20)
21. Inzucchi, S.E., et al., Management of hyperglycemia in type 2 diabetes: a patient-centered approach: position statement of the American Diabetes Association (ADA) and the European Association for the Study of Diabetes (EASD). Diabetes Care, 2012. 35(6): p. 1364-79. [↑](#endnote-ref-21)
22. Hayward, R.A., et al., Follow-up of glycemic control and cardiovascular outcomes in type 2 diabetes. N Engl J Med, 2015. 372(23): p. 2197-206. [↑](#endnote-ref-22)
23. Gerstein, H.C., et al., Effects of intensive glycaemic control on ischaemic heart disease: analysis of data from the randomised, controlled ACCORD trial. Lancet, 2014. 384(9958): p. 1936-41. [↑](#endnote-ref-23)
24. de Lusignan S1, Sadek N, Mulnier H, Tahir A, Russell-Jones D, Khunti K. Miscoding, misclassification and misdiagnosis of diabetes in primary care. Diabet Med. 2012 Feb;29(2):181-9. doi: 10.1111/j.1464-5491.2011.03419.x [↑](#endnote-ref-24)
25. N Hassan Sadek,1 A-R Sadek,1 A Tahir,1 K Khunti,2 T Desombre,1 and S de Lusignan. Evaluating tools to support a new practical classification of diabetes: excellent control may represent misdiagnosis and omission from disease registers is associated with worse control. Int J Clin Pract. 2012 Sep; 66(9): 874–882. doi: 10.1111/j.1742-1241.2012.02979.x [↑](#endnote-ref-25)
26. Stone MA, Camosso-Stefinovic J, Wilkinson J, de Lusignan S, Hattersley AT, Khunti K. Incorrect and incomplete coding and classification of diabetes: a systematic review. Diabet Med. 2010 May;27(5):491-7. doi: 10.1111/j.1464-5491.2009.02920.x. [↑](#endnote-ref-26)
27. Royal College of General Practitioners (RCGP) Research and Surveillance Centre (RSC). Available at: <http://www.rcgp.org.uk/clinical-and-research/research-and-surveillance-centre.aspx> (Accessed: 12 August 2016) [↑](#endnote-ref-27)
28. de Lusignan S. Codes, classifications, terminologies and nomenclatures: definition, development and application in practice. Inform Prim Care. 2005;13(1):65-70. [↑](#endnote-ref-28)
29. de Lusignan S, Mimnagh C. Breaking the first law of informatics: the Quality and Outcomes Framework (QOF) in the dock. Inform Prim Care. 2006;14(3):153-6. [↑](#endnote-ref-29)
30. Apollo Medical Software Solutions (2016). Available at: <http://www.apollo-medical.com/> (Accessed: 12 August 2016) [↑](#endnote-ref-30)
31. Klein, R., et al., The Wisconsin epidemiologic study of diabetic retinopathy. II. Prevalence and risk of diabetic retinopathy when age at diagnosis is less than 30 years. Arch Ophthalmol, 1984. 102(4): p. 520-6. [↑](#endnote-ref-31)
32. Tapp, R.J., et al., The prevalence of and factors associated with diabetic retinopathy in the Australian population. Diabetes Care, 2003. 26(6): p. 1731-7. [↑](#endnote-ref-32)
33. R studio. Available at: <http://www.rstudio.com/> (Accessed: 12 August 2016) [↑](#endnote-ref-33)
34. N. Poh and S. de Lusignan, Modeling Rate of Change in Renal Function for Individual Patients: A Longitudinal Model Based on Routinely Collected Data, Neural Information Processing Systems (NIPS) Personalized Medicine Workshop 2011 (NIPS PM 2011), Sierra Nevada. [↑](#endnote-ref-34)
35. The routine yearly checks in primary care should include an eye check and/or that the patient is attending retinopathy screening or an eye clinic. [↑](#footnote-ref-1)
36. The routine yearly check includes tests of sensation. There should be checks by chiropody and some patients will be under review by hospital teams (e.g. vascular surgeons). [↑](#footnote-ref-2)
37. This is challenging without inclusion of (hospital) HES data. [↑](#footnote-ref-3)
38. Department for Communities and Local Government. The English Indices of Deprivation 2015 (2015). Available at: <https://www.gov.uk/government/statistics/english-indices-of-deprivation-2015> (Accessed: 12 August 2016) [↑](#endnote-ref-35)
