# Supplementary material for: Disparities in glycaemic control, monitoring, and treatment of type 2 diabetes in England: A retrospective cohort analysis
Source: PLoS Med. 2019 Oct 7;16(10):e1002942. doi: 10.1371/journal.pmed.1002942 (PMC6779242; doi:10.1371/journal.pmed.1002942)
Supplement: S2 Text — (DOCX) [file pmed.1002942.s005.docx]

**S2**

Read codes (Version 2) for disease monitoring of glycaemic control (HbA1c), blood pressure, renal function (eGFR), retinopathy and neuropathy.

**HbA1c**

| **Read codes** | **Read code terms** |
| --- | --- |
| 42W4. | HbA1c level (DCCT aligned) |
| 42W5. | Haemoglobin A1c level - International Federation of Clinical Chemistry and Laboratory Medicine standardised |
| 44TB. | Haemoglobin A1c level |
| 44TL. | Total glycosylated haemoglobin level |

**Blood pressure**

| **Read codes** | **Read code terms** |
| --- | --- |
| 246.. | O/E - blood pressure reading |
| 2461 | O/E - BP reading very low |
| 2462 | O/E - BP reading low |
| 2463 | O/E - BP borderline low |
| 2464 | O/E - BP reading normal |
| 2465 | O/E - BP borderline raised |
| 2465 | O/E - BP borderline raised |
| 2466 | O/E - BP reading raised |
| 2467 | O/E - BP reading very high |
| 2468 | O/E - BP reading:postural drop |
| 2469 | O/E - Systolic BP reading |
| 246A. | O/E - Diastolic BP reading |
| 246B. | O/E - BP stable |
| 246J. | O/E - BP reading: no postural drop |
| 246M. | White coat hypertension |
| 246N. | Standing systolic blood pressure |
| 246P. | Standing diastolic blood pressure |
| 246Q. | Sitting systolic blood pressure |
| 246R. | Sitting diastolic blood pressure |
| 246S. | Lying systolic blood pressure |
| 246T. | Lying diastolic blood pressure |
| 246V. | Average 24 hour diastolic blood pressure |
| 246W. | Average 24 hour systolic blood pressure |
| 246X. | Average day interval diastolic blood pressure |
| 246Y. | Average day interval systolic blood pressure |
| 246Z. | O/E-blood pressure reading NOS |
| 246a. | Average night interval diastolic blood pressure |
| 246b. | Average night interval systolic blood pressure |
| 246c. | Average home diastolic blood pressure |
| 246d. | Average home systolic blood pressure |
| 246e. | Ambulatory systolic blood pressure |
| 246f. | Ambulatory diastolic blood pressure |
| 246g. | Self measured blood pressure reading |
| 246k. | Unequal blood pressure in arms |
| 246l. | Average systolic blood pressure |
| 246m. | Average diastolic blood pressure |
| 246n. | Baseline blood pressure |

**eGFR**

“eGFR” is determined from the maximum of eGFR_L1, eGFR_L1_90 +/- eGFR_L1_90_int. However, eGFR is “NA” if eGFR_L1_90 is NULL (i.e. there need to be at least 2x creatinine readings).

eGFR = 141 × min(SCr/K, 1)^a × max(SCr/K, 1)^-1.209 × 0.993^Age × 1.018 [if female] x 1.159 [if black ethnicity].

eGFR = 141 × min(SCr/K, 1)^a × max(SCr/K, 1)^-1.209 × 0.993^Age × 1.018 [if female] x 1.159 [if black ethnicity].

K = 0.7 for females, 0.9 for males

SCr = Serum creatinine

| **Read code** | **Read code term** |
| --- | --- |
| 44J3. | Serum creatinine |
| 44JF. | Plasma creatinine level |

**Retinopathy**

| **Read code** | **Read code term** |
| --- | --- |
| 2BB.. | O/E - retinal inspection |
| 2BB1. | O/E - retina normal |
| 2BB2. | O/E - retinal vessel narrowing |
| 2BB3. | O/E - retinal A-V nipping |
| 2BB4. | O/E - retinal microaneurysms |
| 2BB5. | O/E - retinal haemorrhages |
| 2BB6. | O/E - retinal exudates |
| 2BB7. | O/E - retinal vascular prolif. |
| 2BB8. | O/E - vitreous haemorrhages |
| 2BB9. | O/E - retinal pigmentation |
| 2BBA. | Examination of retina |
| 2BBB. | O/E - Right retina not seen |
| 2BBC. | O/E - Left retina not seen |
| 2BBD. | O/E - Right retina normal |
| 2BBF. | Retinal abnormality - diabetes related |
| 2BBG. | Retinal abnormality - non-diabetes |
| 2BBH. | Retinal drusen |
| 2BBI. | O/E - no retinopathy |
| 2BBJ. | O/E - no right diabetic retinopathy |
| 2BBK. | O/E - no left diabetic retinopathy |
| 2BBL. | O/E - diabetic maculopathy present both eyes |
| 2BBM. | O/E - diabetic maculopathy absent both eyes |
| 2BBN. | Myelinated retinal nerve fibres |
| 2BBO. | O/E - Laser photocoagulation scars |
| 2BBP. | O/E - right eye background diabetic retinopathy |
| 2BBQ. | O/E - left eye background diabetic retinopathy |
| 2BBR. | O/E - right eye preproliferative diabetic retinopathy |
| 2BBS. | O/E - left eye preproliferative diabetic retinopathy |
| 2BBT. | O/E - right eye proliferative diabetic retinopathy |
| 2BBV. | O/E - left eye proliferative diabetic retinopathy |
| 2BBW. | O/E - right eye diabetic maculopathy |
| 2BBX. | O/E - left eye diabetic maculopathy |
| 2BBY. | O/E - referable retinopathy |
| 2BBZ. | O/E - retinal inspection NOS |
| 2BBa. | O/E- non-referable retinopathy |
| 2BBb. | O/E - fundus not adequately seen |
| 2BBc. | O/E - No retinal laser photocoagulation scars |
| 2BBd. | O/E - Red reflex absent |
| 2BBe. | O/E - right retina partially assessable |
| 2BBf. | O/E - left retina partially assessable |
| 2BBg. | O/E - right retina fully assessable |
| 2BBh. | O/E - left retina fully assessable |
| 2BBi. | O/E - right eye no maculopathy |
| 2BBj. | O/E - left eye no maculopathy |
| 2BBk. | O/E - right eye stable treated proliferative diabetic retinopathy |
| 2BBl. | O/E - left eye stable treated proliferative diabetic retinopathy |
| 2BBm. | O/E - right eye clinically significant macular oedema |
| 2BBn. | O/E - left eye clinically significant macular oedema |
| 2BBo. | O/E - sight threatening diabetic retinopathy |
| 2BBr. | Impaired vision due to diabetic retinopathy |
| 2BBs. | Retinal arteries silverwire |
| 2BBp. | On examination right red reflex present |
| 2BBq. | On examination left red reflex present |
| 2BA.. | O/E - optic disc inspection |
| 2BA1. | O/E - optic disc normal |
| 2BA2. | O/E - optic disc pale |
| 2BA3. | O/E - optic disc margin clear |
| 2BA4. | O/E -optic disc margin blurred |
| 2BA5. | O/E - optic disc margin absent |
| 2BA6. | O/E - optic disc flat |
| 2BA7. | O/E - optic disc elevated |
| 2BA8. | O/E - optic disc cupped |
| 2BA9. | O/E - optic disc pigmented |
| 2BAA. | Optic cup/disc ratio |
| 2BAZ. | O/E - optic disc NOS |
| 68A7. | Diabetic retinopathy screening |
| 68A8. | Digital retinal screening |
| 68A9. | Diabetic retinopathy screening offered |
| 68AA. | Digital retinal screening offered |
| 68AB. | Diabetic digital retinopathy screening offered |
| 8HBD. | Retinopathy follow up |
| 8HBG. | Diabetic retinopathy 12 month review |
| 8HBH. | Diabetic retinopathy 6 month review |
| 8Hl1. | Referral for diabetic retinopathy screening |
| 8I3X. | Diabetic retinopathy screening refused |
| 9m0A. | Declined diabetic retinopathy screening |
| F420. | Diabetic retinopathy |
| F4200 | Background diabetic retinopathy |
| F4201 | Proliferative diabetic retinopathy |
| F4202 | Preproliferative diabetic retinopathy |
| F4203 | Advanced diabetic maculopathy |
| F4204 | Diabetic maculopathy |
| F4205 | Advanced diabetic retinal disease |
| F4206 | Non proliferative diabetic retinopathy |
| F4207 | High risk proliferative diabetic retinopathy |
| F4208 | High risk non proliferative diabetic retinopathy |
| F420z | Diabetic retinopathy NOS |

**Neuropathy**

| **Read codes** | **Read code terms** |
| --- | --- |
| C106. | Diabetes with neurological manifestation |
| C109A | Non-insulin dependent diabetes mellitus with mononeuropathy |
| C109B | Non-insulin dependent diabetes mellitus with polyneuropathy |
| C10EC | Type 1 diabetes mellitus with polyneuropathy |
| C10FA | Type 2 diabetes mellitus with mononeuropathy |
| C10FB | Type 2 diabetes mellitus with polyneuropathy |
| F1711 | Autonomic neuropathy due to diabetes |
| F366. | Polyneuropathy |
| F367. | Peripheral neuropathy |
| F372. | Polyneuropathy in diabetes |
| F3720 | Acute painful diabetic neuropathy |
| F3721 | Chronic painful diabetic neuropathy |
| F3722 | Asymptomatic diabetic neuropathy |
| F374z | Polyneuropathy in disease NOS |
| F37y1 | Axonal sensorimotor neuropathy |
| F3y0. | Diabetic mononeuropathy |
| Fyu6B | [X]Other mononeuropathies of lower limb |
| Fyu7C | [X] Polyneuropathy, unspecified |
| FyuAC | [X]Autonomic neuropathy in endocrine and metabolic diseases classified elsewhere |
| M2711 | Neuropathic diabetic ulcer - foot |
| C1060 | Diabetes mellitus, juvenile type, with neurological manifestation |
| C1061 | Diabetes mellitus, adult onset, with neurological manifestation |
| C106y | Other specified diabetes mellitus with neurological complications |
| C106z | Diabetes mellitus NOS with neurological manifestation |
| 311A. | Monofilament foot sensation test |
| 29B.. | O/E - tactile sensation |
| 29B1. | O/E - tactile sensation normal |
| 29B2. | O/E - anaesthesia present |
| 29B20 | O/E - anaesthesia in legs |
| 29B21 | O/E - anaesthesia of extremities |
| 29B3. | O/E - hypoaesthesia present |
| 29B4. | O/E - hyperaesthesia present |
| 29B5. | O/E - paraesthesia present |
| 29B50 | O/E - paraesthesia in hands |
| 29B6. | Hemisensory loss |
| 29B7. | 10g monofilament sensation present |
| 29B8. | 10g monofilament sensation absent |
| 29B9. | 10g monofilament sensation R foot abnormal |
| 29BA. | 10g monofilament sensation L foot abnormal |
| 29BB. | 10g monofilament sensation R foot normal |
| 29BC. | 10g monofilament sensation L foot normal |
| 29BD. | 10g monofilament sensation plantar aspect of great toe left foot present |
| 29BE. | 10g monofilament sensation plantar aspect of middle toe right foot present |
| 29BF. | 10g monofilament sensation plantar aspect of middle toe left foot present |
| 29BG. | 10g monofilament sensation plantar aspect of little toe right foot present |
| 29BH. | 10g monofilament sensation plantar aspect of little toe left foot present |
| 29BJ. | 10g monofilament sensation plantar aspect of first metatarsal head right foot present |
| 29BK. | 10g monofilament sensation plantar aspect of first metatarsal head left foot present |
| 29BL. | 10g monofilament sensation plantar aspect of great toe right foot present |
| 29BM. | 10g monofilament sensation plantar aspect of great toe right foot absent |
| 29BN. | 10g monofilament sensation plantar aspect of great toe left foot absent |
| 29BP. | 10g monofilament sensation plantar aspect of middle toe right foot absent |
| 29BQ. | 10g monofilament sensation plantar aspect of middle toe left foot absent |
| 29BR. | 10g monofilament sensation plantar aspect of little toe right foot absent |
| 29BS. | 10g monofilament sensation plantar aspect of little toe left foot absent |
| 29BT. | 10g monofilament sensation plantar aspect of first metatarsal head right foot absent |
| 29BV. | 10g monofilament sensation plantar aspect of first metatarsal head left foot absent |
| 29C.. | O/E - tactile discrimination |
| 29C1. | O/E-tactile discrimination NAD |
| 29C2. | O/E - tactile discrimination.abnormal |
| 29C3. | Pin prick sensation of right foot abnormal |
| 29C4. | Pin prick sensation of left foot abnormal |
| 29C5. | Pin prick sensation of right foot normal |
| 29C6. | Pin prick sensation of left foot normal |
| 29C7. | Tactile discrimination right foot abnormal |
| 29C8. | Tactile discrimination left foot abnormal |
| 29C9. | Tactile discrimination right foot normal |
| 29CA. | Tactile discrimination left foot normal |
| 29CZ. | O/E-tactile discrimination NOS |
| 29D.. | O/E - deep sensation |
| 29D1. | O/E - deep sensation normal |
| 29D2. | O/E - deep sensation reduced |
| 29D3. | O/E - deep sensation absent |
| 29DZ. | O/E - deep sensation NOS |
| 29E.. | O/E - pain sensation |
| 29E1. | O/E - pain sensation normal |
| 29E2. | O/E - pain sensation reduced |
| 29E3. | O/E - analgesia present |
| 29E4. | On examination allodynia |
| 29EZ. | O/E - pain sensation NOS |
| 29F.. | O/E-temperature discrimination |
| 29F1. | O/E - temp. discrimination NAD |
| 29F2. | O/E - temp. discrimination. Reduced |
| 29F3. | O/E - temp. discrimination. Absent |
| 29FZ. | O/E - temp. discrimination NOS |
| 29G.. | O/E - joint position sense |
| 29G1. | O/E - joint position. sense normal |
| 29G2. | O/E - joint position.sense reduced |
| 29G3. | O/E - joint position.sense absent |
| 29GZ. | O/E - joint position. sense NOS |
| 29H.. | O/E - vibration sense |
| 29H1. | O/E - vibration sense normal |
| 29H2. | O/E - vibration sense reduced |
| 29H3. | O/E - vibration sense absent |
| 29H4. | O/E - Vibration sense of right foot abnormal |
| 29H5. | O/E - Vibration sense of right foot normal |
| 29H6. | O/E - Vibration sense of left foot abnormal |
| 29H7. | O/E - Vibration sense of left foot normal |
| 29H8. | O/E - vibration sense left foot reduced |
| 29H9. | O/E - vibration sense right foot reduced |
| 29HA. | O/E - Vibration sense of right foot absent |
| 29HB. | O/E - Vibration sense of left foot absent |
| 29HZ. | O/E - vibration sense NOS |
