# Supplementary material for: Disparities in glycaemic control, monitoring, and treatment of type 2 diabetes in England: A retrospective cohort analysis
Source: PLoS Med. 2019 Oct 7;16(10):e1002942. doi: 10.1371/journal.pmed.1002942 (PMC6779242; doi:10.1371/journal.pmed.1002942)
Supplement: S3 Text — (DOCX) [file pmed.1002942.s006.docx]

**S3 Appendix**

- Patient age
- Patient gender
- Patient ethnicity (White, Asian, Black, Mixed, none, other)
- Smoking status (never smoked, active smoker, ex-smoker, unknown)
- Alcohol use (non-drinker, safe use [<14 units per week], hazardous use, alcoholism, none)
- Duration of diabetes (< 5 years, 5-9 years, 10-14 years, 15-19 years, ≥ 20 years)
- Glycaemic control (HbA_1c_ – most recent before start of follow up period)
- Concurrent diabetes therapies (between 2012 and 2016)
- Comorbidities and complications
  - Amputation
  - Angina
  - Congestive cardiac failure
  - Hypertension
  - Peripheral artery disease
  - Renal replacement
  - Renal impairment (categorised by stage; 3a, 3b, 4, and 5)
  - Ischaemic heart disease
  - Previous stroke/transient ischaemic attack
  - Atrial fibrillation
  - Retinopathy
- Other parameters
  - Blood pressure
    - Systolic (<120mmHg, 120-140mmHg, 140-159mmHg, ≥ 160mmHg, missing)
    - Diastolic (<80mmHg, 80-89mmHg, 90-99mmHg, ≥ 100mmHg, missing)
  - BMI (underweight [<18.5 kg/m^2^], normal weight [18.5-24.9], overweight [25-29.9], obese class I [30-34.9], obese class I [35-39.9], obese class III [≥40])
