# Supplementary material for: Disparities in glycaemic control, monitoring, and treatment of type 2 diabetes in England: A retrospective cohort analysis
Source: PLoS Med. 2019 Oct 7;16(10):e1002942. doi: 10.1371/journal.pmed.1002942 (PMC6779242; doi:10.1371/journal.pmed.1002942)
Supplement: S4 Text — (PDF) [file pmed.1002942.s007.pdf]

Category	Description	Definition
Alcoholism	Coded as having alcohol dependence or complications of alcohol use	Any code in the alcoholism code list ever recorded
Hazardous	Current or history of drinking more than the recommended limits or reversible complications of alcohol use	No alcoholism code ever **AND** (Any code in the hazardous drinking category ever **OR** ever had value associated with a = "136V" readcode > 14 (values >1000 should be ignored) **OR** ever had a value associated with a = "136" readcode > 14 (values >1000 should be ignored))
Safe	Drinking but within the recommended limits and no history of excess alcohol use	No alcoholism or hazardous drinking codes ever **AND** most recent of the following is consistent with safe drinking (code from the list of safe and non-drinking codes **OR** 136/136V code [value > 0 and <= 14])
Non-drinker	Currently a non-drinker and no history of excess alcohol use	No alcoholism or hazardous drinking codes ever **AND** most recent of the following is consistent with non-drinking (code from the list of safe and non-drinking codes **OR** 136/136V code [value = 0])

Alcoholism/complications		
1365.	0	Heavy drinker - 7-9u/day
1366.	0	Very heavy drinker - >9u/day
136D.	0	Ex-heavy drinker - (7-9u/day)
136E.	0	Ex-very heavy drinker-(>9u/d)
136P.	0	Heavy drinker
136Q.	0	Very heavy drinker
136T.	0	Harmful alcohol use
136W.	0	Alcohol misuse
136Y.	0	Drinks in morning to get rid of hangover
13Y8.	0	Alcoholics anonymous
1B1c.	0	Alcohol induced hallucinations
1F9D.	0	Replaces meals with drinks
66e%	0	Alcohol disorder monitoring
7P221	0	Delivery of rehabilitation for alcohol addiction
8BA8.	0	Alcohol detoxification
8BAs.	0	Alcohol relapse prevention
8BAu.	0	Alcohol harm reduction programme
8BAw.	0	Alcohol twelve step programme
8H35.	0	Admitted to alcohol detoxification centre
8IAt.	0	Extended intervention for excessive alcohol consumption declined
9k1B.	0	Extended intervention for excessive alcohol consumption completed
9NgzH	0	Withdrawn from alcohol detoxification programme
9NJz.	0	In-house alcohol detoxification
C1505	0	Alcohol-induced pseudo-Cushing's syndrome
du1%	0	DISULFIRAM
du5%	0	ACAMPROSATE CALCIUM
E01%	0	Alcoholic psychoses
E23%	12	Alcohol problem drinking

E250%	0	Nondependent alcohol abuse
Eu10%	0	[X]Mental and behavioural disorders due to use of alcohol
F11x0	0	Cerebral degeneration due to alcoholism
F1440	0	Cerebellar ataxia due to alcoholism
F25B.	0	Alcohol-induced epilepsy
F375.	0	Alcoholic polyneuropathy
G555.	0	Alcoholic cardiomyopathy
G8523	0	Oesophageal varices in alcoholic cirrhosis of the liver
J612%	0	Alcoholic cirrhosis of liver
J6130	0	Alcoholic hepatic failure
J617.	0	Alcoholic hepatitis
J6170	0	Chronic alcoholic hepatitis
J6710	0	Alcohol-induced chronic pancreatitis
U60H3	11	[X] Adverse reaction to alcohol deterrents
U60H3	0	[X]Alcohol deterrents causing adverse effects in therapeutic use
ZV57A	0	[V]Alcohol rehabilitation
ZV6D6	0	[V]Alcohol abuse counselling and surveillance
ACE/28581	EMIS	Acamprosate 333mg gastro-resistant tablets
ACE/28581	EMIS	Acamprosate Calcium E/C Tablets 333 mg
ACE/28581	EMIS	acamprosate calcium enteric coated tablets 333mg
DITA4859		Disulfiram Tablets 200 mg
DITA4859		Disulfiram 200mg tablets
66e0.		Alcohol abuse monitoring
du11.		DISULFIRAM 200mg tablets
du12.		ANTABUSE 200mg tablets
du51.		ACAMPROSATE CALCIUM 333mg e/c tablets
du52.		CAMPRAL EC 333mg e/c tablets
E010.		Alcohol withdrawal delirium
E011.		Alcohol amnestic syndrome
E0110		Korsakov's alcoholic psychosis
E0111		Korsakov's alcoholic psychosis with peripheral neuritis
E0112		Wernicke-Korsakov syndrome
E011z		Alcohol amnestic syndrome NOS
E012.		Other alcoholic dementia
E0120		Chronic alcoholic brain syndrome
E013.		Alcohol withdrawal hallucinosis
E014.		Pathological alcohol intoxication
E015.		Alcoholic paranoia
E01y.		Other alcoholic psychosis
E01y0		Alcohol withdrawal syndrome
E01yz		Other alcoholic psychosis NOS
E01z.		Alcoholic psychosis NOS
E230.		Acute alcoholic intoxication in alcoholism
E2300		Acute alcoholic intoxication, unspecified, in alcoholism

E2301	Continuous acute alcoholic intoxication in alcoholism
E2302	Episodic acute alcoholic intoxication in alcoholism
E2303	Acute alcoholic intoxication in remission, in alcoholism
E230z	Acute alcoholic intoxication in alcoholism NOS
E231.	Chronic alcoholism
E2310	Unspecified chronic alcoholism
E2311	Continuous chronic alcoholism
E2312	Episodic chronic alcoholism
E2313	Chronic alcoholism in remission
E231z	Chronic alcoholism NOS
E23z.	Alcohol dependence syndrome NOS
E2500	Nondependent alcohol abuse, unspecified
E2501	Nondependent alcohol abuse, continuous
E2502	Nondependent alcohol abuse, episodic
E2503	Nondependent alcohol abuse in remission
E250z	Nondependent alcohol abuse NOS
Eu100	[X]Mental and behavioural disorders due to use of alcohol: acute intoxication
Eu101	[X]Mental and behavioural disorders due to use of alcohol: harmful use
Eu102	[X]Mental and behavioural disorders due to use of alcohol: dependence syndrome

**Hazardous drinking**		
1364.	0	Moderate drinker - 3-6u/day
136a.	0	Increasing risk drinking
136b.	0	Feels should cut down drinking
136c.	0	Higher risk drinking
136K.	0	Alcohol intake above recommended sensible limits
136O.	0	Moderate drinker
136R.	0	Binge drinker
136S.	0	Hazardous alcohol use
136X.	0	Alcohol units consumed on heaviest drinking day
13ZY.	0	Disqualified from driving due to excess alcohol
2577.	11	O/E - alcoholic breath
38Dz.	0	Severity of alcohol dependence questionnaire
6792.	0	Health ed. - alcohol
67K6.	0	Cycle of change stage, alcohol
8CdK.	0	Specialist alcohol treatment service signposted
8CE1.	0	Alcohol leaflet given
8H7p.	0	Referral to community alcohol team
8HkG.	0	Referral to specialist alcohol treatment service
8IAF.	0	Brief intervention for excessive alcohol consumption declined
8IAJ.	0	Declined referral to specialist alcohol treatment service
8IEA.	0	Referral to community alcohol team declined
9EVD.	0	Hospital alcohol liaison team report received
9k11.	0	Alcohol consumption counselling
9k14.	0	Alcohol counselling by other agencies

9k1A.	0	Brief intervention for excessive alcohol consumption completed
9NN2.	0	Under care of community alcohol team
9Nz9.	0	Emergency department attendance related to personal alcohol consumption
9NzA.	0	Hospital attendance related to personal alcohol consumption
J153.	0	Alcoholic gastritis
J610.	0	Alcoholic fatty liver
J611.	0	Acute alcoholic hepatitis
J613.	0	Alcoholic liver damage unspecified
J6708	0	Alcohol-induced acute pancreatitis
R103.	0	[D]Alcohol blood level excessive
SLH3.	0	Alcohol deterrent poisoning
SM000	0	Ethanol causing toxic effect
U209.	0	[X]Intentional self poisoning by and exposure to alcohol

**Safe**		
ZV791	0	[V]Screening for alcoholism
136Z.	0	Alcohol consumption NOS
136L.	0	Alcohol intake within recommended sensible limits
136G.	0	Beer drinker
136H.	0	Drinks beer and spirits
136I.	0	Drinks wine
388u.	0	Fast alcohol screening test
38Df.	0	Five-shot questionnaire on heavy drinking
67H0.	0	Lifestyle advice regarding alcohol
136N.	0	Light drinker
1363.	0	Light drinker - 1-2u/day
136d.	0	Lower risk drinking
1D19.	0	Pain in lymph nodes after alcohol consumption
8CAM.	0	Patient advised about alcohol
136J.	0	Social drinker
136F.	0	Spirit drinker
1362.	0	Trivial drinker - <1u/day

**Non-drinker**		
136M.	0	Current non drinker
136A.	0	Ex-trivial drinker (<1u/day)
1361.	0	Teetotaller
