# Supplementary material for: Disparities in glycaemic control, monitoring, and treatment of type 2 diabetes in England: A retrospective cohort analysis
Source: PLoS Med. 2019 Oct 7;16(10):e1002942. doi: 10.1371/journal.pmed.1002942 (PMC6779242; doi:10.1371/journal.pmed.1002942)
Supplement: S5 Text — (DOCX) [file pmed.1002942.s008.docx]

**S5 Appendix**

Complications and comorbidities defined using a comprehensive list of diagnosis, investigation, and process of care Read codes (version 2).

**Amputation**

| **Read code** | **Read code term** |
| --- | --- |
| 14N4. | H/O: limb amputation |
| 14N40 | H/O: upper limb amputation |
| 14N41 | H/O: lower limb amputation |
| 14N4Z | H/O: limb amputation NOS |
| 7L04. | Amputation of arm |
| 7L040 | Forequarter amputation |
| 7L041 | Disarticulation of shoulder |
| 7L042 | Amputation above elbow |
| 7L043 | Amputation through forearm |
| 7L044 | Amputation through elbow |
| 7L04y | Other specified amputation of arm |
| 7L04z | Amputation of arm NOS |
| 7L05. | Amputation of hand |
| 7L050 | Amputation through wrist |
| 7L051 | Amputation of thumb |
| 7L052 | Amputation of phalanx of finger |
| 7L053 | Amputation of finger NEC |
| 7L054 | Amputation through phalanx of finger |
| 7L055 | Terminalisation of thumb |
| 7L056 | Terminalisation of finger |
| 7L05y | Other specified amputation of hand |
| 7L05z | Amputation of hand NOS |
| 7L06. | Amputation of leg |
| 7L060 | Hindquarter amputation |
| 7L061 | Disarticulation of hip |
| 7L062 | Amputation above knee |
| 7L063 | Amputation through knee |
| 7L064 | Amputation below knee |
| 7L06y | Other specified amputation of leg |
| 7L06z | Amputation of leg NOS |
| 7L07. | Amputation of foot |
| 7L070 | Amputation through ankle |
| 7L071 | Disarticulation of tarsal bones |
| 7L072 | Disarticulation tarsometatarsal joint |
| 7L073 | Amputation through metatarsal bones |
| 7L07y | Other specified amputation of foot |
| 7L07z | Amputation of foot NOS |
| 7L08. | Amputation of toe |
| 7L080 | Amputation hallux |
| 7L081 | Amputation of phalanx of toe |
| 7L082 | Proximal hemiphalangectomy of toe |
| 7L083 | Amputation lesser toe |
| 7L084 | Terminalisation of hallux |
| 7L085 | Terminalisation of lesser toe |
| 7L08y | Other specified amputation of toe |
| 7L08z | Amputation of toe NOS |
| 7L09. | Operations on amputation stump |
| 7L090 | Reamputation at higher level |
| 7L091 | Excision of lesion of amputation stump |
| 7L092 | Shortening of amputation stump |
| 7L093 | Revision coverage of amputation stump |
| 7L094 | Drainage of amputation stump |
| 7L095 | Excision neuroma amputation stump |
| 7L09y | Other specified operation on amputation stump |
| 7L09z | Operation on amputation stump NOS |
| F336. | Phantom limb syndrome |
| F3360 | Phantom limb syndrome with pain |
| F3361 | Phantom limb syndrome without pain |
| SP16. | Late amputation stump complication |
| SP160 | Late amputation stump complication, unspecified |
| SP161 | Neuroma of amputation stump |
| SP162 | Chronic infection of amputation stump |
| SP163 | Necrosis of amputation stump |
| SP164 | Bony prominence in amputation stump |
| SP165 | Poorly shaped amputation stump |
| SP16z | Late amputation stump complication NOS |

**Angina**

| **Read code** | **Read code term** |
| --- | --- |
| 14A5. | H/O: angina pectoris |
| 662K. | Angina control |
| 662K0 | Angina control - good |
| 662K1 | Angina control - poor |
| 662K2 | Angina control - improving |
| 662K3 | Angina control - worsening |
| 662K4 | Angina self management plan commenced |
| 662K5 | Angina self management plan completed |
| 662Kz | Angina control NOS |
| G3111 | Unstable angina |
| G3112 | Angina at rest |
| G3113 | Refractory angina |
| G3114 | Worsening angina |
| G33.. | Angina pectoris |
| G330. | Angina decubitus |
| G3300 | Nocturnal angina |
| G330z | Angina decubitus NOS |
| G331. | Prinzmetal's angina |
| G332. | Coronary artery spasm |
| G33z. | Angina pectoris NOS |
| G33z0 | Status anginosus |
| G33z1 | Stenocardia |
| G33z2 | Syncope anginosa |
| G33z3 | Angina on effort |
| G33z4 | Ischaemic chest pain |
| G33z5 | Post infarct angina |
| G33z6 | New onset angina |
| G33z7 | Stable angina |
| G33zz | Angina pectoris NOS |

**Congestive cardiac failure**

| **Read code** | **Read code term** |
| --- | --- |
| 662f. | New York Heart Association classification - class I |
| 662g. | New York Heart Association classification - class II |
| 662h. | New York Heart Association classification - class III |
| 662i. | New York Heart Association classification - class IV |
| G1yz1 | Rheumatic left ventricular failure |
| G58.. | Heart failure |
| G58.. | Cardiac failure |
| G580. | Congestive heart failure |
| G580. | Congestive cardiac failure |
| G580. | Right heart failure |
| G580. | Right ventricular failure |
| G580. | Biventricular failure |
| G5800 | Acute congestive heart failure |
| G5801 | Chronic congestive heart failure |
| G5802 | Decompensated cardiac failure |
| G5803 | Compensated cardiac failure |
| G5804 | Congestive heart failure due to valvular disease |
| G581. | Left ventricular failure |
| G581. | Asthma - cardiac |
| G581. | Pulmonary oedema - acute |
| G581. | Impaired left ventricular function |
| G5810 | Acute left ventricular failure |
| G582. | Acute heart failure |
| G583. | Heart failure with normal ejection fraction |
| G583. | HFNEF - heart failure with normal ejection fraction |
| G583. | Heart failure with preserved ejection fraction |
| G584. | Right ventricular failure |
| G58z. | Heart failure NOS |
| G58z. | Weak heart |
| G58z. | Cardiac failure NOS |
| 14A6. | H/O: heart failure |
| 14AM. | H/O: Heart failure in last year |
| 14S3. | H/O: heart recipient |
| 14T7. | H/O: artificial heart |
| 1O1.. | Heart failure confirmed |
| 662p. | Heart failure 6 month review |
| 662T. | Congestive heart failure monitoring |
| 662W. | Heart failure annual review |
| 679X. | Heart failure education |
| 679W1 | Education about deteriorating heart failure |
| 67D4. | Heart failure information given to patient |
| 7900 | Transplantation of heart and lung |
| 79000 | Allotransplantation of heart and lung |
| 79001 | Revision of transplantation of heart and lung |
| 7900y | Other specified transplantation of heart and lung |
| 7900z | Transplantation of heart and lung NOS |
| 7901 | Other transplantation of heart |
| 79010 | Allotransplantation of heart NEC |
| 79011 | Xenotransplantation of heart |
| 79013 | Piggyback transplantation of heart |
| 79014 | Revision of implantation of prosthetic heart |
| 79015 | Revision of transplantation of heart NEC |
| 7901y | Other specified other transplantation of heart |
| 7901z | Other transplantation of heart NOS |
| 79379 | Implantation of biventricular cardiac pacemaker system |
| 7936J | Implantation of intravenous biventricular cardiac pacemaker system |
| 7933 | Transluminal heart assist operations |
| 79330 | Transluminal insertion of pulsation balloon into aorta |
| 79331 | Transluminal insertion of heart assist system NEC |
| 79332 | Transluminal maintenance of heart assist system |
| 79334 | Implantation of ventricular assist device |
| 79335 | Implantation of right ventricular assist device |
| 79336 | Implantation of left ventricular assist device |
| 79337 | Implantation of biventricular assist device |
| 7933y | Other specified transluminal heart assist operation |
| 7933z | Transluminal heart assist operation NOS |
| 793L. | Open heart assist operations |
| 793L0 | Open implantation of ventricular assist device |
| 793Ly | Other specified open heart assist operations |
| 793Lz | Open heart assist operations NOS |
| 8B29. | Cardiac failure therapy |
| 8CeC. | Preferred place of care for next exacerbation of heart failure |
| 8CL3. | Heart failure care plan discussed with patient |
| 8CMK. | Has heart failure management plan |
| 8CMW8 | Heart failure clinical pathway |
| 8H2S. | Admit heart failure emergency |
| 8HBE. | Heart failure follow-up |
| 8HHb. | Referral to heart failure nurse |
| 8HHz. | Referral to heart failure exercise programme |
| 8Hk0. | Referred to heart failure education group |
| 8HTL. | Referral to heart failure clinic |
| 8HTL0 | Referral to rapid access heart failure clinic |
| 9Or0. | Heart failure review completed |
| 9N0k. | Seen in heart failure clinic |
| 9N2p. | Seen by community heart failure nurse |
| G2101 | Malignant hypertensive heart disease with congestive cardiac failure |
| G2111 | Benign hypertensive heart disease with congestive cardiac failure |
| G21z1 | Hypertensive heart disease NOS with congestive cardiac failure |
| G232. | Hypertensive heart and renal disease with (congestive) heart failure |
| G234. | Hypertensive heart and renal disease with both (congestive) heart failure and renal failure |
| Q48y1 | Congenital cardiac failure |
| Q490. | Neonatal cardiac failure |
| SP084 | Heart transplant failure and rejection |
| SP085 | Heart-lung transplant failure and rejection |
| TB000 | Transplantation of heart as the cause of abnormal reaction of patient, or of later complication, without mention of misadventure at the time of operation |
| ZV421 | [V]Heart transplanted |
| ZV45M | [V]Biventricular pacemaker in situ |

**Hypertension**

| **Read code** | **Read code term** |
| --- | --- |
| 14A2. | H/O: hypertension |
| 662b. | Moderate hypertension control |
| 662c. | Hypertension six month review |
| 662d. | Hypertension annual review |
| 6627 | Good hypertension control |
| 6628 | Poor hypertension control |
| 662F. | Hypertension treatm. started |
| 662G. | Hypertensive treatm.changed |
| 662O. | On treatment for hypertension |
| 662P. | Hypertension monitoring |
| 662P0 | Hypertension 9 month review |
| 662P1 | Telehealth hypertension monitoring |
| 67H8. | Lifestyle advice regarding hypertension |
| 8B26. | Antihypertensive therapy |
| 8HT5. | Referral to hypertension clinic |
| 9N03. | Seen in hypertension clinic |
| 9OI1. | Attends hypertension monitor. |
| G2... | Hypertensive disease |
| G2... | BP - hypertensive disease |
| G20.. | Essential hypertension |
| G20.. | High blood pressure |
| G20.. | Primary hypertension |
| G200. | Malignant essential hypertension |
| G201. | Benign essential hypertension |
| G202. | Systolic hypertension |
| G203. | Diastolic hypertension |
| G20z. | Essential hypertension NOS |
| G20z. | Hypertension NOS |
| G21.. | Hypertensive heart disease |
| G210. | Malignant hypertensive heart disease |
| G2100 | Malignant hypertensive heart disease without congestive cardiac failure |
| G2101 | Malignant hypertensive heart disease with congestive cardiac failure |
| G210z | Malignant hypertensive heart disease NOS |
| G211. | Benign hypertensive heart disease |
| G2110 | Benign hypertensive heart disease without congestive cardiac failure |
| G2111 | Benign hypertensive heart disease with congestive cardiac failure |
| G211z | Benign hypertensive heart disease NOS |
| G21z. | Hypertensive heart disease NOS |
| G21z0 | Hypertensive heart disease NOS without congestive cardic failure |
| G21z0 | Cardiomegaly - hypertensive |
| G21z1 | Hypertensive heart disease NOS with congestive cardiac failure |
| G21zz | Hypertensive heart disease NOS |
| G22.. | Hypertensive renal disease |
| G22.. | Nephrosclerosis |
| G220. | Malignant hypertensive renal disease |
| G221. | Benign hypertensive renal disease |
| G222. | Hypertensive renal disease with renal failure |
| G22z. | Hypertensive renal disease NOS |
| G22z. | Renal hypertension |
| G23.. | Hypertensive heart and renal disease |
| G230. | Malignant hypertensive heart and renal disease |
| G231. | Benign hypertensive heart and renal disease |
| G232. | Hypertensive heart and renal disease with (congestive) heart failure |
| G233. | Hypertensive heart and renal disease with renal failure |
| G234. | Hypertensive heart and renal disease with both (congestive) heart failure and renal failure |
| G23z. | Hypertensive heart and renal disease NOS |
| G24.. | Secondary hypertension |
| G240. | Secondary malignant hypertension |
| G2400 | Secondary malignant renovascular hypertension |
| G240z | Secondary malignant hypertension NOS |
| G241. | Secondary benign hypertension |
| G2410 | Secondary benign renovascular hypertension |
| G241z | Secondary benign hypertension NOS |
| G244. | Hypertension secondary to endocrine disorders |
| G24z. | Secondary hypertension NOS |
| G24z0 | Secondary renovascular hypertension NOS |
| G24zz | Secondary hypertension NOS |
| G25.. | Stage 1 hypertension (NICE - National Institute for Health and Clinical Excellence 2011) |
| G25.. | Stage 1 hypertension |
| G26.. | Severe hypertension (NICE - National Institute for Health and Clinical Excellence 2011) |
| G26.. | Severe hypertension |
| G27.. | Hypertension resistant to drug therapy |
| G28.. | Stage 2 hypertension (NICE - National Institute for Health and Clinical Excellence 2011) |
| G2y.. | Other specified hypertensive disease |
| G2z.. | Hypertensive disease NOS |
| G672. | Hypertensive encephalopathy |
| G672. | Hypertensive crisis |
| F4213 | Hypertensive retinopathy |
| L1200 | Benign essential hypertension complicating pregnancy, childbirth and the puerperium unspecified |
| L122. | Other pre-existing hypertension in preg/childbirth/puerp |
| L1220 | Other pre-existing hypertension complicating pregnancy, childbirth and the puerperium unspecified |
| L1221 | Other pre-existing hypertension complicating pregnancy, childbirth and the puerperium - delivered |
| L1222 | Other pre-existing hypertension complicating pregnancy, childbirth and the puerperium - delivered with postnatal complication |
| L1223 | Other pre-existing hypertension complicating pregnancy, childbirth and the puerperium - not delivered |
| L1224 | Other pre-existing hypertension complicating pregnancy, childbirth and the puerperium with postnatal complication |
| L122z | Other pre-existing hypertension complicating pregnancy, childbirth and the puerperium NOS |
| L128. | Pre-existing hypertension complicating pregnancy, childbirth and puerperium |
| L1280 | Pre-existing hypertensive heart disease complicating pregnancy, childbirth and the puerperium |
| L1281 | Pre-existing hypertensive heart and renal disease complicating pregnancy, childbirth and the puerperium |
| L1282 | Pre-existing secondary hypertension complicating pregnancy, childbirth and puerperium |
| Gyu2. | [X]Hypertensive diseases |
| Gyu20 | [X]Other secondary hypertension |
| Gyu21 | [X]Hypertension secondary to other renal disorders |

**Peripheral arterial disease**

| **Read code** | **Read code term** |
| --- | --- |
| 7A41. | Other bypass of iliac artery |
| 7A410 | Emergency bypass of iliac artery by anastomosis of iliac artery to femoral artery NEC |
| 7A411 | Bypass of iliac artery by anastomosis of iliac artery to femoral artery NEC |
| 7A412 | Emergency bypass of iliac artery by anastomosis of femoral artery to femoral artery NEC |
| 7A413 | Bypass of iliac artery by anastomosis of femoral artery to femoral artery NEC |
| 7A414 | Emergency bypass of common iliac artery by anastomosis of aorta to common iliac artery NEC |
| 7A415 | Emergency bypass of iliac artery by anastomosis of aorta to external iliac artery NEC |
| 7A416 | Emergency bypass of artery of leg by anastomosis of aorta to common femoral artery NEC |
| 7A417 | Emergency bypass of artery of leg by anastomosis of aorta to deep femoral artery NEC |
| 7A418 | Emergency bypass of iliac artery by anastomosis of iliac artery to iliac artery NEC |
| 7A419 | Bypass of common iliac artery by anastomosis of aorta to common iliac artery NEC |
| 7A41A | Bypass of iliac artery by anastomosis of aorta to external iliac NEC |
| 7A41B | Bypass of artery of leg by anastomosis of aorta to common femoral artery NEC |
| 7A41C | Bypass of artery of leg by anastomosis of aorta to deep femoral artery NEC |
| 7A41D | Bypass of iliac artery by anastomosis of iliac artery to iliac artery NEC |
| 7A41E | Emergency bypass of iliac artery by unspecified anastomosis |
| 7A41F | Ilio-femoral prosthetic cross over graft |
| 7A41y | Other specified other bypass of iliac artery |
| 7A41z | Other bypass of iliac artery NOS |
| 7A42. | Reconstruction of iliac artery |
| 7A420 | Endarterectomy of iliac artery and patch repair of iliac artery |
| 7A421 | Endarterectomy of iliac artery NEC |
| 7A42y | Other specified reconstruction of iliac artery |
| 7A42z | Reconstruction of iliac artery NOS |
| 7A43. | Other open operations on iliac artery |
| 7A430 | Repair of iliac artery NEC |
| 7A431 | Open embolectomy of iliac artery |
| 7A433 | Open insertion of iliac artery stent |
| 7A43y | Other specified other open operation on iliac artery |
| 7A43z | Other open operation on iliac artery NOS |
| 7A44. | Transluminal operations on iliac artery |
| 7A440 | Percutaneous transluminal angioplasty of iliac artery |
| 7A441 | Percutaneous transluminal embolectomy of iliac artery |
| 7A442 | Arteriography of iliac artery |
| 7A443 | Insertion of iliac artery stent |
| 7A444 | Percutaneous transluminal insertion of iliac artery stent |
| 7A44y | Other specified transluminal operation on iliac artery |
| 7A44z | Transluminal operation on iliac artery NOS |
| 7A47. | Other emergency bypass of femoral artery or popliteal artery |
| 7A470 | Emergency bypass of femoral artery by anastomosis of femoral artery to popliteal artery using prosthesis NEC |
| 7A471 | Emergency bypass of popliteal artery by anastomosis of popliteal artery to popliteal artery using prosthesis NEC |
| 7A472 | Emergency bypass of femoral artery by anastomosis of femoral artery to popliteal artery using vein graft NEC |
| 7A473 | Emergency bypass of popliteal artery by anastomosis of popliteal artery to popliteal artery using vein graft NEC |
| 7A474 | Emergency bypass of femoral artery by anastomosis of femoral artery to tibial artery using prosthesis NEC |
| 7A475 | Emergency bypass of popliteal artery by anastomosis of popliteal artery to tibial artery using prosthesis NEC |
| 7A476 | Emergency bypass of femoral artery by anastomosis of femoral artery to tibial artery using vein graft NEC |
| 7A477 | Emergency bypass of popliteal artery by anastomosis of popliteal artery to tibial artery using vein graft NEC |
| 7A478 | Emergency bypass of femoral artery by anastomosis of femoral artery to peroneal artery using prosthesis NEC |
| 7A479 | Emergency bypass of popliteal artery by anastomosis of popliteal artery to peroneal artery using prosthesis NEC |
| 7A47A | Emergency bypass of femoral artery by anastomosis of femoral artery to peroneal artery using vein graft NEC |
| 7A47B | Emergency bypass of popliteal artery by anastomosis of popliteal artery to peroneal artery using vein graft NEC |
| 7A47C | Emergency bypass of femoral artery by anastomosis of femoral artery to femoral artery NEC |
| 7A47D | Emergency bypass of popliteal artery by anastomosis of popliteal artery to femoral artery NEC |
| 7A47y | Other specified other emergency bypass of femoral artery or popliteal artery |
| 7A47z | Other emergency bypass of femoral artery or popliteal artery NOS |
| 7A48. | Other bypass of femoral artery or popliteal artery |
| 7A480 | Bypass of femoral artery by anastomosis of femoral artery to popliteal artery using prosthesis NEC |
| 7A481 | Bypass of popliteal artery by anastomosis of popliteal artery to popliteal artery using prosthesis NEC |
| 7A482 | Bypass of femoral artery by anastomosis of femoral artery to popliteal artery using vein graft NEC |
| 7A483 | Bypass of popliteal artery by anastomosis of popliteal artery to popliteal artery using vein graft NEC |
| 7A484 | Bypass of femoral artery by anastomosis of femoral artery to tibial artery using prosthesis NEC |
| 7A485 | Bypass of popliteal artery by anastomosis of popliteal artery to tibial artery using prosthesis NEC |
| 7A486 | Bypass of femoral artery by anastomosis of femoral artery to tibial artery using vein graft NEC |
| 7A487 | Bypass of popliteal artery by anastomosis of popliteal artery to tibial artery using vein graft NEC |
| 7A488 | Bypass of femoral artery by anastomosis of femoral artery to peroneal artery using prosthesis NEC |
| 7A489 | Bypass of popliteal artery by anastomosis of popliteal artery to peroneal artery using prosthesis NEC |
| 7A48A | Bypass of femoral artery by anastomosis of femoral artery to peroneal artery using vein graft NEC |
| 7A48B | Bypass of popliteal artery by anastomosis of popliteal artery to peroneal artery using vein graft NEC |
| 7A48C | Bypass of femoral artery by anastomosis of femoral artery to femoral artery NEC |
| 7A48D | Bypass of popliteal artery by anastomosis of popliteal artery to femoral artery NEC |
| 7A48E | Femoro-femoral prosthetic cross over graft |
| 7A48y | Other specified other bypass of femoral artery or popliteal artery |
| 7A48z | Other bypass of femoral artery or popliteal artery NOS |
| 7A49. | Reconstruction of femoral artery or popliteal artery |
| 7A490 | Endarterectomy of femoral artery and patch repair of femoral artery |
| 7A491 | Endarterectomy of popliteal artery and patch repair of popliteal artery |
| 7A492 | Endarterectomy of femoral artery NEC |
| 7A493 | Endarterectomy of popliteal artery NEC |
| 7A494 | Profundoplasty of femoral artery and patch repair of deep femoral artery |
| 7A495 | Profundoplasty of popliteal artery and patch repair of popliteal artery |
| 7A496 | Profundoplasty of femoral artery NEC |
| 7A497 | Profundoplasty of popliteal artery NEC |
| 7A498 | Reconstruction of femoral artery with vein graft |
| 7A499 | Reconstruction of popliteal artery with vein graft |
| 7A49y | Other specified reconstruction of femoral artery or popliteal artery |
| 7A49z | Reconstruction of femoral or popliteal artery NOS |
| 7A4A. | Other open operations on femoral artery or popliteal artery |
| 7A4A0 | Repair of femoral artery NEC |
| 7A4A1 | Repair of popliteal artery NEC |
| 7A4A2 | Open embolectomy of femoral artery |
| 7A4A3 | Open embolectomy popliteal artery |
| 7A4A4 | Ligation of aneurysm of popliteal artery |
| 7A4A5 | Operation on aneurysm of femoral artery NEC |
| 7A4A6 | Operation on popliteal artery NEC |
| 7A4A7 | Repair of femoral artery with temporary silastic shunt |
| 7A4A8 | Repair of popliteal artery with temporary silastic shunt |
| 7A4Ay | Other specified other open operation on femoral artery or popliteal artery |
| 7A4Az | Other open operation on femoral artery or popliteal artery NOS |
| 7A4B. | Transluminal operations on femoral artery or popliteal artery |
| 7A4B0 | Percutaneous transluminal angioplasty of femoral artery |
| 7A4B1 | Percutaneous transluminal angioplasty of popliteal artery |
| 7A4B2 | Percutaneous transluminal embolectomy of femoral artery |
| 7A4B3 | Percutaneous transluminal embolectomy of popliteal artery |
| 7A4B4 | Percutaneous transluminal embolisation of femoral artery |
| 7A4B5 | Percutaneous transluminal embolisation of popliteal artery |
| 7A4B8 | Percutaneous transluminal thrombolysis of femoral graft using streptokinase |
| 7A4B9 | Percutaneous transluminal insertion of stent into femoral artery |
| 7A4By | Other specified transluminal operation on femoral artery or popliteal artery |
| 7A4Bz | Transluminal operation on femoral artery or popliteal artery NOS |
| 14N4. | H/O: limb amputation |
| 14N40 | H/O: upper limb amputation |
| 14N41 | H/O: lower limb amputation |
| 14N4Z | H/O: limb amputation NOS |
| 7L04. | Amputation of arm |
| 7L040 | Forequarter amputation |
| 7L041 | Disarticulation of shoulder |
| 7L042 | Amputation above elbow |
| 7L043 | Amputation through forearm |
| 7L044 | Amputation through elbow |
| 7L04y | Other specified amputation of arm |
| 7L04z | Amputation of arm NOS |
| 7L05. | Amputation of hand |
| 7L050 | Amputation through wrist |
| 7L051 | Amputation of thumb |
| 7L052 | Amputation of phalanx of finger |
| 7L053 | Amputation of finger NEC |
| 7L054 | Amputation through phalanx of finger |
| 7L055 | Terminalisation of thumb |
| 7L056 | Terminalisation of finger |
| 7L05y | Other specified amputation of hand |
| 7L05z | Amputation of hand NOS |
| 7L06. | Amputation of leg |
| 7L060 | Hindquarter amputation |
| 7L061 | Disarticulation of hip |
| 7L062 | Amputation above knee |
| 7L063 | Amputation through knee |
| 7L064 | Amputation below knee |
| 7L06y | Other specified amputation of leg |
| 7L06z | Amputation of leg NOS |
| 7L07. | Amputation of foot |
| 7L070 | Amputation through ankle |
| 7L071 | Disarticulation of tarsal bones |
| 7L072 | Disarticulation tarsometatarsal joint |
| 7L073 | Amputation through metatarsal bones |
| 7L07y | Other specified amputation of foot |
| 7L07z | Amputation of foot NOS |
| 7L08. | Amputation of toe |
| 7L080 | Amputation hallux |
| 7L081 | Amputation of phalanx of toe |
| 7L082 | Proximal hemiphalangectomy of toe |
| 7L083 | Amputation lesser toe |
| 7L084 | Terminalisation of hallux |
| 7L085 | Terminalisation of lesser toe |
| 7L08y | Other specified amputation of toe |
| 7L08z | Amputation of toe NOS |
| 7L09. | Operations on amputation stump |
| 7L090 | Reamputation at higher level |
| 7L091 | Excision of lesion of amputation stump |
| 7L092 | Shortening of amputation stump |
| 7L093 | Revision coverage of amputation stump |
| 7L094 | Drainage of amputation stump |
| 7L095 | Excision neuroma amputation stump |
| 7L09y | Other specified operation on amputation stump |
| 7L09z | Operation on amputation stump NOS |
| F336. | Phantom limb syndrome |
| F3360 | Phantom limb syndrome with pain |
| F3361 | Phantom limb syndrome without pain |
| SP16. | Late amputation stump complication |
| SP160 | Late amputation stump complication, unspecified |
| SP161 | Neuroma of amputation stump |
| SP162 | Chronic infection of amputation stump |
| SP163 | Necrosis of amputation stump |
| SP164 | Bony prominence in amputation stump |
| SP165 | Poorly shaped amputation stump |
| SP16z | Late amputation stump complication NOS |
| 16I.. | Claudication distance |
| G732. | Peripheral gangrene |
| G7320 | Gangrene of toe |
| G7321 | Gangrene of foot |
| G7322 | Gangrene of finger |
| G7323 | Gangrene of thumb |
| G7324 | Gangrene of hand |
| G733. | Ischaemic foot |
| G734. | Peripheral arterial disease |
| G73y. | Other specified peripheral vascular disease |
| G73y0 | Diabetic peripheral angiopathy |
| G73y1 | Peripheral angiopathic disease EC NOS |
| G73y2 | Acrocyanosis |
| G73yz | Other specified peripheral vascular disease NOS |
| G702. | Extremity artery atheroma |
| G7020 | Monckeberg's medial sclerosis |
| G702z | Extremity artery atheroma NOS |
| G73z. | Peripheral vascular disease NOS |
| G73z0 | Intermittent claudication |
| G73z1 | Spasm of peripheral artery |
| G73zz | Peripheral vascular disease NOS |

**Renal replacement**

| **Read code** | **Read code term** |
| --- | --- |
| 14S2. | H/O: kidney recipient |
| 14V2. | H/O: renal dialysis |
| 14V2. | H/O: kidney dialysis |
| 44yC1 | Peritoneal dialysis fluid adenosine deaminase level |
| 4I29. | Peritoneal dialysis sample |
| 4N... | Dialysis fluid examination |
| 4N0.. | Dialysis fluid urea level |
| 4N1.. | Dialysis fluid creatinine level |
| 4N2.. | Dialysis fluid glucose level |
| 4N3.. | Peritoneal dialysis fluid cell count |
| 4N4.. | Dialysis fluid potassium level |
| 4N5.. | Dialysis fluid sodium level |
| 7B00. | Transplantation of kidney |
| 7B001 | Transplantation of kidney from live donor |
| 7B001 | Allotransplantation of kidney from live donor |
| 7B002 | Transplantation of kidney from cadaver |
| 7B002 | Allotransplantation of kidney from cadaver |
| 7B002 | Cadaveric renal transplant |
| 7B003 | Allotransplantation of kidney from cadaver, heart-beating |
| 7B004 | Allotransplantation of kidney from cadaver, heart non-beating |
| 7B005 | Allotransplantation of kidney from cadaver NEC |
| 7B006 | Xenograft renal transplant |
| 7B00y | Other specified transplantation of kidney |
| 7B00z | Transplantation of kidney NOS |
| 7B012 | Bilateral nephrectomy |
| 7B015 | Transplant nephrectomy |
| 7B015 | Excision of rejected transplanted kidney |
| 7B019 | Excision of rejected transplanted kidney |
| 7B063 | Exploration of renal transplant |
| 7B0F3 | Post-transplantation of kidney examination, recipient |
| 7L1A. | Compensation for renal failure |
| 7L1A. | Dialysis for renal failure |
| 7L1A0 | Renal dialysis |
| 7L1A0 | Thomas intravascular shunt for dialysis |
| 7L1A1 | Peritoneal dialysis |
| 7L1A2 | Haemodialysis NEC |
| 7L1A4 | Automated peritoneal dialysis |
| 7L1A5 | Continuous ambulatory peritoneal dialysis |
| 7L1A6 | Peritoneal dialysis NEC |
| 7L1B. | Placement of ambulatory apparatus for compensation for renal failure |
| 7L1B. | Placement of ambulatory dialysis apparatus for compensation for renal failure |
| 7L1B0 | Insertion of ambulatory peritoneal dialysis catheter |
| 7L1B1 | Removal of ambulatory peritoneal dialysis catheter |
| 7L1B2 | Flushing of peritoneal dialysis catheter |
| 7L1By | Other specified placement of ambulatory apparatus for compensation for renal failure |
| 7L1Bz | Placement of ambulatory apparatus for compensation for renal failure NOS |
| 7L1C. | Placement of other apparatus for compensation for renal failure |
| 7L1Cy | Other specified placement of other apparatus for compensation for renal failure |
| 7L1Cz | Placement of other apparatus for compensation for renal failure NOS |
| 8882 | Intestinal dialysis |
| K0B5. | Renal tubulo-interstitial disorders in transplant rejection |
| PD000 | Bilateral renal agenesis |
| PD020 | Bilateral congenital absence of kidneys |
| SP015 | Mechanical complication of dialysis catheter |
| SP06B | Continuous ambulatory peritoneal dialysis associated peritonitis |
| SP083 | Kidney transplant failure and rejection |
| SP08a | Thrombosis of vein of transplanted kidney |
| SP08b | De novo glomerulonephritis |
| SP08C | Accelerated rejection of renal transplant |
| SP08D | Acute-on-chronic rejection of renal transplant |
| SP08E | Acute rejection of renal transplant - grade I |
| SP08F | Acute rejection of renal transplant - grade II |
| SP08G | Acute rejection of renal transplant - grade III |
| SP08H | Acute rejection of renal transplant |
| SP08J | Chronic rejection of renal transplant |
| SP08J | Chronic transplant nephropathy |
| SP08K | Chronic rejection of renal transplant - grade 1 |
| SP08L | Chronic rejection of renal transplant - grade II |
| SP08M | Chronic rejection of renal transplant - grade III |
| SP08N | Unexplained episode of renal transplant dysfunction |
| SP08P | Stenosis of vein of transplanted kidney |
| SP08Q | Aneurysm of artery of transplanted kidney |
| SP08R | Renal transplant rejection |
| SP08S | Aneurysm of vein of transplanted kidney |
| SP08T | Urological complication of renal transplant |
| SP08V | Very mild acute rejection of renal transplant |
| SP08V | Borderline changes of acute rejection |
| SP08W | Vascular complication of renal transplant |
| SP08X | Rupture of artery of transplanted kidney |
| SP08Y | Rupture of vein of transplanted kidney |
| SP08Z | Thrombosis of artery of transplanted kidney |
| SP0E. | Disorders associated with peritoneal dialysis |
| SP0E0 | Bloodstained peritoneal dialysis effluent |
| SP0E1 | Thrombus in peritoneal dialysis catheter |
| SP0F. | Haemodialysis first use syndrome |
| SP0G. | Anaphylactoid reaction due to haemodialysis |
| SP0H. | Disorder associated with dialysis |
| SP0H0 | Dialysis disequilibrium |
| SP3y9 | Acute hypercalcaemia of dialysis |
| TA020 | Accidental cut, puncture, perforation or haemorrhage during kidney dialysis |
| TA020 | Accidental cut, puncture, perforation or haemorrhage during renal dialysis |
| TA120 | Foreign object left in body during kidney dialysis |
| TA120 | Foreign object left in body during renal dialysis |
| TA220 | Failure of sterile precautions during kidney dialysis |
| TA220 | Failure of sterile precautions during renal dialysis |
| TA420 | Mechanical failure of instrument or apparatus during kidney dialysis |
| TA420 | Mechanical failure of apparatus during renal dialysis |
| TB001 | Transplantation of kidney as the cause of abnormal reaction of patient, or of later complication, without mention of misadventure at the time of operation |
| TB001 | Renal transplant with complication, without blame |
| TB11. | Kidney dialysis as the cause of abnormal reaction of patient, or of later complication, without mention of misadventure at the time of procedure |
| TB11. | Renal dialysis with complication, without blame |
| U6102 | [X]Unintentional cut, puncture, perforation or haemorrhage during kidney dialysis or other perfusion |
| U6112 | [X]Foreign object accidentally left in body during kidney dialysis or other perfusion |
| U6122 | [X]Failure of sterile precautions during kidney dialysis or other perfusion |
| U641. | [X]Kidney dialysis as the cause of abnormal reaction of the patient, or of later complication, without mention of misadventure at the time of the procedure |
| ZV420 | [V]Kidney transplanted |
| ZV56. | [V]Aftercare involving intermittent dialysis |
| ZV560 | [V]Aftercare involving extracorporeal dialysis |
| ZV560 | [V]Aftercare involving renal dialysis NOS |
| ZV56y | [V]Other specified aftercare involving intermittent dialysis |
| ZV56y | [V]Aftercare involving peritoneal dialysis |
| ZV56z | [V]Unspecified aftercare involving intermittent dialysis |

**Renal impairment**“eGFR” is determined from the maximum of eGFR_L1, eGFR_L1_90 +/- eGFR_L1_90_int. However, eGFR is “NA” if eGFR_L1_90 is NULL (i.e. there need to be at least 2x creatinine readings).

eGFR = 141 × min(SCr/K, 1)^a × max(SCr/K, 1)^-1.209 × 0.993^Age × 1.018 [if female] x 1.159 [if black ethnicity].

eGFR = 141 × min(SCr/K, 1)^a × max(SCr/K, 1)^-1.209 × 0.993^Age × 1.018 [if female] x 1.159 [if black ethnicity].

K = 0.7 for females, 0.9 for males

SCr = Serum creatinine

| **Read code** | **Read code term** |
| --- | --- |
| 44J3. | Serum creatinine |
| 44JF. | Plasma creatinine level |

**Ischaemic heart disease**

| **Read code** | **Read code term** |
| --- | --- |
| G3401 | Double coronary vessel disease |
| 792.. | Coronary artery operations |
| 7920 | Saphenous vein graft replacement of coronary artery |
| 79200 | Saphenous vein graft replacement of one coronary artery |
| 79201 | Saphenous vein graft replacement of two coronary arteries |
| 79202 | Saphenous vein graft replacement of three coronary arteries |
| 79203 | Saphenous vein graft replacement of four or more coronary arteries |
| 7920y | Other specified saphenous vein graft replacement of coronary artery |
| 7920z | Saphenous vein graft replacement coronary artery NOS |
| 7921 | Other autograft replacement of coronary artery |
| 79210 | Autograft replacement of one coronary artery NEC |
| 79211 | Autograft replacement of two coronary arteries NEC |
| 79212 | Autograft replacement of three coronary arteries NEC |
| 79213 | Autograft replacement of four of more coronary arteries NEC |
| 7921y | Other specified other autograft replacement of coronary artery |
| 7921z | Other autograft replacement of coronary artery NOS |
| 7922 | Allograft replacement of coronary artery |
| 79220 | Allograft replacement of one coronary artery |
| 79221 | Allograft replacement of two coronary arteries |
| 79222 | Allograft replacement of three coronary arteries |
| 79223 | Allograft replacement of four or more coronary arteries |
| 7922y | Other specified allograft replacement of coronary artery |
| 7922z | Allograft replacement of coronary artery NOS |
| 7923 | Prosthetic replacement of coronary artery |
| 79230 | Prosthetic replacement of one coronary artery |
| 79231 | Prosthetic replacement of two coronary arteries |
| 79232 | Prosthetic replacement of three coronary arteries |
| 79233 | Prosthetic replacement of four or more coronary arteries |
| 7923y | Other specified prosthetic replacement of coronary artery |
| 7923z | Prosthetic replacement of coronary artery NOS |
| 7924 | Revision of bypass for coronary artery |
| 79240 | Revision of bypass for one coronary artery |
| 79241 | Revision of bypass for two coronary arteries |
| 79242 | Revision of bypass for three coronary arteries |
| 79243 | Revision of bypass for four or more coronary arteries |
| 79244 | Revision of connection of thoracic artery to coronary artery |
| 79245 | Revision of implantation of thoracic artery into heart |
| 7924y | Other specified revision of bypass for coronary artery |
| 7924z | Revision of bypass for coronary artery NOS |
| 7925 | Connection of mammary artery to coronary artery |
| 79250 | Double anastomosis of mammary arteries to coronary arteries |
| 79251 | Double implantation of mammary arteries into coronary arteries |
| 79252 | Single anastomosis of mammary artery to left anterior descending coronary artery |
| 79253 | Single anastomosis of mammary artery to coronary artery NEC |
| 79254 | Single implantation of mammary artery into coronary artery |
| 7925y | Other specified connection of mammary artery to coronary artery |
| 7925z | Connection of mammary artery to coronary artery NOS |
| 7926 | Connection of other thoracic artery to coronary artery |
| 79260 | Double anastomosis of thoracic arteries to coronary arteries NEC |
| 79261 | Double implantation of thoracic arteries into coronary arteries NEC |
| 79262 | Single anastomosis of thoracic artery to coronary artery NEC |
| 79263 | Single implantation of thoracic artery into coronary artery NEC |
| 7926y | Other specified connection of other thoracic artery to coronary artery |
| 7926z | Connection of other thoracic artery to coronary artery NOS |
| 7927 | Other open operations on coronary artery |
| 79273 | Transposition of coronary artery NEC |
| 79275 | Open angioplasty of coronary artery |
| 7927y | Other specified other open operation on coronary artery |
| 7927z | Other open operation on coronary artery NOS |
| 7928 | Transluminal balloon angioplasty of coronary artery |
| 79280 | Percutaneous transluminal balloon angioplasty of one coronary artery |
| 79281 | Percutaneous transluminal balloon angioplasty of multiple coronary arteries |
| 79282 | Percutaneous transluminal balloon angioplasty of bypass graft of coronary artery |
| 79283 | Percutaneous transluminal cutting balloon angioplasty of coronary artery |
| 7928y | Other specified transluminal balloon angioplasty of coronary artery |
| 7928z | Transluminal balloon angioplasty of coronary artery NOS |
| 7929 | Other therapeutic transluminal operations on coronary artery |
| 79290 | Percutaneous transluminal laser coronary angioplasty |
| 79293 | Rotary blade coronary angioplasty |
| 79294 | Insertion of coronary artery stent |
| 79295 | Insertion of drug-eluting coronary artery stent |
| 79296 | Percutaneous transluminal atherectomy of coronary artery |
| 7929y | Other specified other therapeutic transluminal operation on coronary artery |
| 7929z | Other therapeutic transluminal operation on coronary artery NOS |
| 792A. | Diagnostic transluminal operations on coronary artery |
| 792A0 | Percutaneous transluminal angioscopy |
| 792Ay | Other specified diagnostic transluminal operation on coronary artery |
| 792Az | Diagnostic transluminal operation on coronary artery NOS |
| 792B. | Repair of coronary artery NEC |
| 792B0 | Endarterectomy of coronary artery NEC |
| 792By | Other specified repair of coronary artery |
| 792Bz | Repair of coronary artery NOS |
| 792C. | Other replacement of coronary artery |
| 792C0 | Replacement of coronary arteries using multiple methods |
| 792Cy | Other specified replacement of coronary artery |
| 792Cz | Replacement of coronary artery NOS |
| 792D. | Other bypass of coronary artery |
| 792Dy | Other specified other bypass of coronary artery |
| 792Dz | Other bypass of coronary artery NOS |
| 792E. | Percutaneous coronary intervention |
| 7.92E+02 | Emergency percutaneous coronary intervention |
| 792y. | Other specified operations on coronary artery |
| 792z. | Coronary artery operations NOS |

**Ischaemic stroke and transient ischaemic attack**

| **Read code** | **Read code term** |
| --- | --- |
| G63y0 | Cerebral infarct due to thrombosis of precerebral arteries |
| G63y1 | Cerebral infarction due to embolism of precerebral arteries |
| G64.. | Cerebral arterial occlusion |
| G640. | Cerebral thrombosis |
| G6400 | Cerebral infarction due to thrombosis of cerebral arteries |
| G641. | Cerebral embolism |
| G6410 | Cerebral infarction due to embolism of cerebral arteries |
| G64z. | Cerebral infarction NOS |
| G64z0 | Brainstem infarction |
| G64z1 | Wallenberg syndrome |
| G64z2 | Left sided cerebral infarction |
| G64z3 | Right sided cerebral infarction |
| G64z4 | Infarction of basal ganglia |
| G65.. | Transient cerebral ischaemia |
| G650. | Basilar artery syndrome |
| G651. | Vertebral artery syndrome |
| G6510 | Vertebro-basilar artery syndrome |
| G652. | Subclavian steal syndrome |
| G653. | Carotid artery syndrome hemispheric |
| G654. | Multiple and bilateral precerebral artery syndromes |
| G656. | Vertebrobasilar insufficiency |
| G657. | Carotid territory transient ischaemic attack |
| G65y. | Other transient cerebral ischaemia |
| G65z. | Transient cerebral ischaemia NOS |
| G65z0 | Impending cerebral ischaemia |
| G65z1 | Intermittent cerebral ischaemia |
| G65zz | Transient cerebral ischaemia NOS |
| G66..% | Stroke and cerebrovascular accident unspecified |
| G660. | Middle cerebral artery syndrome |
| G661. | Anterior cerebral artery syndrome |
| G662. | Posterior cerebral artery syndrome |
| G663. | Brain stem stroke syndrome |
| G664. | Cerebellar stroke syndrome |
| G665. | Pure motor lacunar syndrome |
| G666. | Pure sensory lacunar syndrome |
| G667. | Left sided CVA |
| G668. | Right sided CVA |
| G6760 | Cerebral infarction due to cerebral venous thrombosis, nonpyogenic |
| G6W.. | Cerebral infarction due to unspecified occlusion or stenosis of precerebral arteries |
| G6X.. | Cerebral infarction due to unspecified occlusion or stenosis of cerebral arteries |
| Gyu63 | [X]Cerebral infarction due to unspecified occlusion or stenosis of cerebral arteries |
| Gyu64 | [X]Other cerebral infarction |
| Gyu65 | [X]Occlusion and stenosis of other precerebral arteries |
| Gyu66 | [X]Occlusion and stenosis of other cerebral arteries |
| Gyu6G | [X]Cerebral infarction due to unspecified occlusion or stenosis of precerebral arteries |
| Fyu55 | [X]Other transient cerebral ischaemic attacks and related syndromes |
| Fyu56 | [X]Other lacunar syndromes |

**Atrial fibrillation**

| **Read code** | **Read code term** |
| --- | --- |
| 3272. | ECG: atrial fibrillation |
| 3273. | ECG: atrial flutter |
| 38DE. | Congestive heart failure, hypertension, age, diabetes, stroke 2 risk score |
| 38DE. | CHADS2 risk score |
| 38DE0 | Congestive heart failure, hypertension, age 2, diabetes mellitus, stroke 2, vascular disease, age, sex category score |
| 6A9.. | Atrial fibrillation annual review |
| 7936A | Implantation of intravenous pacemaker for atrial fibrillation |
| 8CMW2 | Atrial fibrillation care pathway |
| 8HTy. | Referral to atrial fibrillation clinic |
| 8OAD. | Provision of written information about atrial fibrillation |
| 9hF.. | Exception reporting: atrial fibrillation quality indicators |
| 9hF0. | Excepted from atrial fibrillation quality indicators: Patient unsuitable |
| 9hF1. | Excepted from atrial fibrillation quality indicators: Informed dissent |
| 9Os.. | Atrial fibrillation monitoring administration |
| 9Os0. | Atrial fibrillation monitoring first letter |
| 9Os1. | Atrial fibrillation monitoring second letter |
| 9Os2. | Atrial fibrillation monitoring third letter |
| 9Os3. | Atrial fibrillation monitoring verbal invite |
| 9Os4. | Atrial fibrillation monitoring telephone invite |
| G573. | Atrial fibrillation and flutter |
| G5730 | Atrial fibrillation |
| G5731 | Atrial flutter |
| G5733 | Non-rheumatic atrial fibrillation |
| G5734 | Permanent atrial fibrillation |
| G5735 | Persistent atrial fibrillation |
| G573z | Atrial fibrillation and flutter NOS |
| 662S. | Atrial fibrillation monitoring |
| 14AN. | H/O: atrial fibrillation |

**Retinopathy**

| **Read code** | **Read code term** |
| --- | --- |
| 2BBL | o/e diabetic maculopathy both eyes |
| 2BBT | O / E - R eye prolif diab ret (XaJOk) |
| 2BBV | O / E - L eye prolif diab ret |
| 2BBW | O / E - R eye diab maculopathy (XaJOn) |
| 2BBX | O / E - L eye diab maculopathy (XaJOo) |
| 2BBY | O/ E - Referable retinopathy |
| 2BBm | O / E - R clin sig macula oedema |
| 2BBn | O / E - left eye clinically significant macular oedema |
| 2BBo | O/E - sight threatening diabetic retinopathy |
| C10EP | Type 1 diabetes mellitus with exudative maculopathy |
| C10FQ | Type 2 diabetes meelitus with exudative maculopathy |
| F4201 | Proliferative diabetic retinopathy |
| F4203 | Advanced diabetic maculopathy |
| F4204 | Diabetic maculopathy |
| F4205 | Advanced diabetic retinal disease |
| F4207 | High risk proliferative diabetic retinopathy |
| 2BBY | O/ E - Referable retinopathy |
| F4208 | High risk non proliferative diabetic retinopathy |
| 2BBR | O / E - R eye preprolif diab ret (XaJOi) |
| 2BBS | O / E - L eye preprolif diab ret (XaJOj) |
| 2BBk | O/E - right eye stable treated prolif diabetic retinopathy |
| 2BBl | O/E - left eye stable treated prolif diabetic retinopathy |
| F4202 | Preproliferative diabetic retinopathy |
| 2BBO | O / E - Laser photocoag scars |
| 2BB7 | O / E - retinal vascular prolif. |
| F4200 | Background diabetic retinopathy |
| 2BBP | O / E - right eye back diab ret (XaJOg) |
| 2BBQ | O / E - left eye back diab ret (XaJOh) |
| 2BB4 | O / E - retinal microaneurysms |
| F4200 | Background diabetic retinopathy |
